# Cross-Electrophile Coupling of Benzyl Halides and Disulfides Catalyzed by Iron

**DOI:** 10.1021/jacs.3c13984

**Published:** 2024-02-12

**Authors:** Julius Semenya, Yuanjie Yang, Elias Picazo

**Affiliations:** Department of Chemistry, Loker Hydrocarbon Research Institute, University of Southern California, 837 Bloom Walk, Los Angeles, California 90089-1661, United States

## Abstract

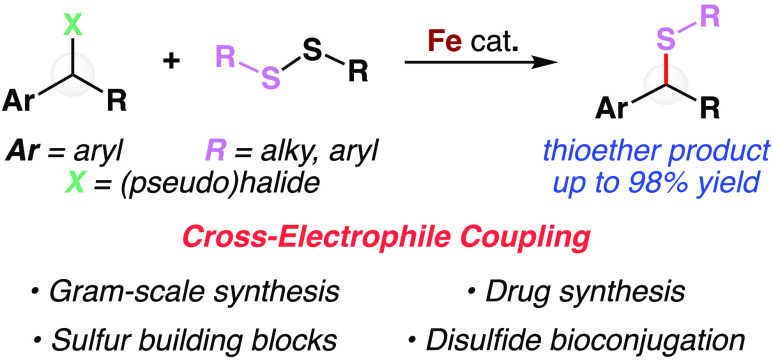

Cross-electrophile couplings are influential reactions that typically require a terminal reductant or photoredox conditions. We discovered an iron-catalyzed reaction that couples benzyl halides with disulfides to yield thioether products in the absence of a terminal reductant and under photoredox conditions. The disclosed platform proceeds without sulfur-induced catalyst poisoning or the use of an exogenous base, supporting a broad scope and circumventing undesired elimination pathways. We applied the developed chemistry in a new mode of disulfide bioconjugation, drug synthesis, gram-scale synthesis, and product derivatization. Lastly, we performed mechanistic experiments to better understand the stereoablative reaction between two electrophiles. Disulfides and benzylic thioethers are imperative for biological and pharmaceutical applications but remain severely understudied in comparison to their ethereal and amino counterparts. Hence, we expect this platform of iron catalysis and the downstream applications to be of interest to the greater scientific community.

## Introduction

The advent of transition-metal cross-coupling reactions has greatly facilitated molecular synthesis.^1^ Transition-metal cross-coupling reactions typically merge a nucleophilic coupling partner with an electrophilic coupling partner. Coupling two electrophilic components is met with the additional challenge of forming the cross-product dimer.^2^ Tremendous efforts in nickel catalysis,^3^ photoredox catalysis,^4,5^ and electrochemistry^6^ have resulted in various C(sp^2^)–C(sp^3^) and C(sp^3^)– C(sp^3^) couplings of two electrophilic components. Despite these advances, cross-electrophile couplings without the use of an exogenous reductant or photoredox conditions are rare. We wondered if nature’s example of using iron-containing enzymes to activate disulfides for target reduction could be leveraged to develop a new approach for cross-electrophile couplings between alkyl halides and disulfides. Such an advance provides a new mode of iron cross-coupling reactions and a mild means of synthesizing thioether products.

**Figure 1:**
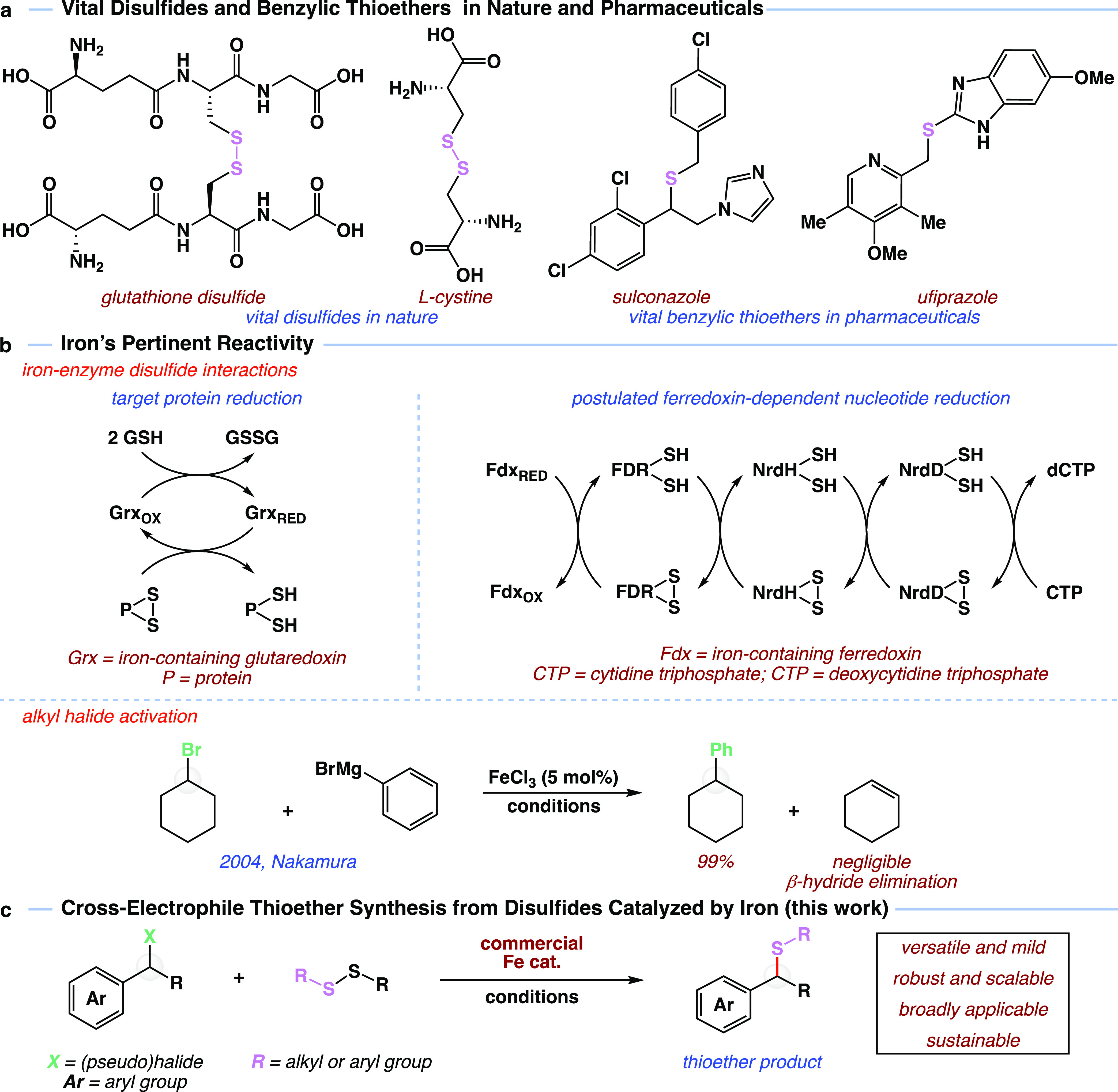
Cross-electrophile coupling of alkyl halides and disulfides catalyzed by iron. (a) Representative naturally occurring disulfides and benzylic thioethers in pharmaceutical molecules. (b) Pertinent reactivity, including enzymatic reactions involving iron and disulfides. (c) Cross-electrophile coupling catalyzed by iron to yield thioether products. Me, methyl group; GSH, glutathione; GSSG, glutathione disulfide; Grx, glutaredoxin; P, protein; Fdx, ferredoxin; FDR, ferredoxin disulfide reductase; NrdH, glutaredoxin-like redox protein; NrdD, ribonucleotide-triphosphate reductase; CTP, cytidine triphosphate; dCTP, deoxycytidine triphosphate; X, (pseudo)halide; Ar, aryl group; R, alkyl or aryl group.

Benzylic thioethers and disulfides are sulfur-based organic compounds that have been prevalent throughout the planet’s history and are essential macronutrients for organism development.^7^ Disulfides such as cystine and glutathione disulfide are critical bioindicators vital for cellular health (Figure 1a).^8^ As such, our ability to detect and react with disulfide bonds significantly advances our understanding of enzyme function, biological events, biodistributions, and imaging biomarkers.^9,10^ Further, an increasing impact of sulfur-containing therapeutics is instrumental to the evolution of therapeutics as benzylic thioethers and related scaffolds are used to treat cancer,^11^ tuberculosis,^12^ Alzheimer’s,^13^ malaria,^14^ and diabetes,^15^ among other medical conditions.^16^ Nearly 25% of small-molecule drugs within the top 200 drugs by retail sales and prescriptions in the U.S. contain this heteroatom, with 8.8% of those compounds possessing a thioether functional group.^17^ Lastly, benzylic thioethers are valuable chemical reagents and building blocks used in an assortment of reactions, polymers, and biological applications.^18^ The significant roles that thioether and disulfide compounds play in biological processes, synthesis, pharmaceuticals, and functional materials demand the development of reactions for their detection and preparation.^19^ Despite the importance of benzylic thioethers and disulfides in numerous fields, catalytic reactions coupling disulfides and alkyl halide starting materials remain elusive. Strategies involving transition-metal catalysts are further complicated by known incompatibilities that lead to catalyst poisoning by thiolate species.^20^

Nature utilizes iron-containing enzymes such as glutaredoxin (Grx) and ferredoxin (Fdx) in a series of radical reactions for direct disulfide activation in target protein^21^ or nucleoside^22^ reduction (Figure 1b). Further, seminal efforts in developing and understanding iron-catalyzed photoredox reactions,^23^ cross-coupling reactions,^24,25^ and biological transformations^26^ emphasize iron’s synthetic versatility^27,28^ through a variety of reaction pathways with little to no β-hydride elimination (Figure 1b, bottom). We hypothesized that nature’s ability to react iron with disulfides^29^ and iron’s ability to activate alkyl halides^30^ could be leveraged to develop reaction conditions for the detection of disulfides and subsequent formation of valuable carbon–sulfur bonds through a cross-electrophile coupling.

In addition to the unique reactivity positioning iron to solve this synthetic challenge, iron catalysts are of particular interest for their ability to address aspects of sustainability.^31,32^ Iron is the most abundant transition metal in the earth’s crust, and as a result, a variety of iron catalysts varying in oxidation states are readily available. As an essential element for deoxyribonucleic acid (DNA) synthesis, oxygen transport, and electron transport, among other metabolic processes,^33,34^ iron is also vital to most living organisms and lowly toxic.^35^

Current synthetic methods for thioether synthesis are limiting^36^ with methods primarily relying on harsh basic or acidic conditions,^37,38^ giving rise to undesired byproducts. Specifically, substrates containing homobenzylic protons are vulnerable to elimination reaction pathways under such conditions. Here, we report the successful realization of an iron-catalyzed cross-electrophile coupling of benzyl halides with disulfide reagents to form thioether products (Figure 1c). Conditions avoid the use of caustic reagents, and elimination pathways are prevented. Further, no undesired homocoupling or other byproducts arising from hydrogen-atom abstraction were observed. Generality of the presented transformation was assessed through an extensive substrate scope, varying multiple components in the benzyl halide and disulfide coupling partners. Synthetic applications showcase scalability, versatility, and downstream applications of the reported technology including product elaboration, drug synthesis, and a new mode of bioconjugation. The reported method marks a significant advance to the limited alternative transition-metal or post-transition-metal procedures requiring the use of stoichiometric reagents^39,40^ or advancing only with activated coupling partners.^41,42^

## Results and Discussion

Based on iron-containing enzymatic reactions involving disulfide bonds^21,22,26^ and significant efforts to understand elementary steps involved in iron-catalyzed cross-coupling reactions,^24,25,27,28,43,44^ we hypothesized that a simple, commercial source of iron could activate a benzyl halide and subsequently react with disulfide reagents to yield valuable thioether products. In this vein, we tested the proposed coupling reaction using (1-bromoethyl)benzene (**1**), 5 mol % of iron(III) bromide, and a disulfide **2** in a variety of common organic solvents in efforts to produce thioethers **3** (Table 1).

**Table 1:**
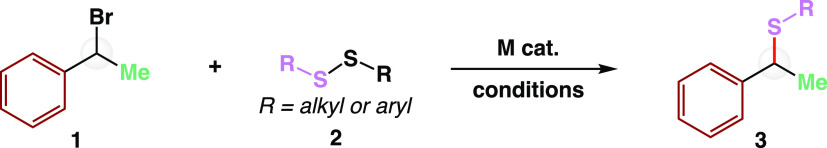

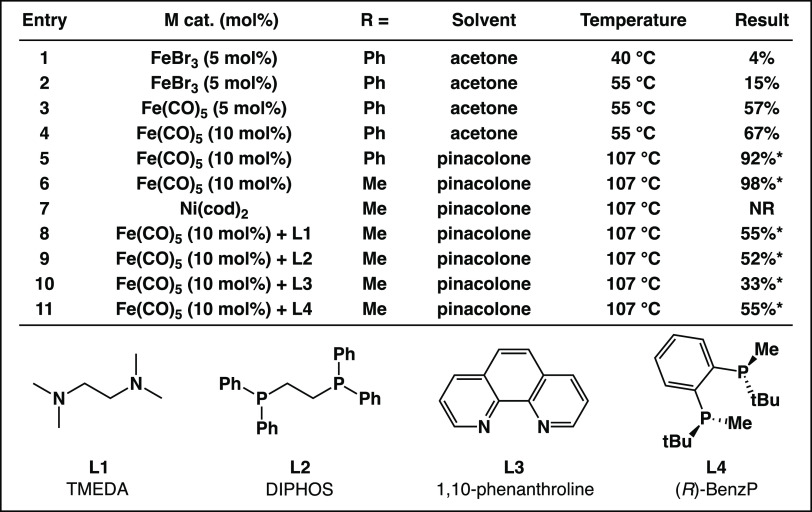
Discovery and Optimization Highlights for the Cross-Electrophile Coupling of Alkyl Halides and Disulfides Catalyzed by Iron^a^

a Various iron sources catalyze the transformation, alternative metal catalysts suffer, ligands and additives that typically facilitate iron catalysis proved detrimental. For full optimization and additive effects, including alternative metal evaluations, see Tables S1– S4 in the Supporting Information. *Isolated yields. Me, methyl group; Ph, phenyl group; M cat., metal catalyst; R, alkyl or aryl group; cod, cyclooctadiene; NR, no reaction; TMEDA, tetramethylethylenediamine; DIPHOS, 1,2-bis(diphenylphosphino)ethane; and (*R*)-BenzP, 1,2-Bis((*R*)-tert-butyl(methyl)phosphino)benzene.

After encountering considerable nonspecific decomposition and undesired reactivity, acetone was identified as an effective solvent to engage both the benzyl halide and disulfide coupling partners without decomposition or solvent incorporation (Table S1). Increasing the reaction temperature from 40 to 55 °C improved the reaction from 4 to 15% yield (Table 1, entries 1 and 2). It was also learned that numerous commercial iron sources promoted the desired transformation in acetone (Table S2). Iron pentacarbonyl, Fe(CO)_5_, was identified as the optimal source, providing 57% product when using 5 mol % of catalyst (Table 1, entry 3). Increasing catalyst loading from 5 to 10 mol % resulted in an increase from 57 to 67% product formation (Table 1, entry 4). Given that a carbonyl solvent and higher temperatures were advantageous, pinacolone was selected as the optimal solvent, and reaction at 107 °C yielded the product in 92% isolated yield (Table 1, entry 5). Dialkyl disulfides such as dimethyl disulfide could also undergo the reaction to efficiently produce dialkyl thioether products in high yield (Table 1, entry 6). The remaining mass balance was starting material, and no undesired byproducts, including elimination byproducts from homobenzylic deprotonation, were observed. Control experiments show that there is no reaction in the absence of iron. Irradiation with blue LEDs, in the absence or presence of iron, has no impact on product formation (Table S2, entries 7–9).

Given nickel’s ability to (1) access single- and two-electron pathways and (2) participate in elementary cross-coupling steps, nickel was tested in the discovered reaction. To our surprise, bis(cyclooctadiene)nickel(0) did not produce any desired product (Table 1, entry 7). Other metal catalysts such as magnesium, aluminum, copper, and palladium also proved ineffective in comparison to iron (Table S3). Further, ligands (Table 1, entries 8–11), common additives that generally facilitate iron-catalyzed reactions,^24,25,27,28,43,44^ and inorganic bases all proved detrimental to the iron-catalyzed cross-electrophile coupling reaction. Upon identification of optimal reaction conditions, all effects, time, substrate classes, and catalyst loadings were re-examined (Table S3, entries 9–24). Although this system is currently limited to benzylic (pseudo)halide substrates (see Supporting Information, Table S5), it is the only known example of a coupling methodology between alkyl halides and disulfides. For extensive optimization, substrate class, and additive studies, see Tables S1–S5 in the Supporting Information.

**Figure 2:**
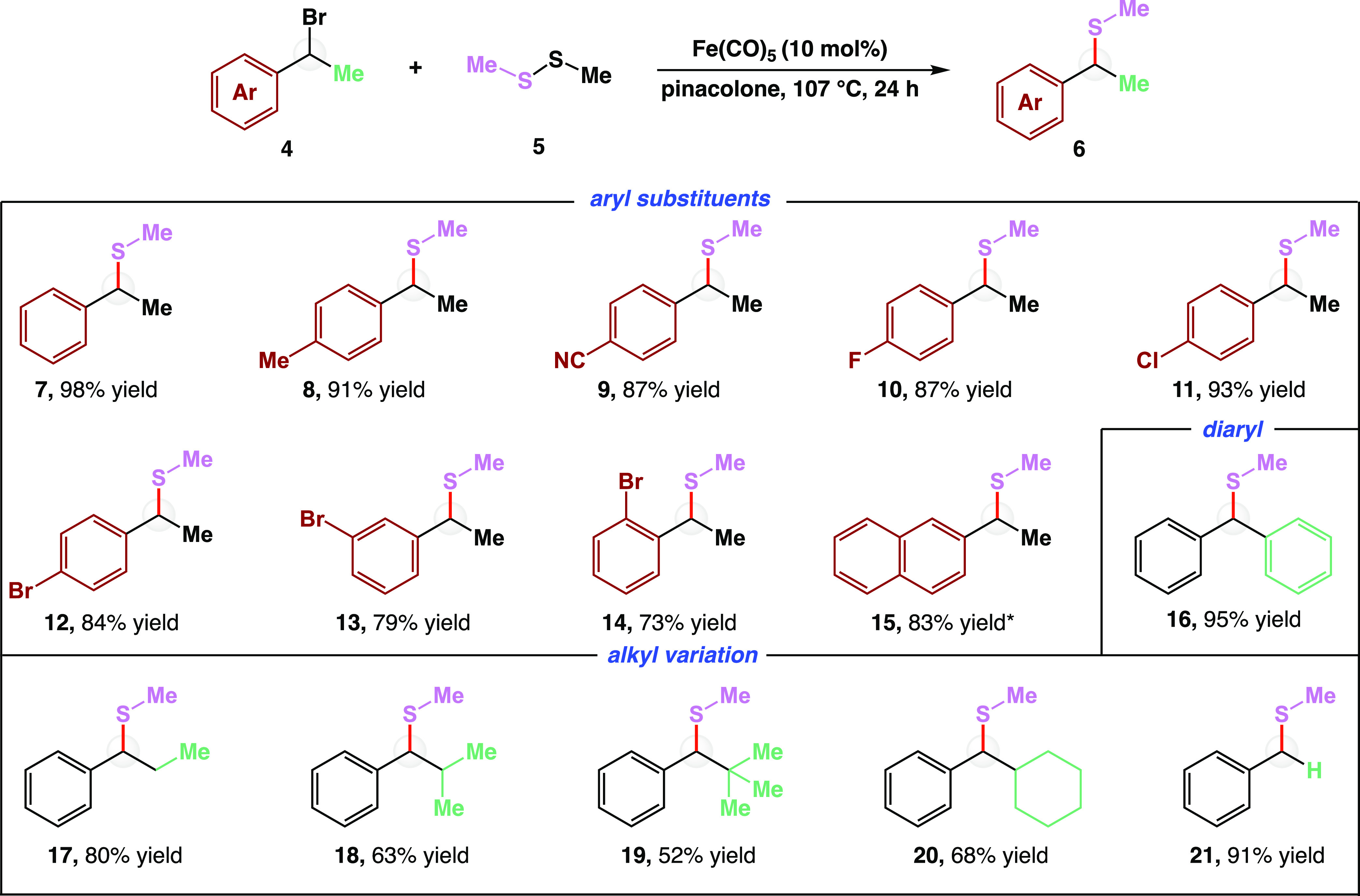
Bromide coupling partner scope in cross-electrophile coupling. Reactions were carried out on a 0.5 mmol scale of bromides **4** with 1.5 equiv of disulfide **5** and 10 mol % of Fe(CO)_5_ in pinacolone (1.0 mL) for 24 h at 107 °C. All yields are isolated yields. See the Supporting Information for detailed reaction conditions. Ar, aryl group; Me, methyl group; Fe(CO)_5_, iron pentacarbonyl. *Crude benzyl bromide used; two-step yield is reported.

Encouraged by these results, we studied the generality of the iron-catalyzed disulfide coupling reaction. As shown in Figure 2, a wide range of functional groups and aryl substituents were well-suited for the reaction between benzyl bromides **4** and dimethyl disulfide (**5**) in the presence of Fe(CO)_5_ to yield thioether products **6**. For example, unsubstituted and electron-rich 4-methyl substituted thioethers **7** and **8** formed smoothly with isolated yields of 98 and 91%, respectively. We learned that electron-withdrawing groups were also not burdensome to the iron-catalyzed transformation, as exemplified by the synthesis of cyano- and fluorothioether products **9** and **10** in 87% yield. High yields were observed for substrates substituted with historically metal-reactive groups, illustrated by fluoro-, chloro-, and bromo-adducts **10**–**14**. In contrast to other transition-metal systems,^45,46^ this protocol was selective for the benzylic bromide, leaving aryl halides untouched. Also noteworthy is that 4-, 3-, and 2-substitution of the arene was largely inconsequential, demonstrated by the productive synthesis of thioethers **12**–**14**. The slightly lower yields for **13** and **14** are presumably due to the steric effect of the arene substitution pattern. Additionally, extension of the conjugated system was not detrimental to the reaction, as demonstrated by the synthesis of thioether **15** in 83% yield and the reaction remains operable with diaryl bromide substrates, demonstrated with the synthesis of **16** in 95% yield. A clear steric effect is observed when increasing bulk from the methyl group present in product **7** (Figure 2, bottom). Ethyl adduct **17** was isolated in 80% yield, isopropyl derivative **18** was produced in 63% yield, and *tert*-butyl product **19** was achieved in 52% yield. Consistent with the steric trend, cyclohexyl derivative **20** was prepared in a 68% yield. The reaction proved to work well with primary bromides, highlighted by the synthesis of primary thioether **21** in 91% yield.

**Figure 3:**
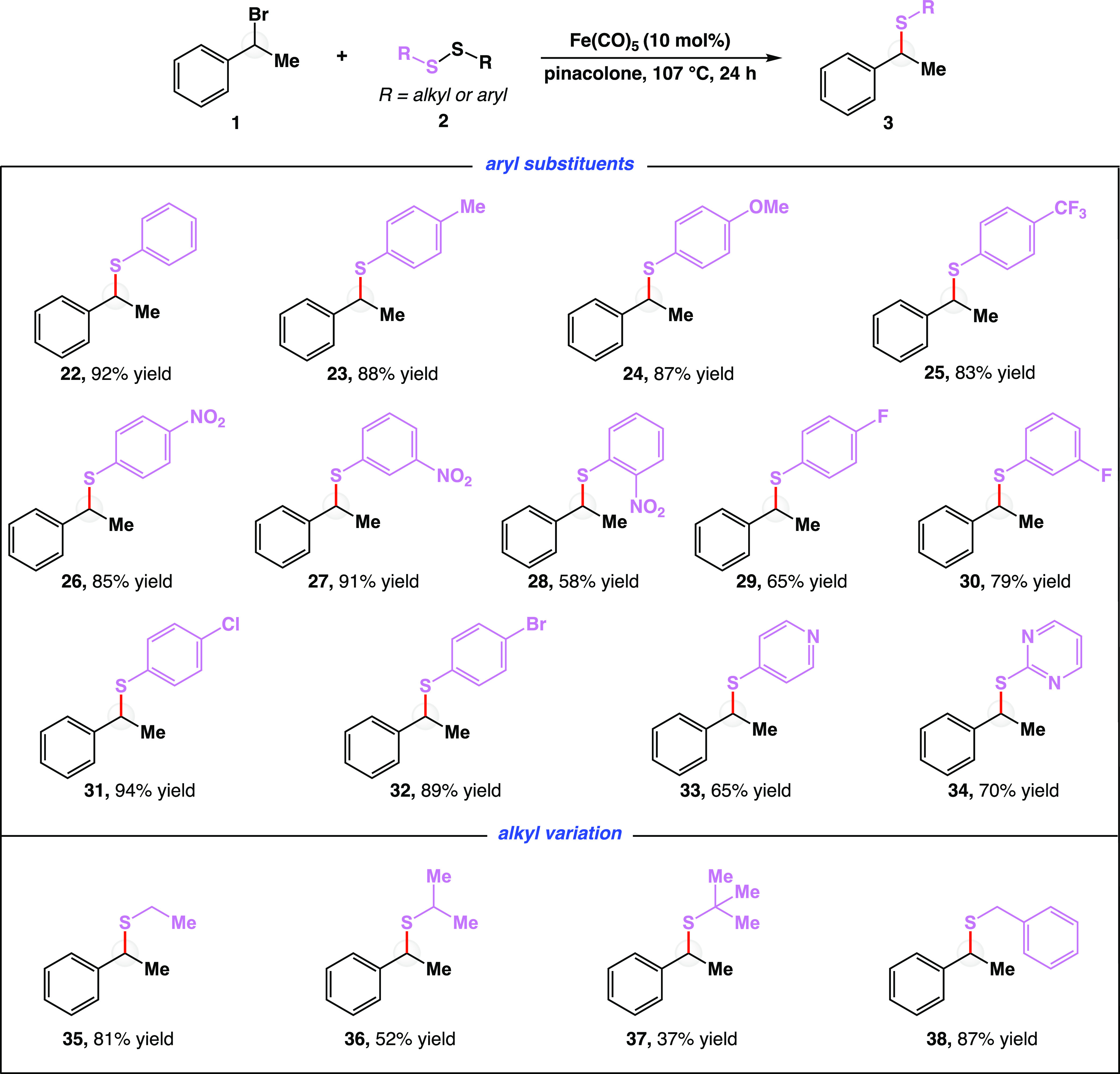
Disulfide coupling partner scope in cross-electrophile coupling. Reactions were carried out on a 0.5 mmol scale of bromide **1** with 1.5 equiv of disulfides **2** and 10 mol % of Fe(CO)_5_ in pinacolone (1.0 mL) for 24 h at 107 °C. All yields are isolated yields. See the Supporting Information for detailed reaction conditions. R, alkyl or aryl group; Me, methyl group; Fe(CO)_5_, iron pentacarbonyl.

As depicted in Figure 3, both dialkyl and diaryl disulfides **2** with varying electronic profiles are applicable. Phenyl thioether **22** was synthesized in 92% yield, while electron-rich products such as 4-methyl compound **23** and 4-methoxy adduct **24** were isolated in 88 and 87% yield, respectively. Electron-withdrawn trifluoromethyl thioether **25** and nitro product **26** were synthesized in 83 and 85% yield, respectively. Disulfide substrates could also bear 4-, 3-, or 2- substitutions on the arene, as demonstrated by products **26**–**28**. The slight decrease in reaction efficacy when moving from 4- and 3-substitution to 2-substitution is presumably due to the steric influence provided by the arene substituent. Halogenated diaryl disulfides yielded fluorinated compounds **29** and **30** in 65 and 79% yield, along with chlorinated and brominated thioethers **31** and **32** in 94 and 89% yield, respectively. The halogenated examples emphasize the orthogonality of this protocol to other transition-metal-catalyzed systems, as iron selectively activated the benzylic bromide while leaving the aryl halides unreacted. Inclusion of nitrogen within the aromatic backbone afforded pyridine and pyrimidine products **33** and **34** in 65 and 70% yield, respectively. The yields for pyridine- and pyrimidine-containing thioethers **33** and **34** are thought to have a lower yield due to iron coordination, which is consistent with our examination of ligand effects (for more detailed information on ligand effects, see Table S4). Increasing the steric profile of disulfide **2** resulted in decreased yields under identical reaction conditions (Figure 3, bottom). Specifically, moving from dimethyl disulfide (**5**) to diethyl disulfide decreased the reaction efficiency from 98 to 81% in the formation of thioethers **7** and **35**, respectively. Further, increasing to diisopropyl disulfide generated isopropyl thioether **36** in 52% yield while increasing to di-*tert*-butyl yielded thioether **37** in 37% yield. Consistent with our steric analysis and ability to synthesize both aryl and alkyl thioether products, benzyl thioether **38** was produced in 87% yield.

**Figure 4:**
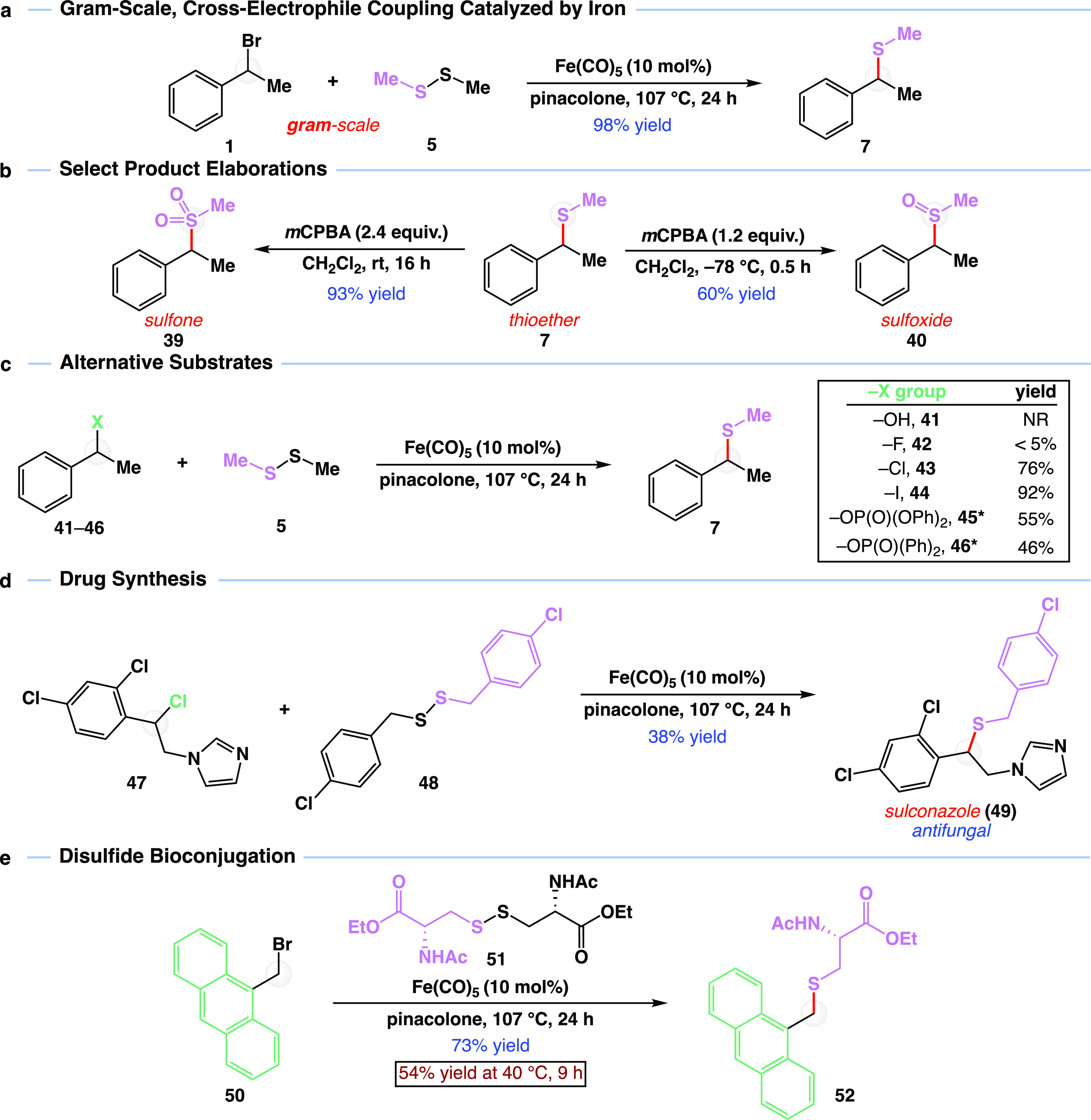
Direct and downstream synthetic applications of cross-electrophile coupling. (a) Gram-scale operation. Reaction was carried out on 1 g (5.4 mmol) of bromide **1**. (b) Synthesis of sulfoxides, sulfones, and thiols from reaction products. (c) Thioether synthesis catalyzed by iron with other halide and pseudohalide starting materials. (d) Application in drug synthesis. (e) Application in bioconjugation. Me, methyl group; Fe(CO)_5_, iron pentacarbonyl; *m*CPBA, *meta*-chloroperoxybenzoic acid; Ph, phenyl group; −OP(O)(OPh)_2_, diphenyl phosphate; −OP(O)(Ph)_2_, diphenyl phosphinate; NR, no reaction; Et, ethyl group; Ac, acetyl group. *Crude benzyl (pseudo)halide used; two-step yield is reported. All yields are isolated yields. See the Supporting Information for detailed reaction conditions.

With a robust reaction in hand, we next turned our attention to synthetic applications (Figure 4). One critical advantage of using iron as a catalyst to develop new platforms of reactivity is the low cost, low toxicity, and sustainable nature of iron.^32,35^ Iron has been successfully used in practical, kilogram-scale industrial applications,^31^ corroborating iron’s potential to address key challenges of sustainability in chemical synthesis. As such, we increased the reaction magnitude to gram-scale. Reacting 1 g of (1-bromoethyl)benzene (**1**) with dimethyl disulfide (**5**) and Fe(CO)_5_ in pinacolone at 107 °C for 24 h provided methyl thioether **7** in 98% yield (Figure 4a).

Second, given the high importance and value of sulfur-containing molecules, we sought to derivatize the products from the developed iron-catalyzed disulfide coupling reaction (Figure 4b). Specifically, sulfoxides and sulfones are found in biologically active molecules, act as ligands in transition-metal catalysis,^47^ and enable a variety of transformations including Mislow–Evans rearrangements,^48^ Pummerer reactions,^49^ Julia olefinations,^50^ and Ramberg–Backlund rearrangements,^51^ among others. In one example, methyl thioether **7** was oxidized with 2.4 equiv of *meta*-chloroperoxybenzoic acid (*m*CPBA) to deliver methyl sulfone **39** in 93% yield. In a second example, thioether **7** was oxidized with 1.2 equiv of *m*CPBA to deliver methyl sulfoxide **40** in 60% yield as a mixture of diastereomers.

Due to variable stabilities of leaving groups,^52^ we wondered how different halide and pseudohalides would perform in the reaction (Figure 4c). No reaction was observed when benzylic alcohol **41** was subjected to standard reaction conditions. Negligible conversion was detected when employing benzylic fluoride **42**. Consistent with bond energies, chloride substrate **43** yielded thioether **7** in 76% yield while benzylic iodide **44** provided the product in 92% yield. Activated alcohols in the form of diphenyl phosphate **45** and diphenyl phosphinate **46** were also active in the reaction, yielding product **7** in 55 and 46% yield, respectively. The lower yields for diphenyl phosphate **45** and diphenyl phosphinate **46** are presumed to be a result of using the coupling partners directly upon synthesis without further purification (see Table S6).

Further, because of the prevalence of benzylic thioethers in pharmaceutically active compounds, we sought to synthesize sulconazole, an antifungal medication of the imidazole class, using our method. In this vein, known benzyl chloride **47** was reacted with disulfide **48** to yield the desired antifungal thioether **49** in 38% yield (Figure 4d), emphasizing the ability to rapidly generate a library of biologically active molecules from common precursors. Lastly, given that this reaction was inspired by natural processes involving iron-containing enzymes, we wondered if the reaction could be used to cleave a cysteine-derived disulfide in reaction with cystine derivative **51**. The ability to productively cleave S–S bonds in cystine and related substrates, such as disulfide **51**, provides a new means of labeling peptides and proteins.^53^ A commonly used bridging protocol requires disulfide bond cleavage with reducing agents.^54^ Unfortunately, having two free thiols provides sufficient flexibility for structurally sensitive proteins to begin unfolding. Hence, we anticipate that our iron-catalyzed disulfide coupling reaction could be used as a new means of disulfide bioconjugation, wherein the disulfide linkage is cleaved and immediately trapped. With this application in mind, we subjected disulfide **51** and anthracenyl bromide **50** to standard reaction conditions and isolated desired bioconjugated thioether **52** in 73% yield (Figure 4e). Gratifyingly, the reaction could also be run at 40 °C for 9 h to yield bioconjugated thioether **52** in 54% yield. Primary bromide **50** was used to avoid complex mixtures of diastereomers, and the anthracenyl core in **50** was selected for the bioconjugation application because it is commonly used as a fluorophore.^55,56^

**Figure 5:**
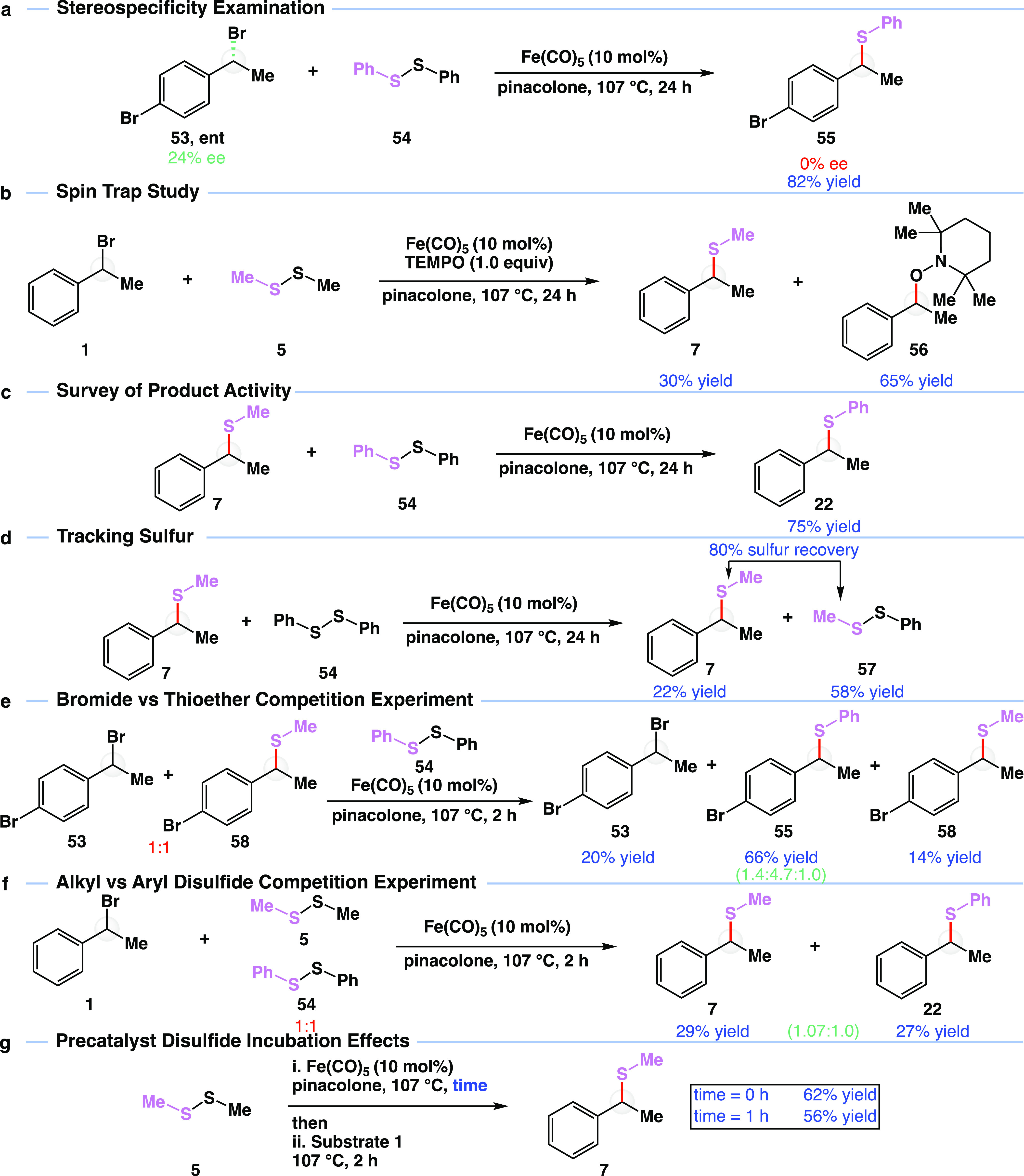
Mechanistic studies of cross-electrophile coupling. (a) Stereospecificity examination. Yield is isolated yield. (b) Spin trap study. Yields are isolated yields. (c) Survey of product activity. Yield is the NMR yield. (d) Tracking sulfur. Yield is NMR yield. (e) Bromide vs thioether competition experiment. Yields are NMR yields. (f) Alkyl vs aryl disulfide competition experiment. Yields are NMR yields. (g) Precatalyst disulfide incubation effects. Yields are NMR yields. Me, methyl group; Ph, phenyl group; Fe(CO)_5_, iron pentacarbonyl; ee, enantioenrichment; TEMPO, (2,2,6,6-tetramethylpiperidin-1-yl)oxyl. All reactions were performed on 0.5 mmol scale. See the Supporting Information for detailed reaction conditions.

The unprecedented nature and efficiency of the cross-electrophile coupling prompted a series of experiments to better understand aspects of the reaction (Figure 5). As previously discussed, ligands that conventionally facilitate iron-catalyzed reactions were detrimental to our cross-electrophile coupling. We further learned that despite using chiral ligands such as **L4**, products were produced in racemic form. Given that starting bromides were all racemic, we wondered if the reaction was producing racemic thioether products through a stereospecific or stereoablative pathway. To answer this, enantioenriched bromide **53, ent** was prepared in 24% enantiomeric excess (ee) and reacted with diphenyl disulfide (**54**) to produce thioether **55** in 82% yield as a racemate (Figure 5a). The deterioration of stereochemical information is consistent with a stereoablative pathway via a carbocation or radical intermediate. Due to the lower yields observed when using aluminum or magnesium catalysts, our initial hypothesis was that ee was being lost through the formation of a radical intermediate. This hypothesis was further substantiated with a radical spin experiment (Figure 5b). Reacting bromide **1** with dimethyl disulfide **5** in the presence of (2,2,6,6-tetramethylpiperidin-1-yl)oxyl (TEMPO) resulted in 30% thioether **7** formation and 65% TEMPO-adduct **56** formation. To further support an ablative mechanism, we sought to rule out a reactive product (Figure 5c). To our surprise, we learned that the thioether product **7** was itself active in the iron-catalyzed reaction. Specifically, reacting thioether **7** with diphenyl disulfide (**54**) resulted in the formation of phenyl thioether **22** in 75% yield. Hence, a molecule’s loss of ee may be a result of reaction between thioether product and excess disulfide.

We suspected that the bromide in **7** was undergoing a formal oxidation to form a sulfenyl bromide byproduct to balance the formal reduction experienced by one sulfur unit of the disulfide when forming the thioether product. Given the reactive nature of sulfenyl bromides, we were unable to isolate such intermediate. Using surprisingly active thioether **7** to our advantage, however, we found support for such a mechanistic redox hypothesis (Figure 5d). Specifically, focusing on the sulfur unit rather than the aryl unit of thioether **7**, the reaction with diphenyl disulfide (**54**) resulted in 22% thioether **7** and 58% yield of mixed disulfide **57**. Together, 80% of the sulfur unit input is accounted for. Hence, by analogy, bromide is acting as an internal reducing agent.

Interested in the active nature of various components, we conducted competition experiments. First, we evaluated the relative rates of bromide and thioether consumption (Figure 5e). Reacting a 1:1 mixture of bromide **53** and thioether **58** with diphenyl disulfide (**54**) for two hours resulted in the formation of a 1.4:4.7:1.0 mixture of **53**:**55**:**58**, respectively. Evidently, thioether coupling partners are consumed at a slightly higher rate. Further, we evaluated the relative rates of alkyl and aryl disulfide consumption (Figure 5f). Specifically, bromide **1** was reacted with a 1:1 mixture of dimethyl disulfide (**5**) and diphenyl disulfide (**54**) for two hours to yield either methyl thioether **7** or phenyl thioether **22**. A 1.07:1.0 ratio of **7**:**22** was observed, consistent with a similar rate of consumption slightly favoring dimethyl disulfide (**5**).

Due to the role of iron–sulfur clusters in iron–sulfur proteins, we wondered if iron was first interacting with the disulfide reagent to form an active iron–sulfur species. To test if there was an incubation period prior to catalysis, we added bromide **1** to a mixture of iron pentacarbonyl and dimethyl disulfide (**5**) that had been stirred at 107 °C for 1 h prior to the addition of bromide **1**. The reaction provided thioether **7** in 56% yield after 2 h of stirring, which compares to the control of 62% yield, consistent with iron either first interacting with substrate or iron having a fast first interaction with the disulfide. We also attempted reaction with a redox active ester as a substrate (in place of bromide substrate); however, this reaction was unsuccessful and will be subject to further investigation.^57^ Together with the (pseudo)halide studies described in Figure 4c and metal studies, these results are consistent with an Fe(0) oxidative cleavage event between iron and the (pseudo)halide substrate.^58^

## Conclusions

In summary, we discovered an iron-catalyzed cross-electrophile reaction that couples benzyl halides with disulfides in the absence of a terminal reductant and photoredox conditions. The system provides a practical and general route to benzylic thioethers without the use of caustic reagents and with no observable byproducts. The chemistry is amenable to a new mode of disulfide bioconjugation, drug synthesis, gram-scale synthesis, a broad halide and disulfide scope, and the preparation of additional valuable sulfur-containing molecules. Studies also provide preliminary mechanistic insight, highlighting a stereoablative pathway that likely involves an oxidative cleavage event between iron and the (pseudo)halide substrate, single electron steps, a reaction-active thioether product, and an internal reductant in the form of bromide. Additional reactions based on these findings are currently under development.

## Supporting Information Available

Detailed experimental procedures and compound characterization data (PDF)
