# Supplementary material for: Cross-Electrophile Coupling of Benzyl Halides and Disulfides Catalyzed by Iron
Source: J Am Chem Soc. 2024 Feb 12;146(7):4903–12. doi: 10.1021/jacs.3c13984 (PMC10910570; doi:10.1021/jacs.3c13984)
Supplement: Supplementary file 1 — ja3c13984_si_001.pdf [file ja3c13984_si_001.pdf]

Cross-Electrophile Coupling of Benzyl Halides and Disulfides Catalyzed by Iron

Julius Semenya, Yuanjie Yang, Elias Picazo*

Loker Hydrocarbon Research Institute and Department of Chemistry, University of Southern California, 837 Bloom Walk, Los Angeles, California 90089-1661, United States

Supporting Information – Table of Contents

1. **General Considerations** **S2**
2. **General procedure for reaction discovery** **S4**
3. **General procedure for reaction optimization** **S5**
4. **General procedure for metal evaluation** **S6**
5. **General procedure for additive evaluation** **S8**
6. **Failed substrate classes** **S9**
7. **General, optimized procedure for iron-catalyzed thioether synthesis** **S9**
8. **Disulfide synthesis and characterization** **S10**
9. **Bromide synthesis and characterization** **S14**
10. **Product characterization** **S27**
11. **Synthetic application procedures** **S44**
12. **Mechanistic analysis procedures** **S51**
13. **^{1}H, ^{13}C, and ^{19}F NMR data** **S58**
14. **SFC data** **S88**
15. **References** **S90**

Materials and Methods

1. General Considerations

Unless stated otherwise, reactions were conducted in flame-dried glassware under an atmosphere of nitrogen using anhydrous solvents (freshly distilled or passed through activated alumina columns). All commercially obtained reagents were used as received unless otherwise specified. All work-up and purification procedures used reagent grade solvents purchased from Fisher, VWR or Sigma-Aldrich. Reagents and catalyst used were purchased from the following vendors and used as received: diphenyl disulfide, iron (III) acetylacetonate, triphenyl phosphine, carbon tetrabromide, bis(cyclooctadiene)nickel(0), phenylethanol, pinacolone, dimethyl disulfide, phenylmagnesium chloride, ethylmagnesium chloride, triethylamine cylcohexylmagnesium chloride, magnesium chloride, aluminum chloride, palladium (II) chloride, tris(dibenzylideneacetone)dipalladium(0), benzyl bromide, 4-chlorobenzaldehyde, tetrakis(triphenylphosphine)nickel(0), phenylacetic acid, benzaldehyde, pivaldehyde, 2-nitro disulfide, methylmagnesium chloride, naphthalen-2-yl-ethanol, 2-methyl-1-phenylpropanone, and tetrakis(triphenylphosphine)palladium(0) were purchased from Sigma Aldrich. Iron (II) chloride, iron(III) bromide, iron (0) pentacarbonyl, chlorobenzenethiol, dibenzyl disulfide, 4-dimethylaminopyridine, 4-methoxy disulfide, benzhydryl bromide, and copper (II) bromide were purchased from Alfa Aesar. Bromoethylbenzne, 2-bromobenzaldehyde, 3-bromobenzaldehyde, 4-bromophenylethanol, 4-acetylbenzonitrile, *p*-tolyl disulfide, 4-nitrodisuldie, 4-fluorodisulfide, 3-fluorodisulfide, diethyl disulfide, N-hydroxyphthalimide, isopropyl disulfide, and *t*-butyl disulfide were purchased from TCI chemicals. N,N'-diisopropylcarbodiimide was acquired from Advanced ChemTech. Anthracenyl bromide, pyridine disulfide, 1-bromoethyl-4-fluorobenzene, iron(II) trifluoromethanesulfonate, iron(III) trifluoromethanesulfonate, and (2,2,6,6-tetramethylpiperidin-1-yl)oxyl were obtained from Ambeed. Iron (III) chloride, iron (II) bromide, diiron nanocarbonyl, and triiron dodecarbonyl were purchased from Thermo Scientific. Iron (II) fluoride, iron (III) fluoride, and 4-benzenethiol were purchased from Strem Chemicals. 3-Nitro disulfide, and 4-(trifluoromethyl)thiophenol were purchased from Matrix Scientific. 2-Thienyl disulfide was purchased from Apollo Scientific. Reaction temperatures were controlled using IKA Plates (RCT digital) and the built-in temperature modulators. Thin layer chromatography (TLC) was conducted

with EMD gel 60 F254 pre-coated plates (0.25 mm) and visualized using a combination of UV light, potassium permanganate, phosphomolybdic acid, and p-anisaldehyde staining. Silicycle Silica flash P60 (particle size 0.040–0.063 mm) was used for flash column chromatography. ^{1}H NMR spectra were recorded on a Mercury (400 MHz), or Varian spectrometers (500, 600 MHz) and are reported relative to deuterated solvent signals. Data for ^{1}H NMR spectra are reported as follows: chemical shift (δ ppm), multiplicity, coupling constant (Hz) and integration. ^{13}C NMR spectra were recorded on Mercury (100 MHz), or Varian spectrometers (125 MHz, 150 MHz) and are reported relative to deuterated solvent signals. ^{19}F spectra were recorded on a Varian spectrometer (564 MHz). IR data were collected on a Mettler Toledo ReactIR 702L equipped with a TE MCT detector, an AgX 6mm x 1.5m Fiber probe interface, and a DiComp diamond probe tip. All IR data are reported in terms of frequency absorption (cm^{-1}). Melting points were recorded on a VWR melting point apparatus, high resolution mass (HRMS) spectra were obtained on an Agilent 6545Q-TOF LC/MS, and chiral SFC data were collected on an Agilent 1260 Hybrid SFC/UHPLC system equipped with the following chiral columns: ChiralPak IA-3, 4.6x250mm, 3 mic; ChiralPak IB N-3, 4.6x250mm, 3 mic; ChiralPak IC-3, 4.6x250mm, 3 mic; ChiralPak IH-3, 4.6x250mm, 3 mic; ChiralPak IG-3, 4.6x250mm, 3 mic; ChiralPak IJ-3, 4.6x250mm, 3 mic; and ChiralPak IK-3, 4.6x250mm, 3 mic.

2. General procedure for thioether synthesis from disulfides catalyzed by iron – reaction discovery.

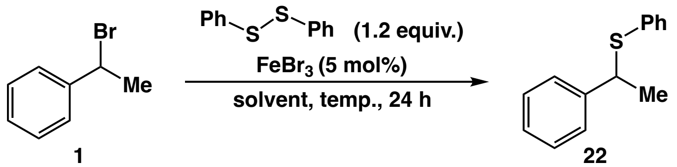

1 22

General scheme for reaction discovery.

To a flame-dried 4 mL dram vial equipped with a stir bar was added $FeBr_3$ (7.4 mg, 0.025 mmol, 5 mol%) in a glove box. The vial was taken out of the glove box and placed under N_2 in a fume-hood. To the vial was added solvent (2.0 mL), (1-bromoethyl)benzene (**1**) (92.5 mg, 0.5 mmol, 68 uL, 1.0 equiv.), and diphenyl disulfide (131 mg, 0.60 mmol, 1.2 equiv.). The reaction vessel was sealed with a teflon screw cap and the mixture was stirred at 40 °C for 24 hours. The mixture was quenched with water (1.0 mL) and the aqueous layer was extracted with diethyl ether (3 x 1.0 mL). The organic layers were combined, dried over anhydrous sodium sulfate, and concentrated under reduced pressure. The resultant mixture was purified by silica flash chromatography (100% hexanes) to yield phenyl thioether **22** as a colorless liquid. (R_f = 0.30, 100% hexanes).

Entry	Solvent	Temperature	Results
1	acetonitrile	40 °C	majority decomposition[1]
2	THF	40 °C	majority decomposition[1]
3	Et_2O	40 °C	majority decomposition[1]
4	1,4–dioxane	40 °C	majority decomposition[1]
5	cyclohexane	40 °C	majority decomposition[1]
6	DMF	40 °C	NR
7	EtOAc	40 °C	majority decomposition[1]
8	acetone	40 °C	4%[2]
9	acetone	55 °C	15%[2]

[1] Starting material was consumed, only trace amounts of product was observed by NMR of the crude mixture. [2] Only product and starting material were present per NMR analysis. Value was calculated using NMR integration and the following equation: [product/(product+starting material)] x 100. Me, methyl group; Ph, phenyl group; THF, tetrahydrofuran; Et, ethyl group; DMF, dimethyl formamide; Ac, acetyl group; NR, no reaction.

Table S1. Reaction discovery.

3. General procedure for thioether synthesis from disulfides catalyzed by iron – reaction optimization.

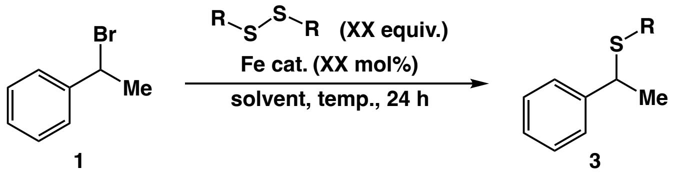

General scheme for reaction optimization.

To a flame-dried 4 mL dram vial equipped with a stir bar was added an iron catalyst (0.025 mmol, 5 mol% or 0.05 mmol, 10 mol%) in a glove box. The vial was taken out of the glove box and placed under N_2 in a fume-hood. To the vial was added solvent (2.0 mL), (1-bromoethyl)benzene (**1**) (92.5 mg, 0.5 mmol, 68 uL, 1.0 equiv.), and disulfide (0.60 mmol, 1.2 equiv. or 0.75 mmol, 1.5 equiv.). The reaction vessel was sealed with a teflon screw cap and the mixture was stirred at the designated temperature for 24 hours. The mixture was quenched with water (1.0 mL) and the aqueous layer was extracted with diethyl ether (3 x 1.0 mL). The organic layers were combined, dried over anhydrous sodium sulfate, and concentrated under reduced pressure. The resultant mixture was purified by silica flash chromatography (100% hexanes) to yield thioethers **3** liquids.

Entry	Disulfide (XX equiv.)	Iron source (XX mol%)	Solvent	Temperature	Results[2]
1	R = Ph (1.2)	$FeBr_3$ (5 mol%)	acetone	40 °C	4%
2	R = Ph (1.2)	$FeBr_3$ (5 mol%)	acetone	55 °C	15%
3	R = Ph (1.2)	$Fe(acac)_3$ (5 mol%)[1]	acetone	55 °C	15%
4	R = Ph (1.2)	$FeCl_2$ (5 mol%)	acetone	55 °C	31%
5	R = Ph (1.2)	$Fe(CO)_5$ (5 mol%)	acetone	55 °C	57%
6	R = Ph (1.2)	$Fe(CO)_5$ (10 mol%)	acetone	55 °C	67%
7	R = Ph (1.2)	no Fe	acetone	55 °C	NR
8	R = Ph (1.2)	36W 450–460 nm blue LED, no iron	acetone	55 °C	NR
9	R = Ph (1.2)	36W 450–460 nm blue LED, $Fe(CO)_5$, (5 mol%)	acetone	55 °C	51%
10	R = Ph (1.5)	$Fe(CO)_5$ (10 mol%)	pinacolone	90 °C	78%
11	R = Ph (1.5)	$Fe(CO)_5$ (10 mol%)	pinacolone	100 °C	83%
12	R = Ph (1.5)	$Fe(CO)_5$ (10 mol%)	pinacolone	107 °C	92%[3]
13	R = Me (1.5)	$Fe(CO)_5$ (10 mol%)	pinacolone	107 °C	98%[3]

[1] $Fe(acac)_3$ is bench stable and was weighed outside of the glove box. [2] Only product and starting material were present per NMR analysis. Value was calculated using NMR integration and the following equation: [product/(product+starting material)] x 100. [3] Isolated yield. Me, methyl group; R, aryl or alkyl group; Ph, phenyl group; acac, acetylacetonate; NR, no reaction.

Table S2. Reaction optimization.

4. General procedure for thioether synthesis from disulfides catalyzed by iron – metal evaluation.

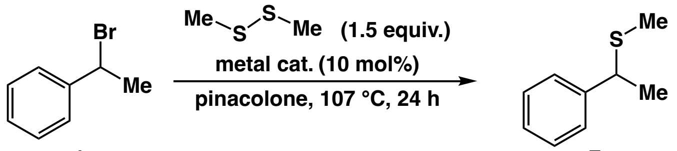

1 7

General scheme for metal evaluation.

To a flame-dried 4 mL dram vial equipped with a stir bar was added a metal catalyst (0.05 mmol, 10 mol%) in a glove box. The vial was taken out of the glove box and placed under N_2 in a fume-hood. To the vial was added pinacolone (1.0 mL), (1-bromoethyl)benzene (**1**) (92.5 mg, 0.5 mmol, 68 uL, 1.0 equiv.), and dimethyl disulfide (70.7 mg, 0.75 mmol, 67.5 uL, 1.5 equiv.). The reaction vessel was sealed with a teflon screw cap and the mixture was stirred at 107 °C for 24 hours. The mixture was quenched with water (1.0 mL) and the aqueous layer was extracted with diethyl ether (3 x 1.0 mL). The organic layers were combined, dried over anhydrous sodium sulfate, and concentrated under reduced pressure. The resultant mixture was purified by silica flash chromatography (100% hexanes) to yield methyl thioether **7** as a colorless liquid. (R_f = 0.30, 100% hexanes).

Entry	Metal cat.	Isolated Yield %
1	$MgCl_2$	5
2	$AlCl_3$	5
3	$Ni(cod)_2$	NR
4	$Ni(PPh_3)_4$	33
5	$CuBr_2$	33
6	$Pd(dba)_3$	30
7	$Pd(PPh_3)_4$	21
8	$PdCl_2$	21
9	$Fe(OTf)_2$	43
10	$Fe(OTf)_3$	48
11	FeF_2	38
12	FeF_3	49
13	$FeCl_2$	57
14	$FeCl_3$	51
15	$FeCl_2.4H_2O$	51
16	$FeBr_2$	49
17	$FeBr_3$	45
18	$Fe_2(CO)_9$	55
19	$Fe_3(CO)_{12}$	49
20	$Fe(CO)_5$	77
21	$Fe(CO)_5$ (5 mol%)[1]	66
22	$Fe(CO)_5$, 12 h	75[2]
23	$Fe(CO)_5$	98[3]
24	no catalyst	NR

[1] This reaction was run with 5 mol% of metal catalyst. [2] The reaction was run for 12 h. [3] The reaction was run for 12 h with 5 mol% of $Fe(CO)_5$, then an addtional 5 mol% of $Fe(CO)_5$ was added, and the reaction was stirred for an addtional 12 h. Me, methyl group; cod, cyclooctadiene; Ph, phenyl group; dba, dibenzylideneacetone; Tf, triflate; NR, no reaction

Table S3. Metal evaluation.

5. General procedure for thioether synthesis from disulfides catalyzed by iron – additive evaluation.

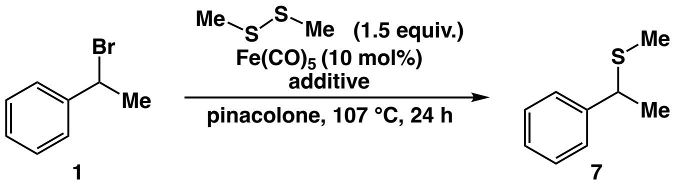

General scheme for additive evaluation.

To a flame-dried 4 mL dram vial equipped with a stir bar was added a $Fe(CO)_5$ (9.9 mg, 0.05 mmol, 6.8 uL, 10 mol%) in a glove box. The vial was taken out of the glove box and placed under N_2 in a fume-hood. To the vial was added pinacolone (1.0 mL), the additive, (1-bromoethyl)benzene (**1**) (92.5 mg, 0.5 mmol, 68 uL, 1.0 equiv.), and dimethyl disulfide (70.7 mg, 0.75 mmol, 67.5 uL, 1.5 equiv.). The reaction vessel was sealed with a teflon screw cap and the mixture was stirred at 107 °C for 24 hours. The mixture was quenched with water (1.0 mL) and the aqueous layer was extracted with diethyl ether (3 x 1.0 mL). The organic layers were combined, dried over anhydrous sodium sulfate, and concentrated under reduced pressure. The resultant mixture was purified by silica flash chromatography (100% hexanes) to yield methyl thioether **7** as a colorless liquid. (R_f = 0.30, 100% hexanes).

Entry	Additive (XX equiv.)	Isolated Yield %[1]
1	1.0 uL H_2O (10 mol%)	46
2	Cs_2CO_3 (1.2 equiv)	7
3	K_2CO_3 (1.2 equiv)	34
4	L1 (10 mol%)	55
5	L2 (10 mol%)	52
6	L3 (10 mol%)	33
7	L4 (10 mol%)	55

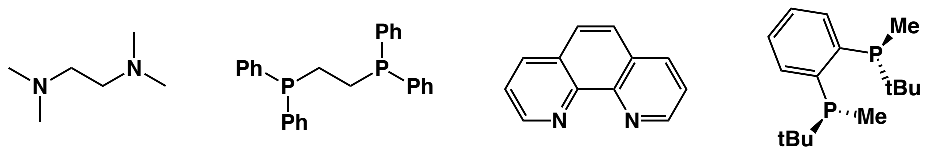

L1 TMEDA **L2** DIPHOS **L3** 1,10-phenanthroline **L4** (*R*)-BenzP

[1] The reaction was run using the sequential addition protocol (Table S3, entry 23). Me, methyl group; TMEDA, tetramethylethylenediamine; DIPHOS, 1,2-bis(diphenylphosphino)ethane; (*R*)-BenzP, 1,2-Bis((R)-tert-butyl(methyl)phosphino)benzene

Table S4. Additive evaluation.

6. Failed substrates in thioether synthesis from disulfides catalyzed by iron.

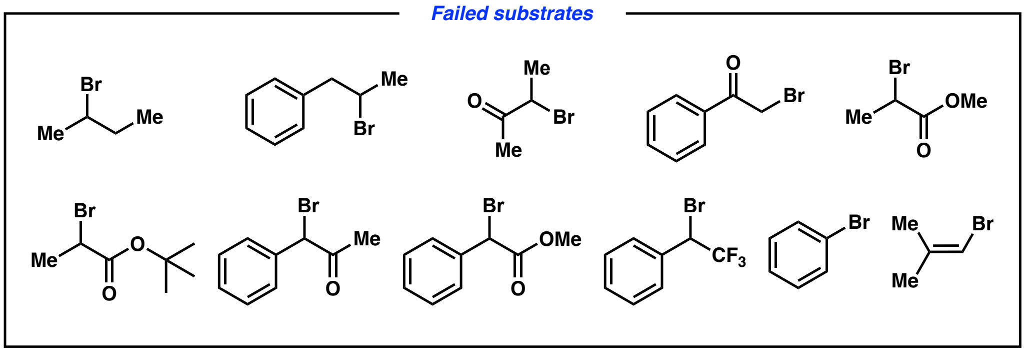

Table S5. Failed bromide substrate classes.

7. General optimized procedure for thioether synthesis from disulfides catalyzed by iron.

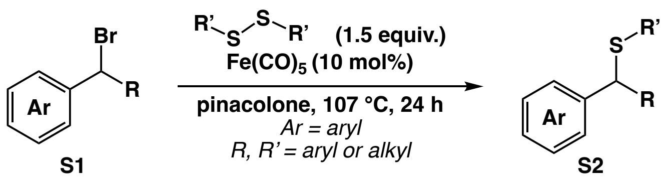

General scheme for optimized procedure.

To a flame-dried 4 mL dram vial equipped with a stir bar was added $Fe(CO)_5$ (5.0 mg, 0.025 mmol, 3.4 uL, 5 mol%) in a glove box. The vial was taken out of the glove box and placed under N_2 in a fume-hood. To the vial was added pinacolone (1.0 mL), bromide coupling partner (**S1**) (0.5 mmol, 1.0 equiv.), and disulfide (0.75 mmol, 1.5 equiv.). The reaction vessel was sealed with a teflon screw cap and the mixture was stirred at 107 °C. The reaction mixture was cooled to room temperature after 12 hours, and an additional portion of $Fe(CO)_5$ (5.0 mg, 0.025 mmol, 3.4 uL, 5 mol%) from the glove box was added via syringe. The reaction was stirred for an additional 12 h at 107 °C. The mixture was quenched with water (1.0 mL) and the aqueous layer was extracted with diethyl ether (3 x 1.0 mL). The organic layers were combined, dried over anhydrous sodium sulfate, and concentrated under reduced pressure. The resultant mixture was purified by silica flash chromatography to yield thioethers **S2**.

8. Synthesis of disulfides.

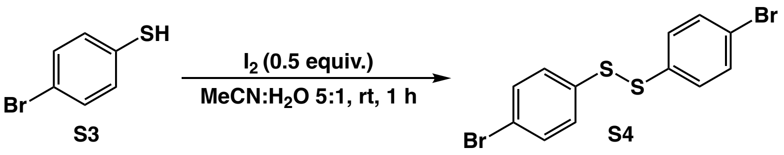

Synthesis of disulfide **S4**.

1,2-Bis(4-bromophenyl)disulfide (S4). To a flame-dried 20 mL vial equipped with a stir bar was added 4-bromobenzenethiol (**S3**) (567.2 mg, 3.0 mmol, 1.0 equiv.), MeCN (10.0 mL), and deionized water (2.0 mL). Iodine (380.7 mg, 1.5 mmol, 0.5 equiv.) was added in one portion and the mixture was stirred at room temperature for 1 h. The reaction was quenched with water (5.0 mL) and extracted with EtOAc (3 x 3.0 mL). The organic layers were combined, dried over Na_2SO_4, and concentrated under reduced pressure. The resultant residue was purified by silica flash chromatography (1:5 EtOAc:hexane) to yield disulfide **S4** as a white solid (532.6 mg, 95% yield). **^{1}H NMR (400 MHz, $CDCl_3$).** δ = 7.43 (dd, J = 8.5, 1.7 Hz, 4H), 7.33 (dd, J = 8.6, 1.7 Hz, 4H). Spectral data matched those reported previously.[1]

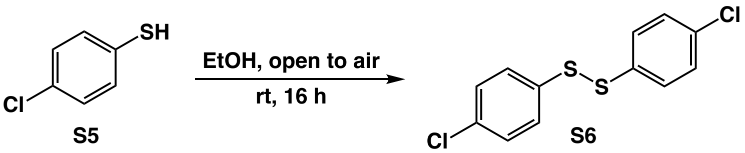

Synthesis of disulfide **S6**.

1,2-Bis(4-chlorophenyl)disulfide (S6). To a flame-dried 20 mL vial equipped with a stir bar was added 4-chlorobenzenethiol (**S5**) (1.446 g, 10.0 mmol, 1.0 equiv.) and EtOH (10.0 mL). The mixture was stirred at room temperature, open to air, for 16 hours. The reaction was concentrated under reduced pressure and the resultant residue was purified by silica flash chromatography (100% hexanes) to yield disulfide **S6** as a white solid (1.344 g, 94% yield). **^{1}H NMR (400 MHz, $CDCl_3$).** δ = 7.42–7.36 (m, 4H), 7.30–7.23 (m, 4H). Spectral data matched those reported previously.[2]

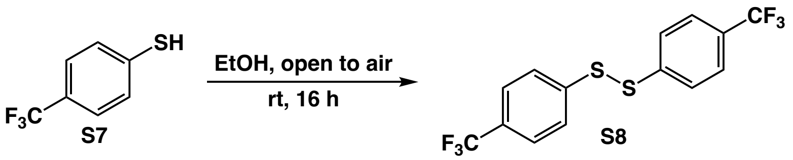

Synthesis of disulfide **S8**.

1,2-Bis(4-(trifluoromethyl)phenyl)disulfide (S8). To a flame-dried 20 mL vial equipped with a stir bar was added 4-(trifluoromethyl)benzenethiol (**S7**) (356.7 mg, 2.0 mmol, 1.0 equiv.) and EtOH (10.0 mL). The mixture was stirred at room temperature, open to air, for 16 hours. The reaction was concentrated under reduced pressure and the resultant oil was purified by silica flash chromatography (100% hexanes) to yield disulfide **S8** as a colorless oil (294.9 mg, 81% yield). **^{1}H NMR (400 MHz, $CDCl_3$).** δ = 7.61–7.55 (m, 8H). Spectral data matched those reported previously.[3]

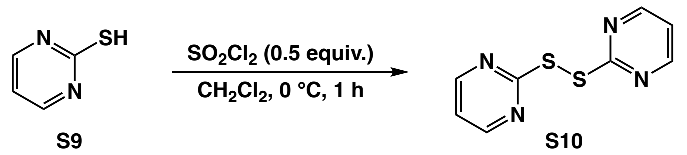

Synthesis of disulfide **S10**.

1,2-di(pyrimidin-2-yl)disulfane (S10). To a flame-dried 20 mL vial equipped with a stir bar was added pyrimidine-2-thiol (**S9**) (1.40 g, 12.4 mmol, 1.0 equiv.) and CH_2Cl_2 (10.0 mL). The solution was cooled to 0 °C and sulfuryl chloride (0.84 g, 6.2 mmol, 0.5 equiv.) was added dropwise. The mixture was stirred at 0 °C for 1 hour, then concentrated under reduced pressure to yield disulfide **S10** as a light yellow solid (1.87 g, 68% yield). **^{1}H NMR (500 MHz, DMSO-*d*$_6$).** δ = 8.69 (d, *J* = 4.9 Hz, 4H), 7.36 (t, *J* = 4.8 Hz, 2H). Spectral data matched those reported previously.[4]

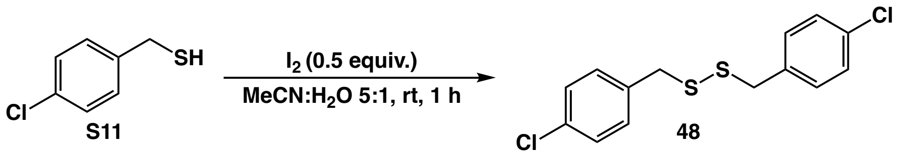

Synthesis of disulfide **48**.

1,2-Bis(4-chlorobenzyl)disulfide (48). To a flame-dried 20 mL vial equipped with a stir bar was added (4-chlorophenyl)methanethiol (**S11**) (476 mg, 3.0 mmol, 1.0 equiv.), MeCN (10.0 mL), and deionized water (2.0 mL). Iodine (380.7 mg, 1.5 mmol, 0.5 equiv.) was added in one portion and the mixture was stirred at room temperature for 1 h. The reaction was quenched with water (5.0 mL) and extracted with EtOAc (3.0 mL x 3). The organic layers were combined, dried over Na_2SO_4, and concentrated under reduced pressure. The resultant oil was purified by silica flash chromatography (1:5 EtOAc:hexane) to yield disulfide **48** as a colorless oil (284 mg, 60% yield). **^{1}H NMR (400 MHz, $CDCl_3$).** δ = 7.29 (dd, J = 8.4, 2.1 Hz, 4H), 7.15 (dd, J = 8.5, 2.2 Hz, 4H), 3.58 (d, J = 2.2 Hz, 4H). Spectral data matched those reported previously.[5]

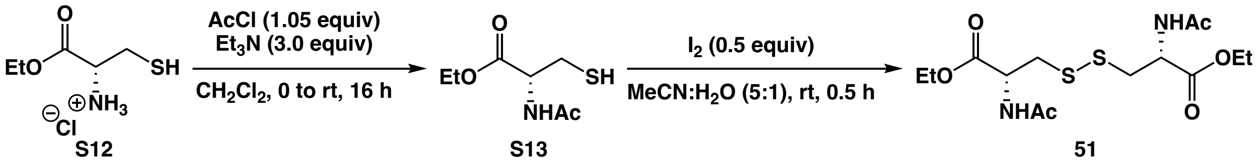

Synthesis of disulfide **51**.

Diethyl 3,3'-disulfanediyl(2R,2'R)-bis(2-acetamidopropanoate) (51). To a flame-dried 20 mL vial equipped with a stir bar was added L-Cysteine ethyl ester hydrochloride (**S12**) (500 mg, 2.69 mmol, 1.0 equiv.), Et_3N (1.1 mL, 8.07 mmol, 3.0 equiv.), and CH_2Cl_2 (15.0 mL). The mixture was cooled to 0 °C and stirred for 10 minutes before adding acetyl chloride (201 uL, 2.83 mmol, 1.05 equiv.) dropwise over 5 minutes. The reaction was allowed to warm to room temperature and then stirred for an additional 16 h. The reaction was quenched with water (10.0 mL), the aqueous layer was extracted with CH_2Cl_2 (5.0 mL x 3), and the organic layers were combined, dried over Na_2SO_4, and concentrated under reduced pressure. The resultant mixture was purified by silica flash chromatography (100% EtOAc) to yield thiol **S13** as a colorless liquid (514 mg, quant. yield).

The resultant thiol **S13** was added to a flame-dried 20 mL vial equipped with a stir bar, and MeCN (10.0 mL) and deionized water (2.0 mL) were added. Iodine (319 mg, 1.26 mmol, 0.5 equiv.) was added in one portion and the mixture was stirred at room temperature for 0.5 h. The reaction was quenched with water (5.0 mL) and extracted with EtOAc (3.0 mL x 3). The organic layers were combined, dried over Na_2SO_4, and concentrated under reduced pressure. The resultant mixture was purified by silica flash chromatography (100% EtOAc) to yield disulfide **51** as a deep orange colored solid (433 mg, 42% yield). (R_{f} = 0.31, 100% EtOAc). **Mp:** 110 – 113 °C. **^{1}H NMR (600 MHz, $CDCl_3$).** δ = 6.55 (d, *J* = 7.4 Hz, 2H), 4.84 (dt, *J* = 7.6, 2.6 Hz, 2H), 4.23 (q, *J* = 6.5 Hz, 4H), 3.22 (d, *J* = 3.6 Hz, 4H), 2. 07 (s, 6H), 1.29 (t, *J* = 7.1 Hz, 6H). **^{13}C NMR (150 MHz, $CDCl_3$).** δ = 170.6, 170.5, 62.4, 52.3, 41.2, 23.5, 14.3. **IR (solution, MeCN).** 2928, 2840, 2580, 1744, 1680, 1524 cm^{-1}. **HRMS-QTOF-ESI** (m/z) $[M + Ag]^+$ calcd for $C_{14}H_{24}AgN_2O_6S_2^+$, 487.0122; found 487.0119.

9. Synthesis of bromides and other (pseudo)halides.

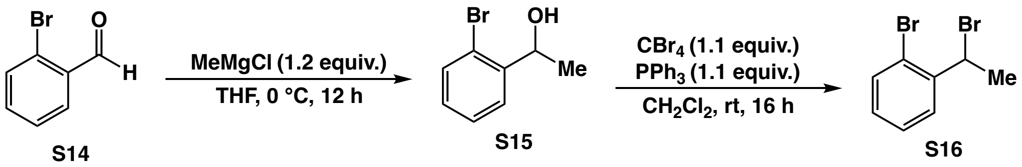

Synthesis of bromide **S16**.

1-Bromo-2-(1-bromoethyl)benzene (S16). To a flame-dried 50 mL round-bottom-flask equipped with a stir bar was added 2-bromobenzaldehyde (**S14**) (463 mg, 2.5 mmol, 1.0 equiv.) and THF (6.0 mL). The mixture was cooled to 0 °C under a positive pressure of N_2 and MeMgCl (3.0 M in THF, 1.0 mL, 3.0 mmol, 1.2 equiv.) was then added dropwise over 30 min. The mixture was then warmed to room temperature and stirred for an additional 12 h. The reaction was quenched with saturated NH_4Cl (10.0 mL) and extracted with EtOAc (5.0 mL x 3). The organic layers were combined, dried over Na_2SO_4, and concentrated under reduced pressure. The resultant oil was purified by silica flash chromatography (1:5 EtOAc:hexane) to yield alcohol **S15** as a pale yellow oil (400 mg, 74% yield).

The resultant alcohol **S15** was added to a flame-dried 50 mL round-bottom-flask equipped with a stir bar. CH_2Cl_2 (6.0 mL) and PPh_3 (580 mg, 2.2 mmol, 1.1 equiv.) were then added to the solution. CBr_4 (0.74 g, 2.2 mmol, 1.1 equiv.) was dissolved in CH_2Cl_2 (3.0 mL) in a separate vial and then added to the reaction mixture dropwise over 30 min at room temperature. The reaction was stirred for 16 h, quenched with water (5.0 mL), and extracted with CH_2Cl_2 (3.0 mL x 3). The organic layers were combined, dried over Na_2SO_4, and concentrated under reduced pressure. The resultant oil was purified by silica flash chromatography (1:5 EtOAc:hexane) to yield bromide **S16** as a light yellow oil (254 mg, 48% yield). **^{1}H NMR (400 MHz, $CDCl_3$)** δ = 7.65 (dd, *J* = 8.0, 1.7 Hz, 1H), 7.54 (dd, *J* = 8.0, 1.3 Hz, 1H), 7.35 (td, *J* = 7.6, 1.3 Hz, 1H), 7.16 – 7.12 (m, 1H), 5.61 (q, *J* = 6.9 Hz, 1H), 2.04 (d, *J* = 6.9 Hz, 3H). Spectral data matched those reported previously.[6]

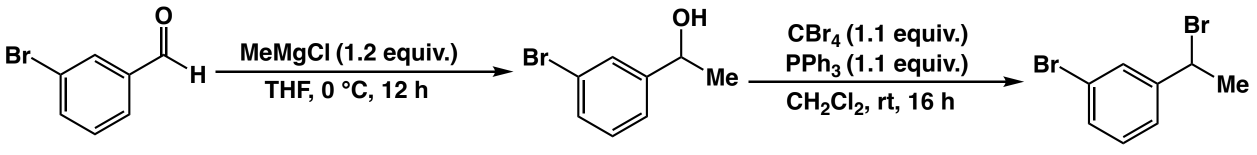

S17 **S18** **S19**

Synthesis of bromide **S19**.

1-Bromo-3-(1-bromoethyl)benzene (S19). To a flame-dried 50 mL round-bottom-flask equipped with a stir bar was added 3-bromobenzaldehyde (**S17**) (0.463 g, 2.5 mmol, 1.0 equiv.) and THF (6.0 mL). The mixture was cooled to 0 °C under a positive pressure of N_2 and MeMgCl (3.0 M in THF, 1.0 mL, 3.0 mmol, 1.2 equiv.) was then added dropwise over 30 min. The mixture was then warmed to room temperature and stirred for an additional 12 h. The reaction was quenched with saturated NH_4Cl (10.0 mL) and extracted with EtOAc (5.0 mL x 3). The organic layers were combined, dried over Na_2SO_4, and concentrated under reduced pressure. The resultant oil was purified by silica flash chromatography (1:5 EtOAc:hexane) to yield alcohol **S18** as a pale yellow oil (424 mg, 78% yield).

The resultant alcohol **S18** was added to a flame-dried 50 mL round-bottom-flask equipped with a stir bar. CH_2Cl_2 (6.0 mL) and PPh_3 (610 mg, 2.3 mmol, 1.1 equiv.) were then added to the solution. CBr_4 (0.78 g, 2.3 mmol, 1.1 equiv.) was dissolved in CH_2Cl_2 (3.0 mL) in a separate vial and then added to the reaction mixture dropwise over 30 min at room temperature. The reaction was stirred for 16 h, quenched with water (5.0 mL), and extracted with CH_2Cl_2 (3.0 mL x 3). The organic layers were combined, dried over Na_2SO_4, and concentrated under reduced pressure. The resultant oil was purified by silica flash chromatography (1:5 EtOAc:hexane) to yield bromide **S19** as a light yellow oil (372 mg, 67% yield). **^{1}H NMR (400 MHz, $CDCl_3$).** δ = 7.58 (s, 1H), 7.44–7.39 (m, 1H), 7.36 (d, J = 7.7 Hz, 1H), 7.21 (t, J = 7.9 Hz, 1H), 5.12 (q, J = 6.9 Hz, 1H), 2.02 (d, J = 6.9 Hz, 3H). Spectral data matched those reported previously.[7]

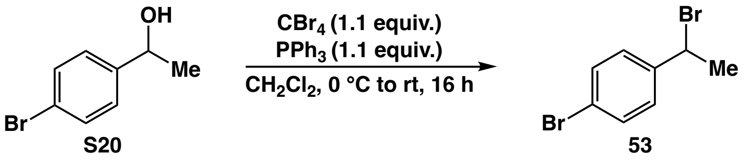

Synthesis of bromide **53**.

1-Bromo-4-(1-bromoethyl)benzene (53). To a flame-dried 20 mL vial equipped with a stir bar was added 1-(4-bromophenyl)ethan-1-ol (**S20**) (500 mg, 2.49 mmol, 1.0 equiv.) and CH_2Cl_2 (8.0 mL). The reaction mixture was cooled to 0 °C under a positive pressure of N_2 and PPh_3 (720 mg, 2.74 mmol, 1.1 equiv.) was then added to the solution. CBr_4 (0.91 g, 2.74 mmol, 1.1 equiv.) was dissolved in CH_2Cl_2 (4.0 mL) in a separate vial and then added to the reaction mixture dropwise over 30 min at room temperature. The reaction was stirred for 16 h, quenched with water (5.0 mL), and extracted with CH_2Cl_2 (3.0 mL x 3). The organic layers were combined, dried over Na_2SO_4, and concentrated under reduced pressure. The resultant oil was purified by silica flash chromatography (100% hexanes) to yield bromide **53** as a light yellow oil (275 mg, 53% yield). **^{1}H NMR (400 MHz, $CDCl_3$).** δ = 7.47 (d, *J* = 6. 7 Hz, 2H), 7.31 (d, *J* = 7.1 Hz, 2H), 5.15 (q, *J* = 7.3 Hz, 1H), 2.02 (d, *J* = 7.2 Hz, 3H). Spectral data matched those reported previously.[8]

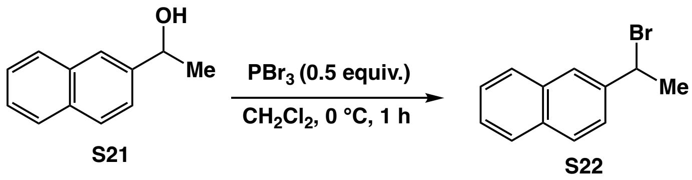

Synthesis of bromide **S22**.

2-(1-Bromoethyl)naphthalene (S22). To a flame-dried 20 mL vial equipped with a stir bar was added 1-(naphthalen-2-yl)ethan-1-ol (**S21**) (172.22 mg, 1.0 mmol, 1.0 equiv.) and CH_2Cl_2 (3.0 mL). The reaction mixture was cooled to 0 °C under a positive pressure of N_2 and PBr_3 (47.0 uL, 0.5 mmol, 0.5 equiv.) was added to the solution dropwise. The reaction was then stirred for 1 hour at 0 °C. The reaction was quenched with water (5.0 mL) and extracted with CH_2Cl_2 (3.0 mL x 3). The organic layers were combined, dried over Na_2SO_4, and concentrated under reduced pressure. Bromide **S22** was unstable to silica flash chromatography purification conditions and was used without further purification. **^{1}H NMR (400 MHz, $CDCl_3$).** δ = 7.86–7.80 (m, 4H), 7.59 (d, *J* = 8.7

Hz, 1H), 7.52–7.46 (m, 2H), 5.40 (q, J = 6.3 Hz, 1H), 2.15 (d, J = 6.9 Hz, 3H). Spectral data matched those reported previously.[6]

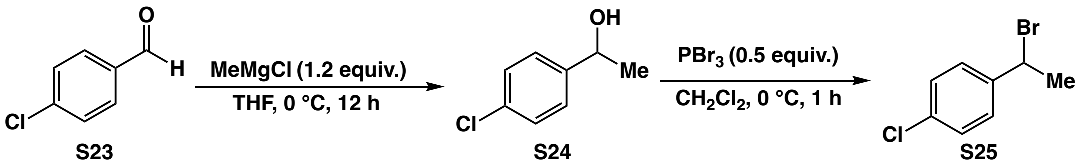

Synthesis of bromide **S25**.

1-(1-Bromoethyl)-4-chlorobenzene (S25). To a flame-dried 50 mL round-bottom-flask equipped with a stir bar was added 4-chlorobenzaldehyde (**S23**) (500 mg, 3.6 mmol, 1.0 equiv.) and THF (12.0 mL). The mixture was cooled to 0 °C under a positive pressure of N_2 and MeMgCl (3.0 M in THF, 1.0 mL, 3.0 mmol, 1.2 equiv.) was then added dropwise over 30 min. The mixture was then warmed to room temperature and stirred for an additional 12 h. The reaction was quenched with saturated NH_4Cl (10.0 mL) and extracted with EtOAc (5.0 mL x 3). The organic layers were combined, dried over Na_2SO_4, and concentrated under reduced pressure. The resultant oil was purified by silica flash chromatography (9:1 to 7:3 hexanes:EtOAc) to yield alcohol **S24** as a pale yellow oil (357 mg, 63% yield).

The resultant alcohol **S24** was added to a flame-dried 20 mL vial equipped with a stir bar. CH_2Cl_2 (11.0 mL) was added and the solution was cooled to 0 °C. PBr_3 (104 uL, 1.1 mmol, 0.5 equiv.) was added to the solution dropwise, and reaction mixture was further stirred for 1 h at 0 °C. The reaction was quenched with water (5.0 mL) and extracted with CH_2Cl_2 (3.0 mL x 3). The organic layers were combined, dried over Na_2SO_4, and concentrated under reduced pressure. The resultant oil was purified by silica flash chromatography (100% hexanes) to yield bromide **S25** as a colorless oil (215 mg, 43% yield). **^{1}H NMR (600 MHz, $CDCl_3$).** δ 7.37 (d, J = 8.5 Hz, 2H), 7.32 (d, J = 8.5 Hz, 2H), 5.18 (q, J = 7.1 Hz, 1H), 2.03 (d, J = 7.1 Hz, 3H). Spectral data matched those reported previously.[7]

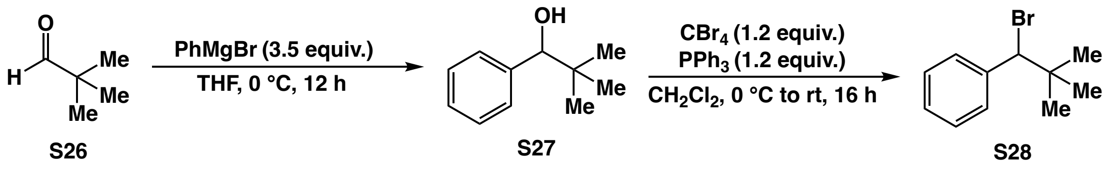

Synthesis of bromide **S28**.

(1-Bromo-2,2-dimethylpropyl)benzene (S28). To a flame-dried 50 mL round-bottom-flask equipped with a stir bar was added pivalaldehyde (**S26**) (500 mg, 5.8 mmol, 1.0 equiv.) and THF (11.6 mL). The mixture was cooled to 0 °C under a positive pressure of N_2 and PhMgBr (3.0 M in THF, 6.8 mL, 20.3 mmol, 3.5 equiv.) was then added dropwise over 30 min. The mixture was then warmed to room temperature and stirred for an additional 12 h. The reaction was quenched with saturated NH_4Cl (10.0 mL) and extracted with EtOAc (5.0 mL x 3). The organic layers were combined, dried over Na_2SO_4, and concentrated under reduced pressure. The resultant oil was purified by silica flash chromatography to yield alcohol **S27** as a pale yellow oil (953 mg, quant. yield).

The resultant alcohol **S27** was added to a flame-dried 20 mL vial equipped with a stir bar. CH_2Cl_2 (58.0 mL) was added and the solution was cooled to 0 °C under a positive pressure of N_2. PPh_3 (1.825 g, 6.96 mmol, 1.2 equiv.) and CBr_4 (2.307 g, 6.96 mmol, 1.2 equiv.) were then added to the solution. The reaction was stirred for 16 h, quenched with water (5.0 mL), and extracted with CH_2Cl_2 (3.0 mL x 3). The organic layers were combined, dried over Na_2SO_4, and concentrated under reduced pressure. The resultant oil was purified by silica flash chromatography (8:2 hexanes:EtOAc) to yield bromide **S28** as a light yellow oil (1.07 g, 81% yield). **^{1}H NMR (400 MHz, $CDCl_3$).** δ = 7.37–7.23 (m, 5H), 5.26 (s, 1H), 1.08 (s, 9H), Spectral data matched those reported previously.[6]

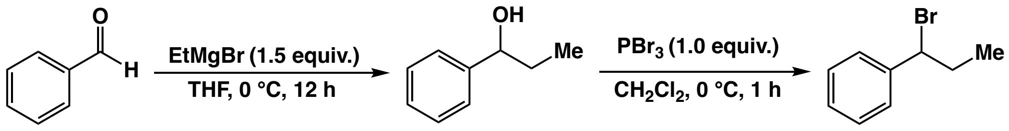

S29 S30 S31

Synthesis of bromide **S31**.

(1-Bromopropyl)benzene (S31). To a flame-dried 50 mL round-bottom-flask equipped with a stir bar was added benzaldehyde (**S29**) (500 mg, 4.7 mmol, 1.0 equiv.) and THF (12.0 mL). The mixture was cooled to 0 °C under a positive pressure of N_2 and EtMgBr (3.0 M in diethyl ether, 7.1 mL, 7.06 mmol, 1.5 equiv.) was then added dropwise over 30 min. The mixture was then warmed to room temperature and stirred for an additional 12 h. The reaction was quenched with saturated NH_4Cl (10.0 mL) and extracted with EtOAc (5.0 mL x 3). The organic layers were combined, dried over Na_2SO_4, and concentrated under reduced pressure. The resultant oil was purified by silica flash chromatography (8:2 hexanes:EtOAc) to yield alcohol **S30** as a pale yellow oil and used directly in the next step.

The resultant alcohol **S30** was added to a flame-dried 20 mL vial equipped with a stir bar. CH_2Cl_2 (12.0 mL) was added and the solution was cooled to 0 °C. PBr_3 (174 uL, 4.7 mmol, 1.0 equiv.) was added to the solution dropwise, and reaction mixture was further stirred for 1 h at 0 °C. The reaction was quenched with water (5.0 mL) and extracted with CH_2Cl_2 (3.0 mL x 3). The organic layers were combined, dried over Na_2SO_4, and concentrated under reduced pressure. The resultant oil was purified by silica flash chromatography (100% hexanes) to yield bromide **S31** as a colorless oil (511 mg, 55% yield, 2 steps). **^{1}H NMR (400 MHz, $CDCl_3$).** δ = 7.41–7.37 (m, 2H), 7.36–7.31 (m, 2H), 7.30–7.27 (m, 1H), 4.88 (t, *J* = 7.4 Hz, 1H), 2.36–2.32 (m, 1H), 2.22–2.10 (m, 1H), 1.00 (t, *J* = 7.2 Hz, 3H), Spectral data matched those reported previously.[6]

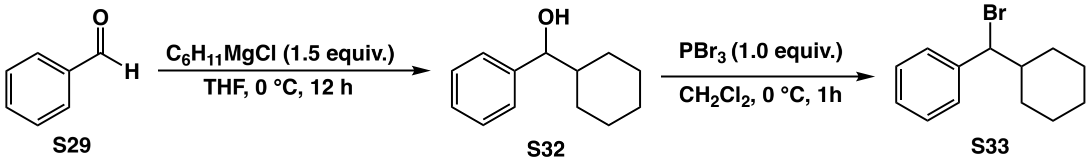

Synthesis of bromide **S33**.

(Bromo(cyclohexyl)methyl)benzene (S33). To a flame-dried 50 mL round-bottom-flask equipped with a stir bar was added benzaldehyde (**S29**) (500 mg, 4.7 mmol, 1.0 equiv.) and THF (12.0 mL). The mixture was cooled to 0 °C under a positive pressure of N_2 and $C_6H_{11}MgCl$ (1.0 M in THF, 7.06 mL, 7.06 mmol, 1.5 equiv.) was then added dropwise over 30 min. The mixture was then warmed to room temperature and stirred for an additional 12 h. The reaction was quenched with saturated NH_4Cl (10.0 mL) and extracted with EtOAc (5.0 mL x 3). The organic layers were combined, dried over Na_2SO_4, and concentrated under reduced pressure. The resultant oil was purified by silica flash chromatography (8:2 hexanes:EtOAc) to yield alcohol **S32** as a pale yellow oil and used directly in the next step.

The resultant alcohol **S32** was added to a flame-dried 20 mL vial equipped with a stir bar. CH_2Cl_2 (12.0 mL) was added and the solution was cooled to 0 °C. PBr_3 (174 uL, 4.7 mmol, 1.0 equiv.) was added to the solution dropwise, and reaction mixture was further stirred for 1 h at 0 °C. The reaction was quenched with water (5.0 mL) and extracted with CH_2Cl_2 (3.0 mL x 3). The organic layers were combined, dried over Na_2SO_4, and concentrated under reduced pressure. The resultant oil was purified by silica flash chromatography (100% hexanes) to yield bromide **S33** as a colorless oil (396 mg, 33% yield, 2 steps). **^{1}H NMR (400 MHz, $CDCl_3$).** δ = 7.44–7.27 (m, 5H), 4.71 (d, *J* = 9.2 Hz, 1H), 2.35–2.25 (m, 1H), 2.02–1.90 (m, 1H), 1.84–1.76 (m, 1H), 1.70–1.57 (m, 2H), 1.51–1.43 (m, 1H), 1.32–0.97 (m, 4H), 0.93–0.78 (m, 1H), Spectral data matched those reported previously.[6]

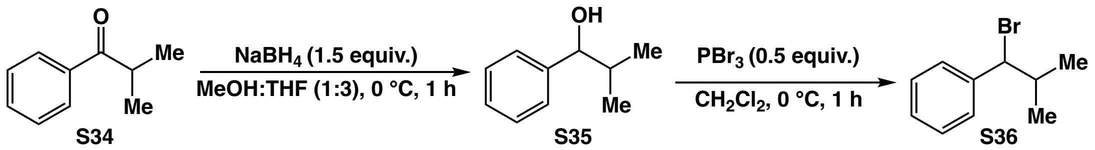

Synthesis of bromide **S36**.

(1-Bromo-2-methylpropyl)benzene (S36). To a flame-dried 20 mL vial equipped with a stir bar was added 2-methyl-1-phenylpropan-1-one (**S34**) (444.6 mg, 3.0 mmol, 1.0 equiv.) along with THF (6.0 mL) and MeOH (2.0 mL). The mixture was cooled to 0 °C under a positive pressure of N_2 and $NaBH_4$ (170.2 mg, 4.5 mmol. 1.5 equiv.) was added. The mixture was stirred for 1 h at 0 °C. The reaction was quenched with water (5.0 mL) and extracted with EtOAc (3.0 mL x 3). The organic layers were combined, dried over Na_2SO_4, and concentrated under reduced pressure. The resultant mixture was purified by silica flash chromatography (1:4 EtOAc:hexane) to yield alcohol **S35** as a pale yellow oil (357 mg, 80.0% yield).

The resultant alcohol **S35** was added to a flame-dried 20 mL vial equipped with a stir bar. CH_2Cl_2 (6.0 mL) was added and the solution was cooled to 0 °C. PBr_3 (120 uL, 1.2 mmol, 0.5 equiv.) was added to the solution dropwise, and reaction mixture was further stirred for 1 h at 0 °C. The reaction was quenched with water (5.0 mL) and extracted with CH_2Cl_2 (3.0 mL x 3). The organic layers were combined, dried over Na_2SO_4, and concentrated under reduced pressure. The resultant oil was purified by silica flash chromatography (1:9 to 1:5 EtOAc:hexane) to yield bromide **S36** as a colorless oil (267 mg, 49% yield). **^{1}H NMR (400 MHz, $CDCl_3$).** δ = 7.39–7.23 (m, 5H), 4.72 (d, *J* = 8.5 Hz, 1H), 2.32 (oct, 6.5 Hz, 1H), 1.19 (d, *J* = 6.6 Hz, 3H), 0.86 (d, *J* = 6.7 Hz, 3H). Spectral data matched those reported previously.[6]

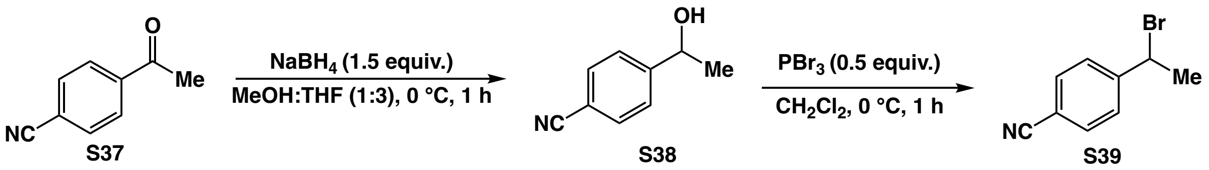

Synthesis of bromide **S39**.

4-(1-Bromoethyl)benzonitrile (S39). To a flame-dried 20 mL vial equipped with a stir bar was added 4-acetylbenzonitrile (**S37**) (436 mg, 3.0 mmol, 1.0 equiv.) along with THF (6.0 mL) and MeOH (2.0 mL). The mixture was cooled to 0 °C under a positive pressure of N_2 and $NaBH_4$ (170.2 mg, 4.5 mmol. 1.5 equiv.) was added. The mixture was stirred for 1 h at 0 °C. The reaction was quenched with water (5.0 mL) and extracted with EtOAc (3.0 mL x 3). The organic layers were combined, dried over Na_2SO_4, and concentrated under reduced pressure. The resultant mixture was purified by silica flash chromatography (1:5 to 1:1 EtOAc:hexane) to yield alcohol **S38** as a pale yellow oil (410 mg, 94% yield).

The resultant alcohol **S38** was added to a flame-dried 20 mL vial equipped with a stir bar. CH_2Cl_2 (6.0 mL) was added and the solution was cooled to 0 °C. PBr_3 (130 uL, 1.36 mmol, 0.5 equiv.) was added to the solution dropwise, and reaction mixture was further stirred for 1 h at 0 °C. The reaction was quenched with water (5.0 mL) and extracted with CH_2Cl_2 (3.0 mL x 3). The organic layers were combined, dried over Na_2SO_4, and concentrated under reduced pressure. The resultant oil was purified by silica flash chromatography (1:5 EtOAc:hexane) to yield bromide **S39** as a colorless oil (311 mg, 55% yield). **^{1}H NMR (500 MHz, $CDCl_3$).** δ = 7.68–7.51 (m, 4H), 5.16 (q, *J* = 6.9, 1H), 2.03 (d, *J* = 6.9 Hz, 3H). Spectral data matched those reported previously.[8]

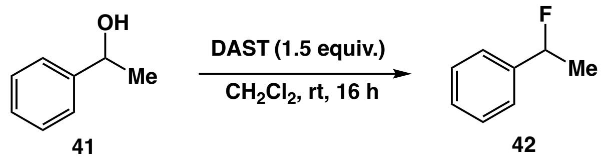

Synthesis of fluoride **42**.

(1-Fluoroethyl)benzene (42). To a flame-dried 20 mL vial equipped with a stir bar was added 1-phenylethan-1-ol (**41**) (500 mg, 4.09 mmol, 1.0 equiv.) and CH_2Cl_2 (8.0 mL). The reaction mixture was cooled to 0 °C under a positive pressure of nitrogen. Diethylaminosulfur trifluoride (DAST) (989 mg, 6.13 mmol, 1.5 equiv.) was then added dropwise, and the reaction was warmed to room temperature and stirred for 16 h. The reaction was quenched with saturated $NaHCO_3$ (5 mL) and extracted with CH_2Cl_2 (5.0 mL x 3). The organic layers were combined, dried over Na_2SO_4, and concentrated under reduced pressure. The resultant mixture was purified with silica flash chromatography (1:11 EtOAc:hexane) to yield fluoride **42** as a light yellow oil (157.4 mg, 31.0% yield). **^{1}H NMR (400 MHz, CDCl$_3$).** δ = 7.42–7.27 (m, 5H), 5.63 (dq, *J* = 47.8, 6.54 Hz, 1H), 1.70–1.59 (m, 3H). Spectral data matched those reported previously.[9]

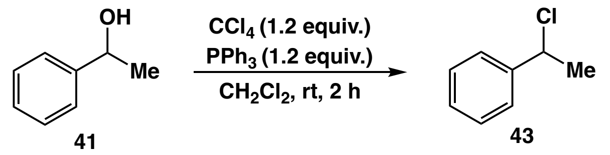

Synthesis of chloride **43**.

(1-Chloroethyl)benzene (43). To a flame-dried 50 mL round-bottom-flask equipped with a stir bar was added triphenylphosphine (1.92 g, 7.3 mmol, 1.2 equiv.) and CH_2Cl_2 (10.0 mL). CCl_4 (1.3 g, 7.3 mmol, 1.2 equiv.) was diluted in CH_2Cl_2 (5.0 mL) in a separate flask and then added to the reaction mixture dropwise over 10 min at room temperature. Phenylethan-1-ol (**41**) (0.75 g, 6.1 mmol, 1.0 equiv.) was added dropwise to the reaction mixture and then stirred for 2 h. The reaction was quenched with water (10.0 mL) and extracted with CH_2Cl_2 (10 mL x 3). The organic layers were combined, dried over $MgSO_4$, and concentrated under reduced pressure. The resultant mixture was purified by silica flash chromatography (95:5 hexane:EtOAc) to yield chloride **43** as a light yellow oil (461 mg, 61% yield) as the product. **^{1}H NMR (400 MHz, CDCl$_3$).** δ = 7.43–

7.28 (m, 5H), 5.09 (q, J = 6.82 Hz, 1H), 1.85 (d, J = 6.84 Hz, 3H). Spectral data matched those reported previously.[10]

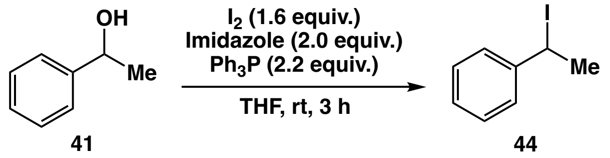

Synthesis of iodide **44**.

(1-Iodoethyl)benzene (44). To a flame-dried 20 mL vial equipped with a stir bar was added 1-phenylethan-1-ol (**41**) (500 mg, 4.09 mmol, 1.0 equiv.), imidazole (1.64 g, 8.18 mmol, 2.0 equiv.), and triphenylphosphine (1.98 g, 9.02, 2.2 equiv.) were added and dissolved in THF (8.0 mL). Iodine (1.66 g, 6.55 mmol, 1.6 eq.) was added and the reaction mixture was stirred for 3 h at room temperature. The reaction was quenched with saturated $NaHCO_3$ (10.0 mL) and extracted with CH_2Cl_2 (5.0 mL x 3). The organic layers were combined, dried over Na_2SO_4, and concentrated under reduced pressure. The resultant mixture was purified by silica flash chromatography (1:10 EtOAc:hexane) to yield iodide **44** as a light yellow oil (781 mg, 83%). The product was observed to decompose over time and was used immediately after purification. **^{1}H NMR (400 MHz, $CDCl_3$).** δ = 7.45 (d, J = 7.9 Hz, 2H), 7.30 (t, J = 14.4 Hz, 2H), 7.27–7.22 (m, 1H), 5.41 (q, 7.4 Hz, 1H), 2.22 (d, J = 6.8 Hz, 3H). Spectral data matched those reported previously.[11]

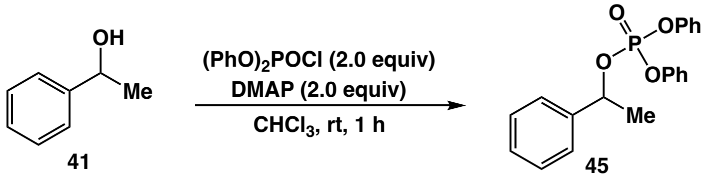

Synthesis of phosphate **45**.

Diphenyl (1-phenylethyl) phosphate (45). To a flame-dried 20 mL vial equipped with a stir bar was added 1-phenylethan-1-ol (**41**) (61.1 mg, 0.5 mmol, 1.0 equiv.) and chloroform (3.0 mL). DMAP (122.2 mg, 1.0 mmol, 2.0 equiv.) and $(PhO)_2POCl$ (268.6 mg, 1.0 mmol, 2.0 equiv.) were added, and the mixture was stirred for 1 h. The reaction was quenched with water (3.0 mL) and

extracted CH_2Cl_2 (2.0 mL x 3). The organic layers were combined, dried over Na_2SO_4, and concentrated under reduced pressure. Phosphate **45** was unstable to silica flash chromatography purification conditions and was used without further purification. **^{1}H NMR (400 MHz, $CDCl_3$).** δ = 7.89-7.75 (m, 4H), 7.69-7.61 (m, 2H), 7.53-7.27 (m, 9H), 5.55-5.48 (m, 1H), 1.67 (d, *J* = 6.2 Hz, 3H). Spectral data matched those reported previously.[12]

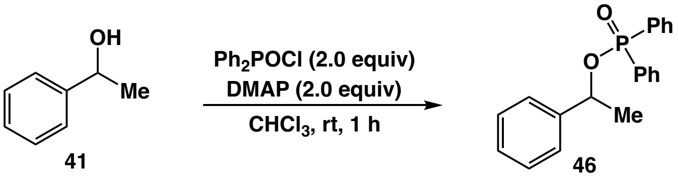

Synthesis of phosphinate **46**.

1-Phenylethyl diphenylphosphinate (46). To a flame-dried 20 mL vial equipped with a stir bar was added 1-phenylethan-1-ol (**41**) (61.1 mg, 0.5 mmol, 1.0 equiv.) and chloroform (3.0 mL). DMAP (122.2 mg, 1.0 mmol, 2.0 equiv.) and Ph_2POCl (236.6 mg, 1.0 mmol, 2.0 equiv.) were added and the mixture was stirred for 1 h. The reaction was quenched with water (3.0 mL) and extracted with CH_2Cl_2 (2.0 mL x 3). The organic layers were combined, dried over Na_2SO_4, and concentrated under reduced pressure. Phosphinate **46** was unstable to silica flash chromatography purification conditions and was used without further purification. **^{1}H NMR (400 MHz, $CDCl_3$).** δ = 7.36-7.12 (m, 13H), 7.05-6.99 (m, 2H), 5.70 (q, *J* = 6.7 Hz, 1H), 1.65 (d, *J* = 6.4 Hz, 3H). Spectral data matched those reported previously.[13]

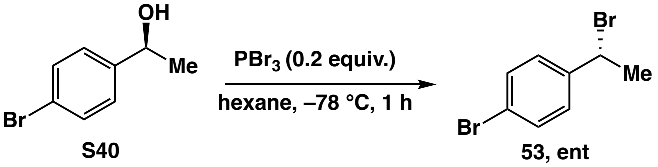

Synthesis of bromide **53, ent**.

Enantioenriched 1-Bromo-4-(1-bromoethyl)benzene (53, ent). Enantiopure alcohol **S40** was added to a flame-dried 20 mL vial equipped with a stir bar. Hexane (3.0 mL) was added and the

solution was cooled to –78 °C. PBr_3 (3 uL, 0.032 mmol, 0.2 equiv.) was added to the solution dropwise, and reaction mixture was further stirred for 1 h at –78 °C. The reaction was quenched with water (5.0 mL) and extracted with CH_2Cl_2 (3.0 mL x 3). The organic layers were combined, dried over Na_2SO_4, and concentrated under reduced pressure. The resultant oil was purified by silica flash chromatography (100% hexanes) to yield bromide **53, ent** as a colorless oil (53 mg, 80% yield, 24% ee). Chiral SFC: 250 mm ChiralPak IG–3, 5% MeOH, 3.0 mL/min, λ = 254 nm, 35 °C, nozzle pressure = 140 bar CO_2, tR1 (major) = 2.24 min, tR2 (minor) = 2.65 min. **^{1}H NMR (400 MHz, $CDCl_3$).** δ = 7.47 (d, *J* = 6. 7 Hz, 2H), 7.31 (d, *J* = 7.1 Hz, 2H), 5.15 (q, *J* = 7.3 Hz, 1H), 2.02 (d, *J* = 7.2 Hz, 3H). Spectral data matched those reported previously.[8]

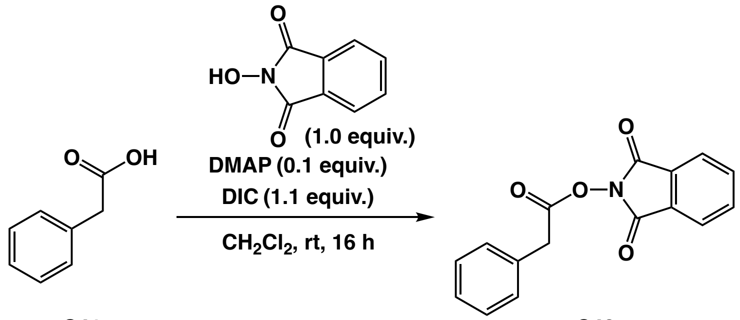

Synthesis of redox ester **S42**.

1,3-dioxoisoindolin-2-yl-2-phenylacetate (S42). To a flame-dried 25 mL round-bottom flask equipped with a stir bar was added phenylacetic acid (**S41**) (272 mg, 2.0 mmol, 1.0 equiv.) and *N*-hydroxyphthalimide (326 mg, 2.0 mmol, 1.0 equiv.). The solids were dissolved in CH_2Cl_2 (10 mL, 0.2 M) and DMAP (24.4 mg, 0.2 mmol, 0.10 equiv.) was added, followed by DIC (0.34 mL, 2.2 mmol, 1.1 equiv.). The reaction mixture was stirred at room temperature for 16 hours. Upon completion, the reaction was filtered over celite and rinsed with EtOAc (3 x 10 mL). The colorless filtrate was concentrated in vacuo. The crude material was purified by flash silica chromatography (30% EtOAc/hexanes) to provide product **S42** as a white solid (549 mg, 97% yield); Rf = 0.51 (30% EtOAc/hexanes). **^{1}H NMR (600 MHz, $CDCl_3$)** δ = 7.88 (dd, J = 5.5, 3.2 Hz, 2H), 7.78 (dd, J = 5.5, 3.1 Hz, 2H), 7.39–7.31 (m, 5H), 4.00 (s, 2H). Spectral data matched those reported previously.[14]

10. Product characterization.

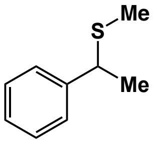

7

Methyl(1-phenylethyl)sulfane (7). Thioether **7** was synthesized following the optimized procedure outlined in section 6 with 1-(bromoethyl)benzene (**1**) (92.5 mg, 0.5 mmol, 1.0 equiv.) and dimethyl disulfide (**5**) (70.6 mg, 0.75 mmol, 67 uL, 1.5 equiv.). Thioether product **7** was obtained as a colorless liquid (74.5 mg, 98% yield) upon purification by flash chromatography on silica gel with 100% hexane. (R_f = 0.30, 100% hexanes). **^{1}H NMR (600 MHz, CDCl$_3$).** δ = 7.30–7.27 (m, 4H), 7.21–7.17 (m, 1H), 3.81 (q, *J* = 7.1 Hz, 1H), 1.86 (s, 3H), 1.54 (d, *J* = 7.0 Hz, 3H). **^{13}C NMR (150 MHz, CDCl$_3$).** δ = 143.8, 128.6, 127.4, 127.1, 45.8, 22.2, 14.7. **IR (solution, MeCN).** 2984, 2920, 1492, 1452, 1224, 956 cm^{-1}. **HRMS-QTOF-ESI** (m/z) [M + Ag]$^+$ calcd for $C_9H_{12}AgS^+$, 258.9706; found 258.9718.

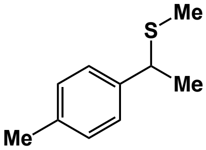

8

Methyl(1-(*p*-tolyl)ethyl)sulfane (8). Thioether **8** was synthesized following the optimized procedure outlined in section 6 with 1-(1-bromoethyl)-4-methylbenzene (99.6 mg, 0.5 mmol, 1.0 equiv.) and dimethyl disulfide (**5**) (70.6 mg, 0.75 mmol, 67 uL, 1.5 equiv.). Thioether product **8** was obtained as a colorless liquid (75.5 mg, 91% yield) upon purification by flash chromatography on silica gel with 100% hexane. (R_f = 0.30, 100% hexanes). **^{1}H NMR (600 MHz, CDCl$_3$).** δ = 7.21 (d, *J* = 8.1 Hz, 2H), 7.14 (d, *J* = 8.0 Hz, 2H), 3.83 (q, *J* = 7.1 Hz, 1H), 2.34 (s, 3H), 1.91 (s, 3H), 1.57 (d, *J* = 7.1 Hz, 3H). **^{13}C NMR (150 MHz, CDCl$_3$).** δ = 140.8, 136.8, 129.2, 127.3, 45.5, 22.3,

21.2, 14.7. **IR (solution, MeCN).** 2980, 2920, 1636, 1200, 1040, 824 cm^{-1}. **HRMS-QTOF-ESI** (m/z) $[M + Ag]^+$ calcd for $C_{10}H_{14}AgS^+$, 272.9867; found 272.9864.

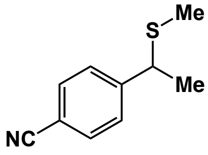

9

4-(1-(Methylthio)ethyl)benzonitrile (9). Thioether **9** was synthesized following the optimized procedure outlined in section 6 with 4-(1-bromoethyl)benzonitrile (105 mg, 0.5 mmol, 1.0 equiv.) and dimethyl disulfide (**5**) (70.6 mg, 0.75 mmol, 67 uL, 1.5 equiv.). Thioether product **9** was obtained as a colorless liquid (77 mg, 87% yield) upon purification by flash chromatography on silica gel with 100% hexane to 7:3 Hexane:EtOAc (R_{f} = 0.50, 8:2 hexanes:EtOAc). **^{1}H NMR (600 MHz, $CDCl_3$).** δ = 7.61 (d, *J* = 8.3 Hz, 2H), 7.44 (d, *J* = 8.4 Hz, 2H), 3.86 (q, *J* = 7.1 Hz, 1H), 1.90 (s, 3H), 1.57 (d, *J* = 7.1 Hz, 3H). **^{13}C NMR (150 MHz, $CDCl_3$).** δ = 149.5, 132.5, 128.2, 118.9, 110.9, 45.6, 21.8, 14.6. **IR (solution, MeCN).** 2948, 2252, 1632, 1440, 1376, 1040 cm^{-1}. **HRMS-QTOF-ESI** (m/z) $[M + Ag]^+$ calcd for $C_{10}H_{11}AgNS^+$, 283.9658; observed 318.8227 with Ag isotope pattern.

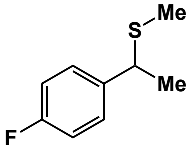

10

(1-(4-Fluorophenyl)ethyl)(methyl)sulfane (10). Thioether **10** was synthesized following the optimized procedure outlined in section 6 with 1-(1-bromoethyl)-4-fluorobenzene (102 mg, 0.5 mmol, 1.0 equiv.) and dimethyl disulfide (**5**) (70.6 mg, 0.75 mmol, 67 uL, 1.5 equiv.). Thioether product **10** was obtained as a colorless liquid (74 mg, 87% yield) upon purification by flash chromatography on silica gel with 100% hexane. (R_{f} = 0.30, 100% hexanes). **^{1}H NMR (600 MHz, $CDCl_3$).** δ = 7.31–7.28 (m, 2H), 7.01–6.98 (m, 2H), 3.84 (q, *J* = 7.1 Hz, 1H), 1.90 (s, 3H), 1.55 (d, *J* = 7.1 Hz, 3H). **^{13}C NMR (150 MHz, $CDCl_3$).** δ = 161.9 (d, *J* = 241.6 Hz), 139.6 (d, *J* = 15.1

Hz), 130.9 (d, J = 9.1 Hz), 115.4 (d, J = 15.1 Hz), 45.1, 22.4, 14.7. **^{19}F NMR (564 MHz, $CDCl_3$).** δ = –116.6. **IR (solution, MeCN).** 2940, 2252, 1628, 1440, 1376, 1036 cm^{-1}. **HRMS-QTOF-ESI** (m/z) $[M + Ag]^+$ calcd for $C_9H_{11}AgFS^+$, 276.9611; found 276. 9617.

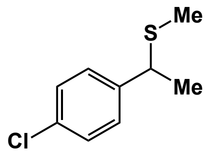

11

(1-(4-Chlorophenyl)ethyl)(methyl)sulfane (11). Thioether **11** was synthesized following the optimized procedure outlined in section 6 with 1-(1-bromoethyl)-4-chlorobenzene (110 mg, 0.5 mmol, 1.0 equiv.) and dimethyl disulfide (**5**) (70.6 mg, 0.75 mmol, 67 uL, 1.5 equiv.). Thioether product **11** was obtained as a colorless liquid (84.6 mg, 91% yield) upon purification by flash chromatography on silica gel with 100% hexane. (R_f = 0.30, 100% hexanes). **^{1}H NMR (600 MHz, $CDCl_3$).** δ = 7.28–7.20 (m, 4H), 3.81 (q, J = 7.1 Hz, 1H), 1.90 (s, 3H), 1.54 (d, J = 7.1 Hz, 3H). **^{13}C NMR (150 MHz, $CDCl_3$).** δ = 142.4, 132.7, 130.3, 128.7, 45.2, 22.2, 14.6. **IR (solution, MeCN).** 2984, 2920, 1628, 1492, 1092, 1016 cm^{-1}. **HRMS-QTOF-ESI** (m/z) $[M + Ag]^+$ calcd for $C_9H_{11}AgClS^+$, 292.9316; found 292.9314.

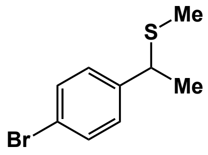

12

(1-(4-Bromophenyl)ethyl)(methyl)sulfane (12). Thioether **12** was synthesized following the optimized procedure outlined in section 6 with 1-bromo-4-(1-bromoethyl)benzene (132 mg, 0.5 mmol, 1.0 equiv.) and dimethyl disulfide (**5**) (70.6 mg, 0.75 mmol, 67 uL, 1.5 equiv.). Thioether product **12** was obtained as a colorless liquid (96.6 mg, 84% yield) upon purification by flash chromatography on silica gel with 100% hexane. (R_f = 0.30, 100% hexanes). **^{1}H NMR (600 MHz, $CDCl_3$).** δ = 7.43 (d, J = 8.4 Hz, 2H), 7.21 (d, J = 8.4 Hz, 2H), 3.80 (q, J = 7.1 Hz, 1H), 1.90 (s, 3H), 1.54 (d, J = 7.1 Hz, 3H). **^{13}C NMR (150 MHz, $CDCl_3$).** δ = 142.9, 131.7, 129.1, 120.8, 45.2,

22.1, 14.6. **IR (solution, MeCN).** 2960, 1632, 1488, 1072, 1012, 832 cm^{-1}. **HRMS-QTOF-ESI** (m/z) $[M + Ag]^+$ calcd for $C_9H_{11}AgBrS^+$, 336.8811; found 336.8801.

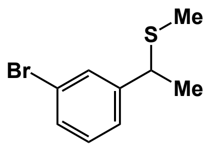

13

(1-(3-Bromophenyl)ethyl)(methyl)sulfane (13). Thioether **13** was synthesized following the optimized procedure outlined in section 6 with 1-bromo-3-(1-bromoethyl)benzene (132 mg, 0.5 mmol, 1.0 equiv.) and dimethyl disulfide (**5**) (70.6 mg, 0.75 mmol, 67 uL, 1.5 equiv.). Thioether product **13** was obtained as a colorless liquid (91 mg, 79% yield) upon purification by flash chromatography on silica gel with 100% hexane. ($R_{f=}$ 0.30, 100% hexanes). **^{1}H NMR (600 MHz, $CDCl_3$).** δ = 7.48 (t, *J* = 1.9 Hz, 1H), 7.36 (ddd, *J* = 7.8, 2.0, 1.1 Hz, 1H), 7.27–7.25 (m, 1H), 7.19 (t, *J* = 7.8 Hz, 1H), 3.79 (q, *J* = 7.1 Hz, 1H), 1.91 (s, 3H), 1.55 (d, *J* = 7.1 Hz, 3H). **^{13}C NMR (150 MHz, $CDCl_3$).** δ = 146.3, 130.5, 130.3, 130.1, 126.1, 122.6, 45.4, 22.2, 14.7. **IR (solution, MeCN).** 2896, 2532, 2416, 1628, 1568, 1472 cm^{-1}. **HRMS-QTOF-ESI** (m/z) $[M + Ag]^+$ calcd for $C_9H_{11}AgBrS^+$, 336.8811; found 336.8803.

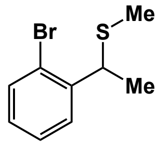

14

(1-(2-Bromophenyl)ethyl)(methyl)sulfane (14). Thioether **14** was synthesized following the optimized procedure outlined in section 6 with 1-bromo-2-(1-bromoethyl)benzene (132 mg, 0.5 mmol, 1.0 equiv.) and dimethyl disulfide (**5**) (70.6 mg, 0.75 mmol, 67 uL, 1.5 equiv.). Thioether product **14** was obtained as a colorless liquid (84 mg, 73% yield) upon purification by flash chromatography on silica gel with 100% hexane. ($R_{f=}$ 0.30, 100% hexanes). **^{1}H NMR (600 MHz, $CDCl_3$).** δ = 7.61–7.55 (m, 2H), 7.32 (t, *J* = 7.5 Hz, 1H), 7.11–7.07 (m, 1H), 4.47 (q, *J* = 7.4 Hz, 1H), 1.95 (s, 3H), 1.53 (d, *J* = 7.1 Hz, 3H). **^{13}C NMR (150 MHz, $CDCl_3$).** δ = 142.7, 132.8, 128.5,

128.4, 128.0, 124.3, 44.0, 21.7, 14.5. **IR (solution, MeCN).** 2876, 2752, 1716, 1468, 1436, 1224 cm^{-1}. **HRMS-QTOF-ESI** (m/z) $[M + Ag]^+$ calcd for $C_9H_{11}AgBrS^+$, 336.8811; found 336.8823.

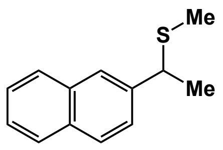

15

Methyl(1-(naphthalen-2-yl)ethyl)sulfane (15). Thioether **15** was synthesized following the optimized procedure outlined in section 6 with 2-(1-bromoethyl)naphthalene (118 mg, 0.5 mmol, 1.0 equiv.) and dimethyl disulfide (**5**) (70.6 mg, 0.75 mmol, 67 uL, 1.5 equiv.). Thioether product **15** was obtained as a colorless liquid (84 mg, 83% yield) upon purification by flash chromatography on silica gel with 100% hexane. ($R_{f=}$ 0.30, 100% hexanes). **^{1}H NMR (600 MHz, $CDCl_3$).** δ = 7.84–7.80 (m, 3H), 7.69 (s, 1H), 7.55 (dd, J = 8.5, 1.9 Hz, 1H), 7.49–7.43 (m, 2H), 4.02 (q, J = 7.1 Hz, 1H), 1.90 (s, 3H), 1.67 (d, J = 7.1 Hz, 3H). **^{13}C NMR (150 MHz, $CDCl_3$).** δ = 141.1, 133.3, 132.9, 128.6, 127.9, 127.8, 126.3, 126.0, 125.9, 125.7, 46.1, 22.1, 14.6. **IR (solution, MeCN).** 2940, 2532, 1376, 1200, 956, 904 cm^{-1}. **HRMS-QTOF-ESI** (m/z) $[M + Ag]^+$ calcd for $C_{13}H_{14}AgS^+$, 308.9862; found 308.9867.

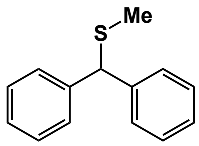

16

Benzhydryl(methyl)sulfane (16). Thioether **16** was synthesized following the optimized procedure outlined in section 6 with (bromomethylene)dibenzene (124 mg, 0.5 mmol, 1.0 equiv.) and dimethyl disulfide (**5**) (70.6 mg, 0.75 mmol, 67 uL, 1.5 equiv.). Thioether product **16** was obtained as a colorless liquid (102 mg, 95% yield) upon purification by flash chromatography on silica gel with 100% hexane. ($R_{f=}$ 0.30, 100% hexanes). **^{1}H NMR (600 MHz, $CDCl_3$).** δ = 7.44–7.41 (m, 4H), 7.35–7.30 (m, 4H), 7.24 (dddd, J = 9.0, 7.5, 3.6, 1.5 Hz, 2H), 5.08 (s, 1H), 2.01 (s, 3H), **^{13}C NMR (150 MHz, $CDCl_3$).** δ = 141.1, 133.3, 132.9, 128.6, 127,9, 127.8, 126.3, 126.0,

125.9, 125.6, 46.1, 22.1, 14.6. **IR (solution, MeCN).** 2900, 2872, 2600, 1496, 1448, 1200 cm^{-1}. **HRMS-QTOF-ESI** (m/z) $[M + Ag]^+$ calcd for $C_{14}H_{14}AgS^+$, 320.9862; found 320.9873.

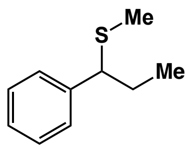

17

Methyl(1-phenylpropyl)sulfane (17). Thioether **17** was synthesized following the optimized procedure outlined in section 6 with (1-bromopropyl)benzene (100 mg, 0.5 mmol, 1.0 equiv.) and dimethyl disulfide (**5**) (70.6 mg, 0.75 mmol, 67 uL, 1.5 equiv.). Thioether product **17** was obtained as a colorless liquid (66.4 mg, 80% yield) upon purification by flash chromatography on silica gel with 100% hexane. (R_f = 0.30, 100% hexanes). **^{1}H NMR (600 MHz, $CDCl_3$).** δ = 7.33–7.27 (m, 4H), 7.24–7.21 (m, 1H), 3.60 (dd, J = 8.5, 6.3 Hz, 1H), 2.01–1.90 (m, 2H), 1.86 (s, 3H), 0.92 (t, J = 7.4, 3H) **^{13}C NMR (150 MHz, $CDCl_3$).** δ = 142.5, 128.5, 128.0, 127.1, 53.5, 29.3, 14.5, 12.5. **IR (solution, acetone).** 2872, 2488, 2372, 1716, 1360, 1216 cm^{-1}. **HRMS-QTOF-ESI** (m/z) $[M + Ag]^+$ calcd for $C_{10}H_{14}AgS^+$, 272.9862; found 272.9866.

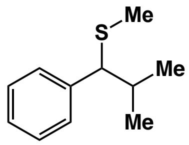

18

Methyl(2-methyl-1-phenylpropyl)sulfane (18). Thioether **18** was synthesized following the optimized procedure outlined in section 6 with (1-bromo-2-methylpropyl)benzene (107 mg, 0.5 mmol, 1.0 equiv.) and dimethyl disulfide (**5**) (70.6 mg, 0.75 mmol, 67 uL, 1.5 equiv.). Thioether product **18** was obtained as a colorless liquid (56.7 mg, 63% yield) upon purification by flash chromatography on silica gel with 100% hexane. (R_f = 0.30, 100% hexanes). **^{1}H NMR (600 MHz, $CDCl_3$).** δ = 7.32–7.26 (m, 4H), 7.23–7.21 (m, 1H), 3.44 (d, J = 8.3 Hz, 1H), 2.08 (dhept, J = 8.4, 6.7 Hz, 1H), 1.80 (s, 3H), 1.10 (d, J = 6.6, 3H), 0.82 (d, J = 6.7, 3H) **^{13}C NMR (150 MHz, $CDCl_3$).** δ = 141.9, 128.7, 128.3, 126.9, 60.0, 34.0, 21.45, 21.17, 14.8. **IR (solution, acetone).** 2940, 2744,

2252, 1464, 1040, 920 cm^{-1}. **HRMS-QTOF-ESI** (m/z) $[M + Ag]^+$ calcd for $C_{11}H_{16}AgS^+$, 287.0019; found 287.0019.

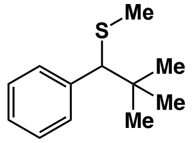

19

(2,2-Dimethyl-1-phenylpropyl)(methyl)sulfane (19). Thioether **19** was synthesized following the optimized procedure outlined in section 6 with (1-bromo-2,2-dimethylpropyl)benzene (114 mg, 0.5 mmol, 1.0 equiv.) and dimethyl disulfide (**5**) (70.6 mg, 0.75 mmol, 67 uL, 1.5 equiv.). Thioether product **19** was obtained as a colorless liquid (50.5 mg, 52% yield) upon purification by flash chromatography on silica gel with 100% hexane. (R_f = 0.30, 100% hexanes). **^{1}H NMR (600 MHz, $CDCl_3$).** δ = 7.35 (d, J = 7.5 Hz, 2H), 7.29 (t, J = 7.6 Hz, 2H), 7.23 (t, J = 7.3 Hz, 1H), 3.56 (s, 1H), 1.78 (s, 3H), 1.01 (s, 9H) **^{13}C NMR (150 MHz, $CDCl_3$).** δ = 140.6, 129.9, 127.8, 126.9, 65.1, 35.7, 28.6, 15.4. **IR (solution, acetone).** 2924, 2624, 2364, 1708, 1360, 1216 cm^{-1}. **HRMS-QTOF-ESI** (m/z) $[M + Ag]^+$ calcd for $C_{12}H_{18}AgS^+$, 301.0175; found 301.0179.

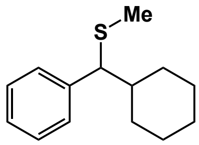

20

(Cyclohexyl(phenyl)methyl)(methyl)sulfane (20). Thioether **20** was synthesized following the optimized procedure outlined in section 6 with (bromo(cyclohexyl)methyl)benzene (127 mg, 0.5 mmol, 1.0 equiv.) and dimethyl disulfide (**5**) (70.6 mg, 0.75 mmol, 67 uL, 1.5 equiv.). Thioether product **20** was obtained as a colorless liquid (74.8 mg, 68% yield) upon purification by flash chromatography on silica gel with 100% hexane. (R_f = 0.30, 100% hexanes). **^{1}H NMR (600 MHz, $CDCl_3$).** δ = 7.32–7.25 (m, 4H), 7.22 (t, J = 7.0 Hz, 1H), 3.48 (d, J = 8.6 Hz, 1H), 2.18–2.12 (m, 1H), 1.78 (s, 3H), 1.77–1.58 (m, 4H), 1.51–1.21 (m, 2H) 1.16–0.84 (m, 4H), **^{13}C NMR (150 MHz, $CDCl_3$).** δ = 141.8, 128.8, 128.3, 126.9, 59.0, 43.3, 31.9, 31.5, 26.5, 26.4, 26.3, 14.7. **IR (solution,**

acetone). 2928, 2824, 2484, 1708, 1648, 1136 cm^{-1}. **HRMS-QTOF-ESI** (m/z) $[M + Ag]^+$ calcd for $C_{14}H_{20}AgS^+$, 327.0332; found 327.0338.

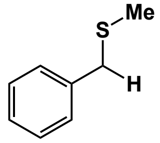

21

Benzyl(methyl)sulfane (21). Thioether **21** was synthesized following the optimized procedure outlined in section 6 with (bromomethyl)benzene (86 mg, 0.5 mmol, 1.0 equiv.) and dimethyl disulfide (**5**) (70.6 mg, 0.75 mmol, 67 uL, 1.5 equiv.). Thioether product **21** was obtained as a colorless liquid (63 mg, 91% yield) upon purification by flash chromatography on silica gel with 100% hexane. (R_f = 0.30, 100% hexanes). **^{1}H NMR (600 MHz, $CDCl_3$).** δ = 7.34–7.30 (m, 4H), 7.25–7.23 (m, 1H), 3.68 (s, 2H), 2.00 (s, 3H), **^{13}C NMR (150 MHz, $CDCl_3$).** δ = 138.4, 129.0, 128.6, 127.1, 38.5, 15.1. **IR (solution, acetone).** 2936, 2880, 2364, 1708, 1640, 1136 cm^{-1}. **HRMS-QTOF-ESI** (m/z) $[M + Ag]^+$ calcd for $C_8H_{10}AgS^+$, 244.9549; found 244.9546.

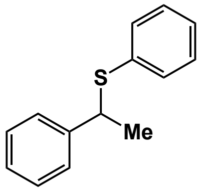

22

Phenyl(1-phenylethyl)sulfane (22). Thioether **22** was synthesized following the optimized procedure outlined in section 6 with 1-(bromoethyl)benzene (**1**) (92.5 mg, 0.5 mmol, 1.0 equiv.) and diphenyl disulfide (164 mg, 0.75 mmol, 1.0 equiv.). Thioether product **22** was obtained as a colorless liquid (98.4 mg, 92% yield) upon purification by flash chromatography on silica gel with 100% hexane. (R_f = 0.30, 100% hexanes). **^{1}H NMR (600 MHz, $CDCl_3$).** δ = 7.31–7.18 (m, 10H), 4.35 (q, J = 7.0 Hz, 1H), 1.64 (d, J = 7.0 Hz, 3H), **^{13}C NMR (150 MHz, $CDCl_3$).** δ = 143.4, 135.3,

132.6, 128.8, 128.5, 127.4, 127.3, 127.2, 48.2, 22.5. Spectral data matched those reported previously.[15]

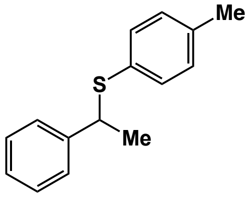

23

(1-Phenylethyl)(*p*-tolyl)sulfane (23). Thioether **23** was synthesized following the optimized procedure outlined in section 6 with 1-(bromoethyl)benzene (**1**) (92.5 mg, 0.5 mmol, 1.0 equiv.) and *p*-tolyl disulfide (185 mg, 0.75 mmol, 1.5 equiv.). Thioether product **23** was obtained as a colorless liquid (103 mg, 88% yield) upon purification by flash chromatography on silica gel with 100% hexane. (R_f = 0.30, 100% hexanes). **^{1}H NMR (600 MHz, $CDCl_3$).** δ = 7.30–7.23 (m, 4H), 7.22–7.19 (m,1H), 7.18 (d, J = 12.0 Hz, 2H), 7.03 (d, J = 6.0 Hz, 2H), 4.26 (q, J = 7.0 Hz, 1H), 2.29 (s, 3H), 1.61 (d, J = 7.0 Hz, 3H), **^{13}C NMR (150 MHz, $CDCl_3$).** δ = 143.5, 137.5, 133.6, 131.5, 129.6, 128.5, 127.4, 127.2, 48.5, 22.3, 21.2. Spectral data matched those reported previously.[15]

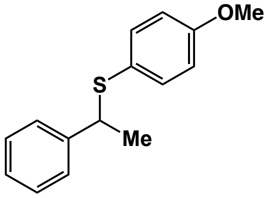

24

(4-Methoxyphenyl)(1-phenylethyl)sulfane (24). Thioether **24** was synthesized following the optimized procedure outlined in section 6 with 1-(bromoethyl)benzene (**1**) (92.5 mg, 0.5 mmol, 1.0 equiv.) and bis(4-methoxyphenyl) disulfide (209 mg, 0.75 mmol, 1.5 equiv.). Thioether product **24** was obtained as a colorless liquid (106 mg, 87% yield) upon purification by flash chromatography on silica gel with 100% hexane to 8:2 hexane:EtOAc. (R_f = 0.40, hexanes:EtOAc 9:1). **^{1}H NMR (600 MHz, $CDCl_3$).** δ = 7.28–7.16 (m, 7H), 6.76 (d, J = 8.5 Hz, 2H), 4.17 (q, J =

7.0 Hz, 1H), 3.77 (s, 3H), 1.60 (d, *J* = 7.1 Hz, 3H). Spectral data matched those reported previously.[15]

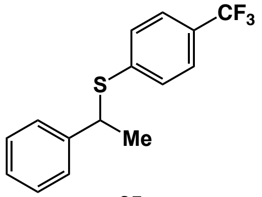

25

(1-Phenylethyl)(4-(trifluoromethyl)phenyl)sulfane (25). Thioether **25** was synthesized following the optimized procedure outlined in section 6 with 1-(bromoethyl)benzene (**1**) (92.5 mg, 0.5 mmol, 1.0 equiv.) and bis(4-trifluoromethyl)phenyl disulfide (266 mg, 0.75 mmol, 1.5 equiv.). Thioether product **25** was obtained as a colorless liquid (117 mg, 83% yield) upon purification by flash chromatography on silica gel with 100% hexane. ($R_{f=}$ 0.30, 100% hexanes). **^{1}H NMR (600 MHz, $CDCl_3$).** δ = 7.44 (d, *J* = 8.0 Hz, 2H), 7.35 (d, *J* = 6.9 Hz, 2H), 7.31 (dd, *J* = 17.8, 7.8 Hz, 4H), 7.24 (t, *J* = 7.2 Hz, 1H), 4.46 (q, *J* = 7.0 Hz, 1H), 1.67 (d, *J* = 7.0 Hz, 3H), **^{13}C NMR (150 MHz, $CDCl_3$).** δ = 142.8, 141.1, 130.5, 128.8, 128.5 (q, *J* = 32.8 Hz), 127.6, 127.3, 125.6 (q, *J* = 3.02 Hz), 124.2 (q, *J* = 273.3 Hz), 47.3, 22.8. **^{19}F NMR (564 MHz, $CDCl_3$).** δ = –62.6. Spectral data matched those reported previously.[15]

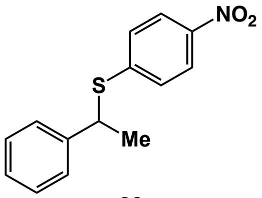

26

(4-Nitrophenyl)(1-phenylethyl)sulfane (26). Thioether **26** was synthesized following the optimized procedure outlined in section 6 with 1-(bromoethyl)benzene (**1**) (92.5 mg, 0.5 mmol, 1.0 equiv.) and bis(4-nitrophenyl) disulfide (231 mg, 0.75 mmol, 1.5 equiv.). Thioether product **26** was obtained as a colorless liquid (111 mg, 85% yield) upon purification by flash chromatography on silica gel with 100% hexane to 8:2 hexane:EtOAc. **^{1}H NMR (600 MHz,**

$CDCl_3$). δ = 8.03 (d, *J* = 8.8 Hz, 2H), 7.43 (d, *J* = 7.6 Hz, 2H), 7.40–7.22 (m, 5H), 4.56 (q, *J* = 7.0 Hz, 1H), 1.69 (d, *J* = 7.1 Hz, 3H). Spectral data matched those reported previously.[16]

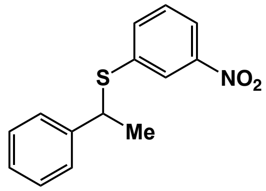

27

(3-Nitrophenyl)(1-phenylethyl)sulfane (27). Thioether **27** was synthesized following the optimized procedure outlined in section 6 with 1-(bromoethyl)benzene (**1**) (92.5 mg, 0.5 mmol, 1.0 equiv.) and bis(3-nitrophenyl) disulfide (231 mg, 0.75 mmol, 1.5 equiv.). Thioether product **27** was obtained as a colorless liquid (117.8 mg, 91% yield) upon purification by flash chromatography on silica gel with 100% hexane to 9:1 hexane:EtOAc. (R_{f} = 0.57, hexane:EtOAc 9:1). **^{1}H NMR (500 MHz, $CDCl_3$).** δ = 8.09 (t, *J* = 1.98 Hz, 1H), 7.99 (ddd, *J* = 8.3, 2.2, 1.0 Hz, 1H), 7.51 (ddd, *J* = 7.8, 1.8, 1.1 Hz, 1H), 7.36–7.18 (m, 6H), 4.46 (q, *J* = 7.0 Hz, 1H), 1.68 (d, *J* = 7.1 Hz, 3H), **^{13}C NMR (125 MHz, $CDCl_3$).** 11 of 12 signals observed: δ = 142.3, 138.4, 137.2, 129.4, 128.8, 127.8, 127.4, 125.9, 121.7, 47.9, 22.5**. IR (solution, MeCN).** 2976, 2908, 2724, 1532, 1352, 1064 cm^{-1}. **HRMS-QTOF-ESI** (m/z), $[M + Ag]^+$ calcd for $C_{14}H_{13}AgNO_2S^+$, 365.9713; observed 388.1792 with Ag isotope pattern.

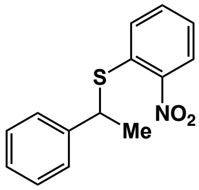

28

(2-Nitrophenyl)(1-phenylethyl)sulfane (28). Thioether **28** was synthesized following the optimized procedure outlined in section 6 with 1-(bromoethyl)benzene (**1**) (92.5 mg, 0.5 mmol, 1.0 equiv.) and bis(2-nitrophenyl) disulfide (231 mg, 0.75 mmol, 1.5 equiv.). Thioether product **28** was obtained as a light yellow solid (75.1 mg, 58% yield) upon purification by flash chromatography on silica gel with 100% hexane to 8:2 hexane:EtOAc. **^{1}H NMR (600 MHz,**

$CDCl_3$). δ = 8.06 (d, *J* = 8.0 Hz, 1H), 7.43–7.34 (m, 4H), 7.32–7.30 (m, 2H), 7.24–7.18 (m, 2H), 4.55 (q, *J* = 7.0 Hz, 1H), 1.70 (d, *J* = 6.9 Hz, 3H). Spectral data matched those reported previously.[17]

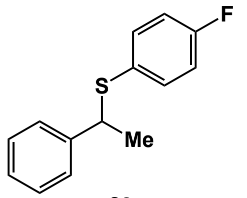

29

(4-Fluorophenyl)(1-phenylethyl)sulfane (29). Thioether **29** was synthesized following the optimized procedure outlined in section 6 with 1-(bromoethyl)benzene (**1**) (92.5 mg, 0.5 mmol, 1.0 equiv.) and bis(4-fluorophenyl) disulfide (191 mg, 0.75 mmol, 1.5 equiv.). Thioether product **29** was obtained as a colorless liquid (75.4 mg, 65% yield) upon purification by flash chromatography on silica gel with 100% hexane. (R_{f} = 0.30, 100% hexanes). **^{1}H NMR (600 MHz, $CDCl_3$).** δ = 7.29–7.23 (m, 3H), 7.22–7.18 (m, 4H), 6.91 (t, *J* = 8.7 Hz, 2H), 4.23 (q, *J* = 7.0 Hz, 1H), 1.62 (d, *J* = 6.7 Hz, 3H), **^{13}C NMR (150 MHz, $CDCl_3$).** δ = 162.7 (d, *J* = 241.6 Hz), 143.2, 135.8 (d, *J* = 15.1 Hz), 129.9 (d, *J* = 3.0 Hz), 128.5, 127.4, 127.3, 115.8 (d, *J* = 15.1 Hz), 49.1, 22.1. **^{19}F NMR (564 MHz, $CDCl_3$).** δ = –114.0. Spectral data matched those reported previously.[18]

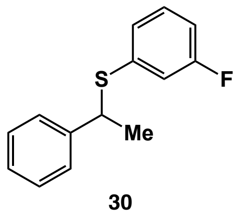

30

(3-Fluorophenyl)(1-phenylethyl)sulfane (30). Thioether **30** was synthesized following the optimized procedure outlined in section 6 with 1-(bromoethyl)benzene (**1**) (92.5 mg, 0.5 mmol, 1.0 equiv.) and bis(3-fluorophenyl) disulfide (191 mg, 0.75 mmol, 1.5 equiv.). Thioether product **30** was obtained as a colorless liquid (91.6 mg, 79% yield) upon purification by flash chromatography on silica gel with 100% hexane. (R_{f} = 0.30, 100% hexanes). **^{1}H NMR (600 MHz, $CDCl_3$).** δ = 7.33–7.28 (m, 4H), 7.23 (t, *J* = 7.1 Hz, 1H), 7.18 (td, *J* = 8.1, 8.0, 6.1 Hz, 1H), 7.05

(ddd, *J* = 7.8, 1.7, 0.9 Hz, 1H), 6.99 (ddd, *J* = 9.4, 2.6, 1.6 Hz, 1H), 6.88 (tdd, *J* = 8.4, 2.6, 1.0 Hz, 1H), 4.39 (q, *J* = 7.0 Hz, 1H), 1.65 (d, *J* = 7.1 Hz, 3H), **^{13}C NMR (150 MHz, $CDCl_3$).** δ = 162.7 (d, *J* = 247.6 Hz), 142.9, 137.8 (d, *J* = 7.6 Hz), 130.0 (d, *J* = 18.1 Hz), 128.7, 127.5, 127.4 (d, *J* = 3.0 Hz), 127.3, 118.5 (d, *J* = 22.7 Hz), 114.0 (d, *J* = 21.1 Hz), 47.9, 22.6. **^{19}F NMR (564 MHz, $CDCl_3$).** δ = –112.7. **IR (solution, acetone).** 2944, 2368, 2336, 1712, 1596, 1580 cm^{-1}. **HRMS-QTOF-ESI** (m/z) $[M + Ag]^+$ calcd for $C_{14}H_{13}AgFS^+$, 338.9768; found 338.9763.

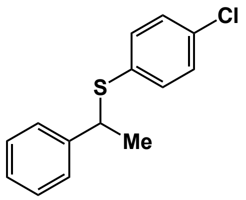

31

(4-Chlorophenyl)(1-phenylethyl)sulfane (31). Thioether **31** was synthesized following the optimized procedure outlined in section 6 with 1-(bromoethyl)benzene (**1**) (92.5 mg, 0.5 mmol, 1.0 equiv.) and bis(4-chlorophenyl) disulfide (215 mg, 0.75 mmol, 1.5 equiv.). Thioether product **31** was obtained as a colorless liquid (116.6 mg, 94% yield) upon purification by flash chromatography on silica gel with 100% hexane. (R_f = 0.30, 100% hexanes). **^{1}H NMR (600 MHz, $CDCl_3$).** δ = 7.30–7.27 (m, 4H), 7.23–7.20 (m, 1H), 7.19–7.15 (m, 4H), 4.30 (q, *J* = 7.0 Hz, 1H), 1.63 (d, *J* = 7.1 Hz, 3H), **^{13}C NMR (150 MHz, $CDCl_3$).** δ = 143.0, 134.1, 133.7, 133.5, 129.0, 128.6, 127.4, 127.4, 48.4, 22.3. Spectral data matched those reported previously.[15]

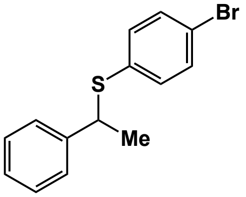

32

(4-Bromophenyl)(1-phenylethyl)sulfane (32). Thioether **32** was synthesized following the optimized procedure outlined in section 6 with 1-(bromoethyl)benzene (**1**) (92.5 mg, 0.5 mmol, 1.0 equiv.) and bis(4-bromophenyl) disulfide (282 mg, 0.75 mmol, 1.5 equiv.). Thioether product

32 was obtained as a colorless liquid (129.5 mg, 89% yield) upon purification by flash chromatography on silica gel with 100% hexane. (R_f = 0.30, 100% hexanes). **^{1}H NMR (600 MHz, $CDCl_3$).** δ = 7.33 (d, *J* = 8.3 Hz, 2H), 7.30–7.27 (m, 4H), 7.24–7.20 (m, 1H), 7.12 (d, *J* = 8.5 Hz, 2H), 4.31 (q, *J* = 7.0 Hz, 1H), 1.63 (d, *J* = 7.0 Hz, 3H), **^{13}C NMR (150 MHz, $CDCl_3$).** δ = 143.0, 134.4, 134.2, 131.9, 128.6, 127.4, 127.3 121.5, 48.3, 22.4. Spectral data matched those reported previously.[15]

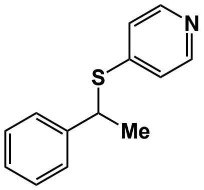

33

4-((1-Phenylethyl)thio)pyridine (33). Thioether **33** was synthesized following the optimized procedure outlined in section 6 with 1-(bromoethyl)benzene (**1**) (92.5 mg, 0.5 mmol, 1.0 equiv.) and 4,4-dithiodipyridine (165 mg, 0.75 mmol, 1.5 equiv.). Thioether product **33** was obtained as a colorless liquid (69.9 mg, 65% yield) upon purification by flash chromatography on silica gel with 100% hexane to 8:2 hexane:EtOAc. (R_f = 0.51, hexanes:EtOAc 6:4). **^{1}H NMR (500 MHz, $CDCl_3$).** δ = 8.35 (d, *J* = 6.3 Hz, 2H), 7.44 (d, *J* = 7.0 Hz, 2H), 7.35 (t, *J* = 7.5 Hz, 2H), 7.28 (t, *J* = 3.7 Hz, 1H), 7.09 (d, *J* = 6.2 Hz, 2H), 4.59 (q, *J* = 7.1 Hz, 1H), 1.71 (d, *J* = 7.1 Hz, 3H), **^{13}C NMR (125 MHz, $CDCl_3$).** δ = 149.4, 148.4, 142.3, 128.9, 127.8, 127.2, 122.2, 45.1, 23.1. **IR (solution, acetone).** 2864, 2640, 1576, 1488, 1244, 1048 cm^{-1}. **HRMS-QTOF-ESI** (m/z) $[M + Ag]^+$ calcd for $C_{13}H_{13}AgNS^+$, 321.9815; found 321.9822.

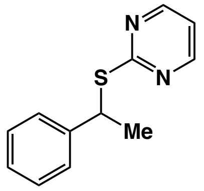

34

2-((1-phenylethyl)thio)pyrimidine (34). Thioether **34** was synthesized following the optimized procedure outlined in section 6 with 1-(bromoethyl)benzene (**1**) (92.5 mg, 0.5 mmol, 1.0 equiv.) and 2-pyrimidine disulfide (167 mg, 0.75 mmol, 1.5 equiv.). Thioether product **34** was obtained

as a colorless liquid (75.5 mg, 70% yield) upon purification by flash chromatography on silica gel with 100% CH_2Cl_2. (R_f = 0.77, 100% CH_2Cl_2). **^{1}H NMR (400 MHz, $CDCl_3$).** δ = 8.52 (dd, *J* = 4.9, 1.8 Hz, 2H), 7.50 (d, *J* = 7.7 Hz, 2H), 7.34 (t, *J* = 7.2 Hz, 2H), 7.28–7.24 (m, 1H), 6.95 (td, *J* = 5.0, 1.8 Hz, 1H), 5.10 (q, *J* = 7.2 Hz, 1H), 1.80 (d, *J* = 7.1 Hz, 3H). Spectral data matched those reported previously.[19]

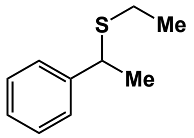

35

Ethyl(1-phenylethyl)sulfane (35). Thioether **35** was synthesized following the optimized procedure outlined in section 6 with 1-(bromoethyl)benzene (**1**) (92.5 mg, 0.5 mmol, 1.0 equiv.) and diethyl disulfide (91.6 mg, 0.75 mmol, 1.5 equiv.). Thioether product **35** was obtained as a colorless liquid (67.2 mg, 81% yield) upon purification by flash chromatography on silica gel with 100% hexane. (R_f = 0.30, 100% hexanes). **^{1}H NMR (600 MHz, $CDCl_3$).** δ = 7.30–7.19 (m, 5H), 3.97 (q, *J* = 7.0 Hz, 1H), 2.22 (q, *J* = 7.4 Hz, 2H), 1.56 (d, *J* = 7.0 Hz, 3H), 1.16 (t, *J* = 7.4 Hz, 3H). Spectral data matched those reported previously.[16]

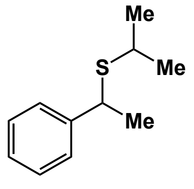

36

Isopropyl(1-phenylethyl)sulfane (36). Thioether **36** was synthesized following the optimized procedure outlined in section 6 with 1-(bromoethyl)benzene (**1**) (92.5 mg, 0.5 mmol, 1.0 equiv.) and diisopropyl disulfide (112.7 mg, 0.75 mmol, 1.5 equiv.). Thioether product **36** was obtained as a colorless liquid (46.8 mg, 52% yield) upon purification by flash chromatography on silica gel with 100% hexane. (R_f = 0.30, 100% hexanes). **^{1}H NMR (600 MHz, $CDCl_3$).** δ = 7.30–7.17 (m,

5H), 4.02 (q, J = 7.1 Hz, 1H), 2.51–2.45 (m, 1H), 1.55 (d, J = 7.0 Hz, 3H), 1.13 (dd, J = 6.7, 4.5 Hz, 6H). Spectral data matched those reported previously.[20]

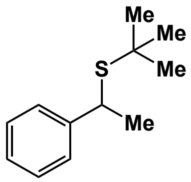

37

***tert*-Butyl(1-phenylethyl)sulfane (37).** Thioether **37** was synthesized following the optimized procedure outlined in section 6 with 1-(bromoethyl)benzene (**1**) (92.5 mg, 0.5 mmol, 1.0 equiv.) and di-*tert*-butyl disulfide (133.7 mg, 0.75 mmol, 1.5 equiv.). Thioether product **37** was obtained as a colorless liquid (35.8 mg, 37% yield) after purification by flash chromatography on silica gel with 100% hexane. (R_{f} = 0.30, 100% hexanes). **^{1}H NMR (600 MHz, $CDCl_3$).** δ = 7.34–7.20 (m, 5H), 3.97 (q, J = 6.9 Hz, 1H), 1.57 (d, J = 7.0 Hz, 3H), 1.22 (s, 9H). Spectral data matched those reported previously.[21]

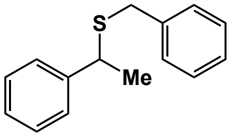

38

Benzyl(1-phenylethyl)sulfane (38). Thioether **38** was synthesized following the optimized procedure outlined in section 6 with 1-(bromoethyl)benzene (**1**) (92.5 mg, 0.5 mmol, 1.0 equiv.) and dibenzyl disulfide (185 mg, 0.75 mmol, 1.5 equiv.). Thioether product **38** was obtained as a colorless liquid (99.2 mg, 87% yield) after purification by flash chromatography on silica gel with 100% hexane. (R_{f} = 0.30, 100% hexanes). **^{1}H NMR (600 MHz, $CDCl_3$).** δ = 7.34–7.19 (m, 10H),

3.82 (q, *J* = 7.1 Hz, 1H), 3.51 (dd, *J* = 24.8, 14.0 Hz, 2H), 1.54 (d, *J* = 7.1 Hz, 3H) Spectral data matched those reported previously.[15]

11. Synthetic application procedures.

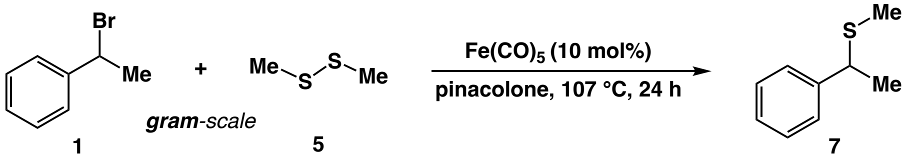

Gram-scale reaction.

Gram-Scale Procedure. To a flame-dried 20 mL dram vial equipped with a stir bar was added $Fe(CO)_5$ (53 mg, 0.27 mmol, 37 uL, 5 mol%) in a glove box. The vial was taken out of the glove box and placed under N_2 in a fume-hood. To the vial was added pinacolone (10 mL), (1-bromoethyl)benzene (**1**) (1 g, 5.4 mmol, 737 uL, 1.0 equiv.), and dimethyl disulfide (**5**) (763 mg, 8.1 mmol, 730 uL, 1.5 equiv.). The reaction vessel was sealed with a teflon screw cap and the mixture was stirred at 107 °C. The reaction mixture was cooled to room temperature after 12 hours, and an additional portion of $Fe(CO)_5$ (53 mg, 0.27 mmol, 37 uL, 5 mol%) from the glove box was added via syringe. The reaction was stirred for an additional 12 h at 107 °C. The mixture was quenched with water (5.0 mL) and the aqueous layer was extracted with diethyl ether (3 x 5.0 mL). The organic layers were combined, dried over anhydrous sodium sulfate, and concentrated under reduced pressure. The resultant oil was purified by silica flash chromatography (100% hexanes) to yield thioether **7** as a colorless oil (741 mg, 98% yield).

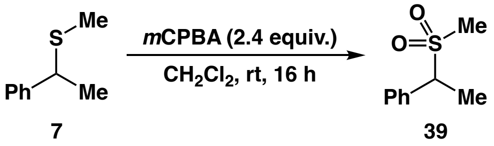

Sulfone **39** synthesis.

(1-(Methylsulfonyl)ethyl)benzene (39). To a flame-dried 20 mL vial equipped with a stir bar was added methyl(1-phenylethyl)sulfane (**7**) (50 mg, 0.33 mmol, 1.0 equiv.) and CH_2Cl_2 (2.5 mL). *m*CPBA (136 mg, 0.8 mmol, 2.4 equiv.) was dissolved in CH_2Cl_2 (1.0 mL) in a separate vial and subsequently added to the reaction vessel dropwise for 5 min at room temperature. The mixture was further stirred for 16 h, then quenched with water (2.0 mL) and extracted with CH_2Cl_2 (2.0

mL x 3). The organic layers were combined, dried over Na_2SO_4, and concentrated under reduced pressure. The resultant mixture was purified with silica flash chromatography (1:3 EtOAc:hexane to pure methanol) to yield product **39** as a colorless crystal (56.4 mg, 93%). **^{1}H NMR (400 MHz, $CDCl_3$)** δ = 7.49–7.34 (m, 5H), 4.21 (q, *J* = 7.2, 1H), 2.65 (s, 3H), 1.81 (d, *J* = 7.2 Hz, 3H), Spectral data matched those reported previously.[22]

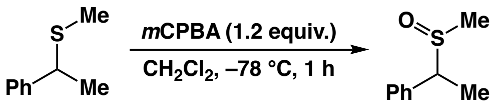

Sulfoxide **40** synthesis.

(1-(methylsulfinyl)ethyl)benzene (40). To a flame-dried 20 mL vial equipped with a stir bar was added methyl(1-phenylethyl) sulfane (**7**) (50 mg, 0.33 mmol, 1.0 equiv.) and CH_2Cl_2 (2.5 mL). The reaction vessel was cooled to –78 °C. *m*CPBA (68 mg, 0.4 mmol, 1.2 equiv.) was dissolved in CH_2Cl_2 (1.0 mL) in a separate vial and subsequently added to the reaction vessel dropwise over 5 min at –78 °C. The mixture was stirred for 20 min, then quenched with water (2.0 mL) and extracted with CH_2Cl_2 (2.0 mL x 3). The organic layers were combined, dried over Na_2SO_4, and concentrated under reduced pressure. The resultant mixture was purified with silica flash chromatography (1:3 EtOAc:hexane to pure methanol) to yield sulfoxide product **40** as a colorless crystal (32 mg, 60% yield, 1:5 mixture of diastereomers). **^{1}H NMR (500 MHz, $CDCl_3$)** Major, δ = 7.47–7.22 (m, 5H), 3.76 (q, *J* = 7.1, 1H), 2.30 (s, 3H), 1.75 (d, *J* = 7.2 Hz, 3H); Minor, δ = 7.47–7.22 (m, 5H), 3.86 (q, *J* = 7.2, 1H), 2.2 (s, 3H), 1.73 (d, *J* = 7.1 Hz, 3H). Spectral data matched those reported previously.[23]

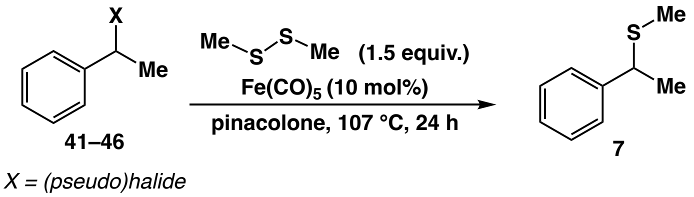

(Pseudo)halogen **41–46** screen.

(Pseudo)halogen (41–46) evaluation. To a flame-dried 4 mL dram vial equipped with a stir bar was added a $Fe(CO)_5$ (9.9 mg, 0.05 mmol, 6.8 uL, 10 mol%) in a glove box. The vial was taken out of the glove box and placed under N_2 in a fume-hood. To the vial was added pinacolone (1 mL), substrate (**41–46**) (0.5 mmol, 1.0 equiv.), and dimethyl disulfide (**2**) (70.7 mg, 0.75 mmol, 67.5 uL, 1.5 equiv.). The reaction vessel was sealed with a teflon screw cap and the mixture was stirred at 107 °C for 24 hours. The mixture was quenched with water (1.0 mL) and the aqueous layer was extracted with diethyl ether (3 x 1.0 mL). The organic layers were combined, dried over anhydrous sodium sulfate, and concentrated under reduced pressure. The residue was purified by silica flash chromatography (100% hexanes) to yield methyl thioether **7** as a colorless liquid. (R_f= 0.30, 100% hexanes).

Entry	Psuedohalide (X)	Isolated Yield %
1	OH	NR
2	F	< 5
3	Cl	76
4	I	92
5	$OP(O)(OPh)_2$	55
6	$OP(O)(Ph)_2$	46

Table S6. (Pseudo)halide evaluation.

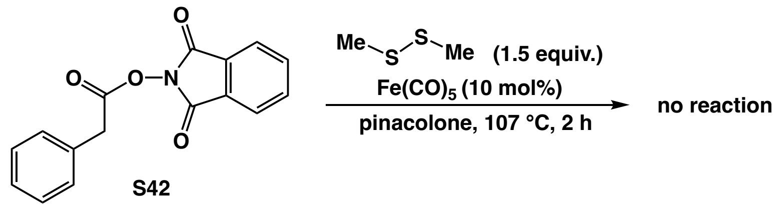

S42

Attempted use of redox ester **S42** as a substrate.

Redox ester S42 as a substrate. To a flame-dried 4 mL dram vial equipped with a stir bar was added a $Fe(CO)_5$ (9.9 mg, 0.05 mmol, 6.8 uL, 10 mol%) in a glove box. The vial was taken out of the glove box and placed under N_2 in a fume-hood. To the vial was added pinacolone (1 mL), redox ester substrate **S42** (0.5 mmol, 1.0 equiv.), and dimethyl disulfide (**2**) (70.7 mg, 0.75 mmol, 67.5 uL, 1.5 equiv.). The reaction vessel was sealed with a teflon screw cap and the mixture was stirred at 107 °C for 2 hours. The mixture was quenched with water (1.0 mL) and the aqueous layer was extracted with diethyl ether (3 x 1.0 mL). The organic layers were combined, dried over anhydrous sodium sulfate, and concentrated under reduced pressure. Only starting material was observed in the crude mixture and no trace amounts of thioether **21** were observed.

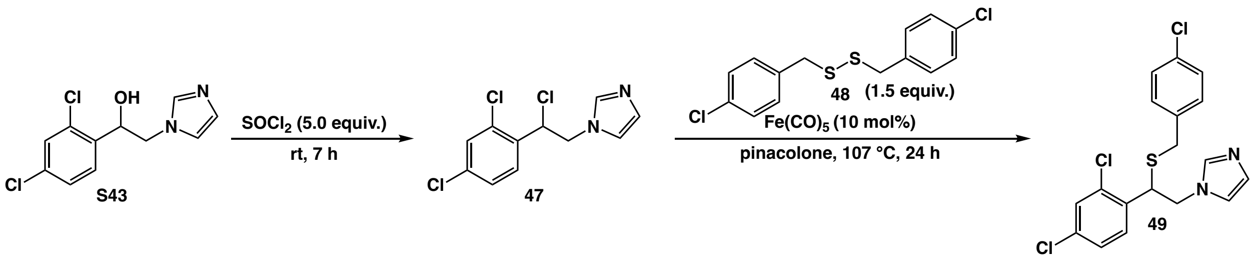

S43 47 49

Sulconazole (**49**) synthesis.

Sulconazole (49). To a flame-dried 20 mL vial equipped with a stir bar was added 1-(2,4-dichlorophenyl)-2-(1H-imidazol-1-yl)ethan-1-ol (**S43**) (771 mg, 3.0 mmol, 1.0 equiv.). Thionyl chloride (1.1 mL, 15.0 mmol, 5.0 equiv.) was added dropwise and the reaction was set to stir for 7 h at room temperature. The mixture was poured into an ice water mixture (30.0 mL). Solid sodium bicarbonate was then added to the mixture until the pH reached 7–8. A white precipitate formed and was filtered out using a cotton plug. The solid was dissolved using CH_2Cl_2 (20.0 mL),

the solution was dried over Na_2SO_4, and concentrated under reduced pressure. The resultant mixture was then purified by silica flash chromatography (1:1 hexanes:EtOAc to 100% EtOAc) to yield chloride **47** as a colorless liquid (827 mg, 99% yield). **^{1}H NMR (400 MHz, $CDCl_3$).** δ = 7.44–7.27 (m, 4H), 7.03 (br s, 1H), 6.86 (br s, 1H), 5.59 (t, J = 6.0 Hz, 1H), 4.49–4.34 (m, 2H).

To a flame-dried 4 mL dram vial equipped with a stir bar was added $Fe(CO)_5$ (5.0 mg, 0.025 mmol, 3.4 uL, 5 mol%) in a glove box. The vial was taken out of the glove box and placed under N_2 in a fume-hood. To the vial was added pinacolone (1.0 mL), chloride (**47**) (137.0 mg, 0.50 mmol, 1.0 equiv.), and disulfide **48** (235 mg, 0.75 mmol, 1.5 equiv.). The reaction vessel was sealed with a teflon screw cap and the mixture was stirred at 107 °C. The reaction mixture was cooled to room temperature after 12 hours, and an additional portion $Fe(CO)_5$ (5.0 mg, 0.025 mmol, 3.4 uL, 5 mol%) from the glove box was added via syringe. The reaction was stirred for an additional 12 h at 107 °C. The mixture was quenched with water (2.0 mL) and the aqueous layer was extracted with diethyl ether (3 x 2.0 mL). The organic layers were combined, dried over anhydrous sodium sulfate, and concentrated under reduced pressure. The residue was purified by silica flash chromatography (100% CH_2Cl_2 to 99:1 CH_2Cl_2:MeOH) to yield antifungal thioether **49** as a yellow liquid (75 mg, 38% yield). (R_f = 0.30, 99:1 CH_2Cl_2:MeOH). **^{1}H NMR (500 MHz, $CDCl_3$)** δ = 7.38–7.35 (m, 2H), 7.28–7.19 (m, 4H), 7.05 (d, J = 8.5 Hz, 2H), 7.00 (s, 1H), 6.72 (s, 1H), 4.45–4.28 (m, 1H), 4.20–4.18 (m, 2H), 3.50 (d, J = 13.6 Hz, 1H), 3.42 (d, J = 13.6 Hz, 1H). Spectral data matched those reported previously.[24]

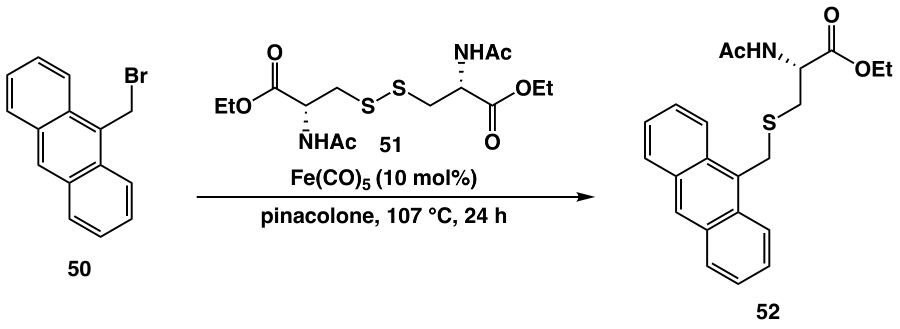

Bioconjugation of fluorophore **50** and cystine derivative **51**.

Ethyl N-acetyl-S-(anthracen-9-ylmethyl)-L-cysteinate (52). Method A: To a flame-dried 4 mL dram vial equipped with a stir bar was added $Fe(CO)_5$ (2.5 mg, 0.0125 mmol, 1.7 uL, 5 mol%) in a glove box. The vial was taken out of the glove box and placed under N_2 in a fume-hood. To the vial was added pinacolone (0.5 mL), 9-(bromomethyl)anthracene (**50**) (67.7 mg, 0.25 mmol, 1.0 equiv.), and cystine derivative **51** (171 mg, 0.375 mmol, 1.5 equiv.). The reaction vessel was sealed with a teflon screw cap and the mixture was stirred at 107 °C. The reaction mixture was cooled to room temperature after 12 hours, and an additional portion $Fe(CO)_5$ (2.5 mg, 0.0125 mmol, 1.7 uL, 5 mol%) from the glove box was added via syringe. The reaction was stirred for an additional 12 h at 107 °C. The mixture was quenched with water (1.0 mL) and the aqueous layer was extracted with diethyl ether (3 x 1.0 mL). The organic layers were combined, dried over anhydrous sodium sulfate, and concentrated under reduced pressure. The residue was purified by silica flash chromatography (9:1 to 2:5 hexanes:EtOAc) to yield thioether **52** as a yellow solid (35 mg, 73% yield).

Method B: To a flame-dried 4 mL dram vial equipped with a stir bar was added $Fe(CO)_5$ (2.5 mg, 0.025 mmol, 1.7 uL, 10 mol%) in a glove box. The vial was taken out of the glove box and placed under N_2 in a fume-hood. To the vial was added pinacolone (0.5 mL), 9-(bromomethyl)anthracene (**50**) (67.7 mg, 0.25 mmol, 1.0 equiv.), and cystine derivative **51** (171 mg, 0.375 mmol, 1.5 equiv.). The reaction vessel was sealed with a teflon screw cap and the mixture was stirred at 40 °C for 9 hours. The mixture was quenched with water (1.0 mL) and the aqueous layer was extracted with diethyl ether (3 x 1.0 mL). The organic layers were combined, dried over anhydrous sodium sulfate, and concentrated under reduced pressure. The residue was purified by silica flash chromatography (9:1 to 2:5 hexanes:EtOAc) to yield thioether **52** as a yellow solid (26 mg, 54%

yield). (R_f = 0.40, hexanes:EtOAc 1:1). **Mp:** 139 – 140 °C. **^{1}H NMR (500 MHz, CDCl$_3$).** δ = 8.41 (s, 1H), 8.29 (d, *J* = 8.9 Hz, 2H), 8.01 (d, *J* = 8.4 Hz, 2H), 7.59–7.56 (m, 2H), 7.49–7.46 (m, 2H), 6.26 (d, *J* = 7.8 Hz, 1H), 4.92 (q, *J* = 5.4 Hz, 1H), 4.78 (s, 2H), 4.24–4.15 (m, 2H), 3.16 (qd, *J* = 13.9, 5.1 Hz, 2H), 1.99 (s, 3H), 1.24 (t, *J* = 7.2 Hz, 3H). **^{13}C NMR (125 MHz, CDCl$_3$).** δ = 171.1, 170.0, 131.6, 130.1, 129.4, 128.4, 127.9, 126.5, 125.2, 124.2, 62.1, 52.1, 35.6, 29.8, 23.3, 14.2. **IR (solution, acetone).** 2896, 2824, 2364, 1708, 1368, 1032 cm^{-1}. **HRMS-QTOF-ESI** (m/z) [M + Ag]$^+$ calcd for $C_{22}H_{23}AgNO_3S^+$, 488.0450; found 488.0458.

12. Mechanistic analysis procedures.

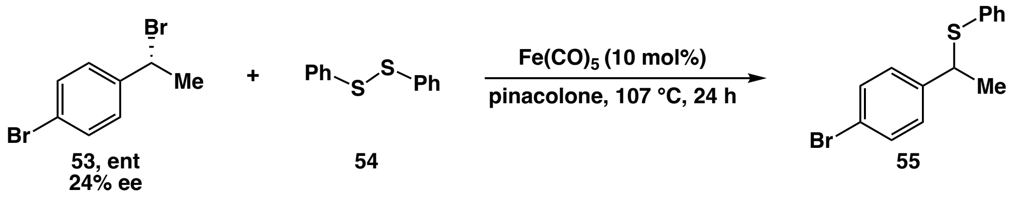

Examination of reaction stereospecificity.

To a flame-dried 4 mL dram vial equipped with a stir bar was added an iron catalyst (6.8 uL, 0.05 mmol, 10 mol%) in a glove box. The vial was taken out of the glove box and placed under N_2 in a fume-hood. To the vial was added solvent (1.0 mL), 1-bromo-4-(1-bromoethyl)benzene (**53, ent**) (132.0 mg, 0.5 mmol, 1.0 equiv., 24% ee), and disulfide (164 mg, 0.75 mmol, 1.5 equiv.). The reaction vessel was sealed with a teflon screw cap and the mixture was stirred at 107 °C for 24 hours. The mixture was quenched with water (1.0 mL) and the aqueous layer was extracted with diethyl ether (3 x 1.0 mL). The organic layers were combined, dried over anhydrous sodium sulfate, and concentrated under reduced pressure. The resultant mixture was purified by silica flash chromatography (100% hexanes) to yield thioether **55** as a colorless oil (120 mg, 82% yield, 0% ee). Chiral SFC: 250 mm ChiralPak IJ–3, 10% MeOH, 3.0 mL/min, λ = 230 nm, 35 °C, nozzle pressure = 140 bar CO_2, tR1 = 4.22 min, tR2 = 4.73 min. **^{1}H NMR (600 MHz, $CDCl_3$).** δ = 7.38 (d, J = 8.4 Hz, 2H), 7.28–7.19 (m, 5H), 7.15 (d, J = 8.5 Hz, 2H), 4.28 (q, J = 7.0 Hz, 1H), 1.60 (d, J = 7.1 Hz, 3H). Spectral data matched those reported previously.[25]

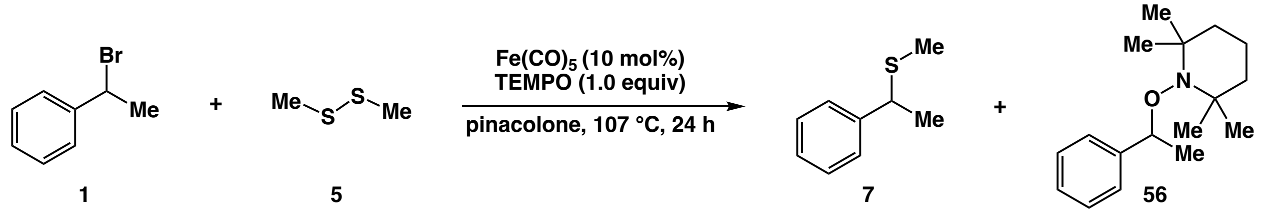

Spin trap experiment.

To a flame-dried 4 mL dram vial equipped with a stir bar was added an iron catalyst (6.8 uL, 0.05 mmol, 10 mol%) in a glove box. The vial was taken out of the glove box and placed under N_2 in a

fume-hood. To the vial was added solvent (1.0 mL), (1-bromoethyl)benzene (**1**) (92.5 mg, 0.5 mmol, 1.0 equiv.), disulfide (70.5 mg, 0.75 mmol, 1.5 equiv.), and TEMPO (78.1 mg, 0.5 mmol, 1.0 equiv). The reaction vessel was sealed with a teflon screw cap and the mixture was stirred at 107 °C for 24 hours. The mixture was quenched with water (1.0 mL) and the aqueous layer was extracted with diethyl ether (3 x 1.0 mL). The organic layers were combined, dried over anhydrous sodium sulfate, and concentrated under reduced pressure. The resultant mixture was purified by silica flash chromatography (100% hexanes) to yield thioether **7** (23 mg, 30% yield) and TEMPO adduct **56** (85 mg, 65% yield). Thioether **7** is tabulated above. TEMPO adduct **56**: **^{1}H NMR (400 MHz, $CDCl_3$).** δ = 7.35-7.21 (m, 5H), 4.85 (q, J= 6.4 Hz, 1H), 1.46 (d, J= 6.4 Hz, 3H), 1.41-1.25 (m, 6H), 1.29 (s, 3H), 1.16 (s, 3H), 1.02 (s, 3H), 0.65 (s, 3H). Spectral data matched those reported previously.[26]

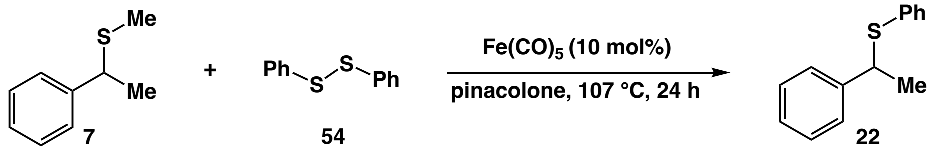

Reaction-active thioether experiment.

To a flame-dried 4 mL dram vial equipped with a stir bar was added an iron catalyst (6.8 uL, 0.05 mmol, 10 mol%) in a glove box. The vial was taken out of the glove box and placed under N_2 in a fume-hood. To the vial was added solvent (1.0 mL), thioether (**7**) (76 mg, 0.5 mmol, 1.0 equiv.), and disulfide (164 mg, 0.75 mmol, 1.5 equiv.). The reaction vessel was sealed with a teflon screw cap and the mixture was stirred at 107 °C for 24 hours. The mixture was quenched with water (1.0 mL) and the aqueous layer was extracted with diethyl ether (3 x 1.0 mL). The organic layers were combined, dried over anhydrous sodium sulfate, and concentrated under reduced pressure. The resultant mixture was analyzed by NMR internal standard using 1,3,5-trimethoxybenzene. Thioether **22** was observed in 75% yield. Thioether **22** is tabulated above.

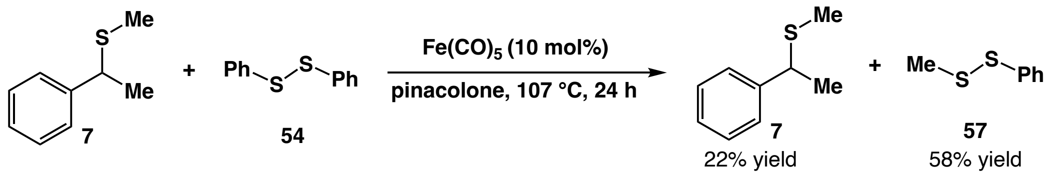

Sulfur unit tracking experiment.

To a flame-dried 4 mL dram vial equipped with a stir bar was added an iron catalyst (6.8 uL, 0.05 mmol, 10 mol%) in a glove box. The vial was taken out of the glove box and placed under N_2 in a fume-hood. To the vial was added solvent (1.0 mL), thioether (**7**) (76 mg, 0.5 mmol, 1.0 equiv.), and disulfide (164 mg, 0.75 mmol, 1.5 equiv.). The reaction vessel was sealed with a teflon screw cap and the mixture was stirred at 107 °C for 24 hours. The mixture was quenched with water (1.0 mL) and the aqueous layer was extracted with diethyl ether (3 x 1.0 mL). The organic layers were combined, dried over anhydrous sodium sulfate, and concentrated under reduced pressure. The resultant mixture was analyzed by NMR internal standard using 1,3,5-trimethoxybenzene. Thioether **7** was observed in 22% yield and mixed disulfide **57** was observed in 58%, accounting for 80% of sulfur unit input. Thioether **7** is tabulated above. Mixed disulfide **57**: **^{1}H NMR (400 MHz, $CDCl_3$).** δ = 7.54 (d, J = 8.0 Hz, 2H), 7.34 (t, J = 8.0 Hz, 2H), 7.24 (t, J = 8.0 Hz, 1H), 2.45 (s, 3H). Spectral data matched those reported previously.[27]

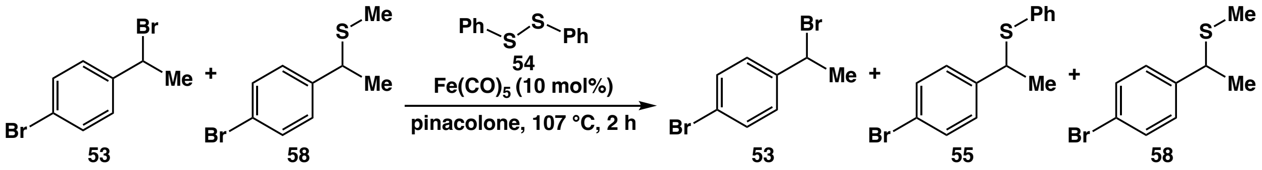

Bromide vs thioether competition experiment.

To a flame-dried 4 mL dram vial equipped with a stir bar was added an iron catalyst (6.8 uL, 0.05 mmol, 10 mol%) in a glove box. The vial was taken out of the glove box and placed under N_2 in a fume-hood. To the vial was added solvent (1.0 mL), bromide (**53**) (0.25 mmol, 1.0 equiv.), thioether **58** (0.25 mmol, 1.0 equiv.), and disulfide (164 mg, 0.75 mmol, 1.5 equiv. to combined substrate mmol). The reaction vessel was sealed with a teflon screw cap and the mixture was stirred

at 107 °C for 2 hours. The mixture was quenched with water (1.0 mL) and the aqueous layer was extracted with diethyl ether (3 x 1.0 mL). The organic layers were combined, dried over anhydrous sodium sulfate, and concentrated under reduced pressure. The resultant mixture was analyzed by NMR internal standard using 1,3,5-trimethoxybenzene. Bromide **53**, thioether **55**, and thioether **58** were observed at 20%, 66%, and 14% yield, respectively. Bromide **53**, thioether **55**, and thioether **58** are tabulated above.

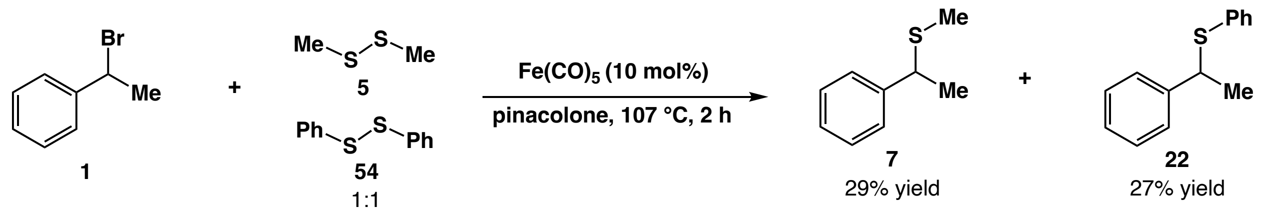

Alkyl vs aryl disulfide competition experiment.

To a flame-dried 4 mL dram vial equipped with a stir bar was added an iron catalyst (6.8 uL, 0.05 mmol, 10 mol%) in a glove box. The vial was taken out of the glove box and placed under N_2 in a fume-hood. To the vial was added solvent (1.0 mL), bromide (**1**) (92.5 mg, 0.5 mmol, 1.0 equiv.), disulfide **5** (35 mg, 0.38 mmol, 0.75 equiv.), and disulfide **54** (82 mg, 0.38 mmol, 0.75 equiv.). The reaction vessel was sealed with a teflon screw cap and the mixture was stirred at 107 °C for 2 hours. The mixture was quenched with water (1.0 mL) and the aqueous layer was extracted with diethyl ether (3 x 1.0 mL). The organic layers were combined, dried over anhydrous sodium sulfate, and concentrated under reduced pressure. The resultant mixture was analyzed by NMR internal standard using 1,3,5-trimethoxybenzene. Thioether **7** and thioether **22** were observed at 29% and 27% yield, respectively. Thioether **7** and thioether **22** are tabulated above.

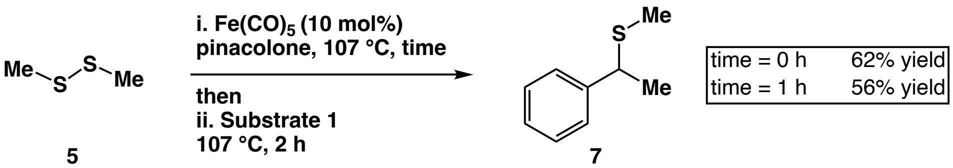

5 7

Precatalyst disulfide incubation effects.

To a flame-dried 4 mL dram vial equipped with a stir bar was added an iron catalyst (6.8 uL, 0.05 mmol, 10 mol%) in a glove box. The vial was taken out of the glove box and placed under N_2 in a fume-hood. To the vial was added solvent (1.0 mL), and disulfide **5** (70.5 mg, 0.75 mmol, 1.5 equiv.). The reaction vessel was sealed with a teflon screw cap and the mixture was stirred at 107 °C for 1 hour. The vial was removed from heat and bromide **1** (92.5 mg, 0.5 mmol, 1.0 equiv.) was added to the reaction mixture. The reaction was allowed to stir for an additional 2 hours. The mixture was quenched with water (1.0 mL) and the aqueous layer was extracted with diethyl ether (3 x 1.0 mL). The organic layers were combined, dried over anhydrous sodium sulfate, and concentrated under reduced pressure. The resultant mixture was analyzed by NMR internal standard using 1,3,5-trimethoxybenzene. Thioether **7** was observed at 56% yield.

Thioether **7** was observed at 62% yield in the control experiment stirred for 2 hours under standard reaction conditions. All reagents were added sequentially without allowing iron pentacarbonyl to stir with disulfide.

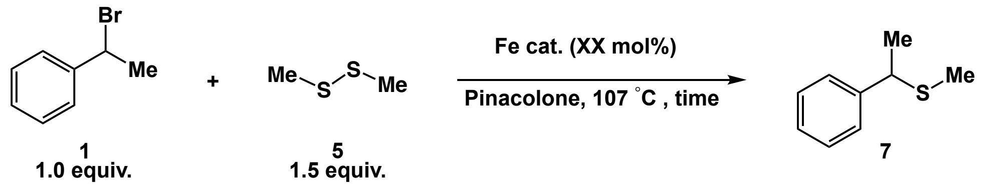

entry	Fe cat.	Fe loading	incubation partner	incubation time	reaction time	result
1	$FeCl_2$	10 mol%	none	none	2 h	53%
2	$Fe(CO)_5$	10 mol%	none	none	2 h	65%
3	$FeCl_2$	30 mol%	none	none	10 min	69%
4	$Fe(CO)_5$	30 mol%	none	none	10 min	86%
5	$FeCl_2$	10 mol%	disulfide	1 h	2 h	47%
6	$Fe(CO)_5$	10 mol%	disulfide	1 h	2 h	57%
7	$FeCl_2$	30 mol%	disulfide	1 h	10 min	60%
8	$Fe(CO)_5$	30 mol%	disulfide	1 h	10 min	87%
9	$FeCl_2$	10 mol%	bromide	1 h	2 h	23%
10	$Fe(CO)_5$	10 mol%	bromide	1 h	2 h	26%
11	$FeCl_2$	30 mol%	bromide	1 h	10 min	11%
12	$Fe(CO)_5$	30 mol%	bromide	1 h	10 min	16%

Table S7. Additional disulfide and bromide incubation effects with $FeCl_2$ and $Fe(CO)_5$.

We conducted additional incubation experiments to narrow the mechanistic possibilities further. Incubating either $FeCl_2$ or $Fe(CO)_5$ with disulfide substrate, regardless of catalyst loading, resulted in similar thioether production as control experiments (entries 1–8). Additionally, incubating either $FeCl_2$ or $Fe(CO)_5$ with bromide substrate, regardless of catalyst loading, resulted in lower thioether production (entries 9–12). The data suggests that both $FeCl_2$ and $Fe(CO)_5$ are first interacting with the bromide substrate.

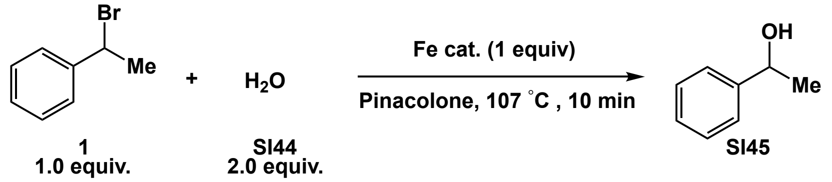

entry	Fe cat.	result
1	$FeCl_2$	20%
2	$Fe(CO)_5$	93%

Table S8. Trapping purported oxidative addition intermediates.

Upon realizing the results described in Table S7, we suspected that an oxidative cleavage was taking place between Fe and bromide **1**. When running the reaction with one equivalent of $FeCl_2$ or $Fe(CO)_5$ and with water in place of disulfide, alcohol **SI45** was produced in 20% and 93% yield for $FeCl_2$ and $Fe(CO)_5$, respectively. These preliminary results are consistent with a more efficient

oxidative cleavage event with $Fe(CO)_5$ in comparison to $FeCl_2$, rendering $Fe(CO)_5$ as the better catalyst.

A full experimental and computational investigation is required to fully understand each elementary step and the exact mechanistic sequence.

13. ^{1}H, ^{13}C, and ^{19}F NMR data.

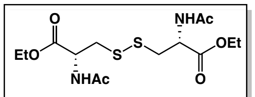

51

Title	PROTON_01
Origin	Varian
Instrument	vnmrs
Solvent	cdcl3
Temperature	25.0
Pulse Sequence	s2pul
Experiment	1D
Probe	AutoX_DB_MR0808W005
Number of Scans	32
Receiver Gain	48
Relaxation Delay	1.0000
Pulse Width	4.1500
Acquisition Time	1.7039
Acquisition Date	2023-07-23T12:55:58
Modification Date	2023-07-23T13:24:24
Spectrometer Frequency	599.79
Spectral Width	9615.4
Lowest Frequency	-1199.9
Nucleus	1H
Acquired Size	16384
Spectral Size	65536
Digital Resolution	0.15

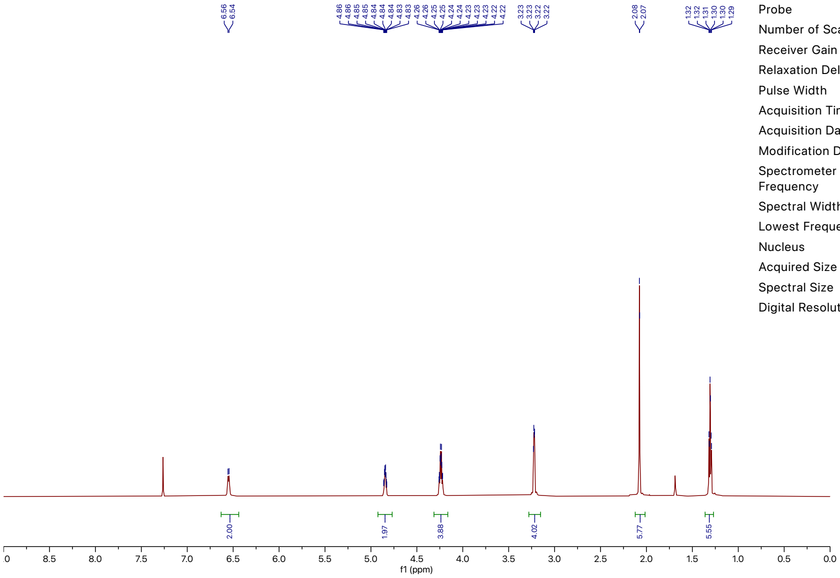

Fig. S1. ^{1}H NMR spectrum of disulfide **51**.

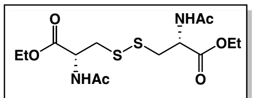

51

Title	CARBON_01
Origin	Varian
Instrument	vnmrs
Solvent	cdcl3
Temperature	25.0
Pulse Sequence	s2pul
Experiment	1D
Probe	AutoX_DB_MR0808W005
Number of Scans	5000
Receiver Gain	30
Relaxation Delay	1.0000
Pulse Width	4.5000
Acquisition Time	0.8651
Acquisition Date	2023-07-23T15:39:53
Modification Date	2023-07-23T16:32:47
Spectrometer Frequency	150.83
Spectral Width	37878.8
Lowest Frequency	-2316.0
Nucleus	13C
Acquired Size	32768
Spectral Size	65536
Digital Resolution	0.58

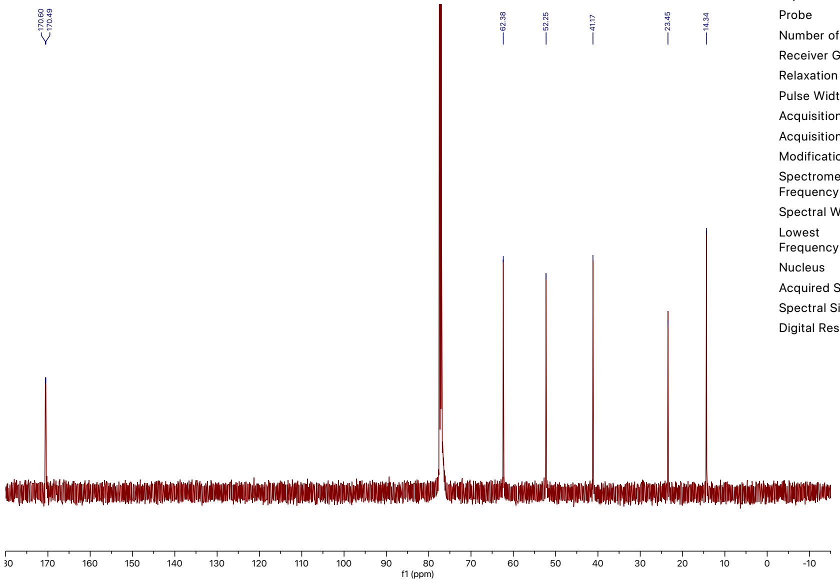

Fig. S2. ^{13}C NMR spectrum of disulfide **51**.

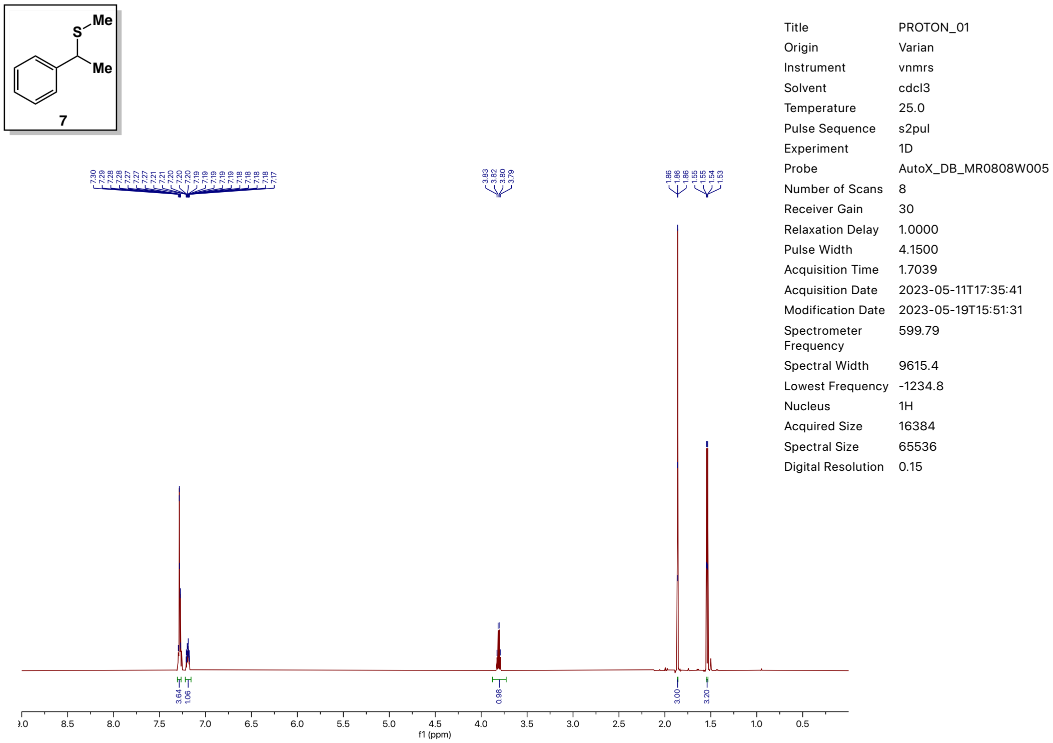

Fig. S3. ^{1}H NMR spectrum of thioether **7**.

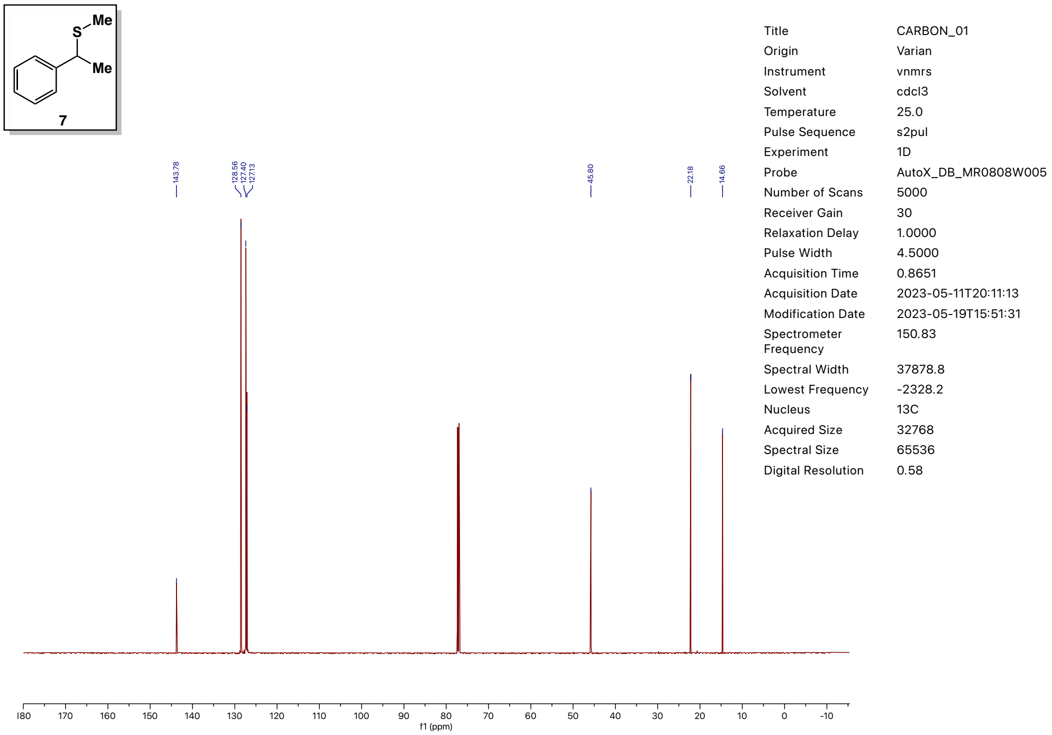

Fig. S4. ^{13}C NMR spectrum of thioether **7**.

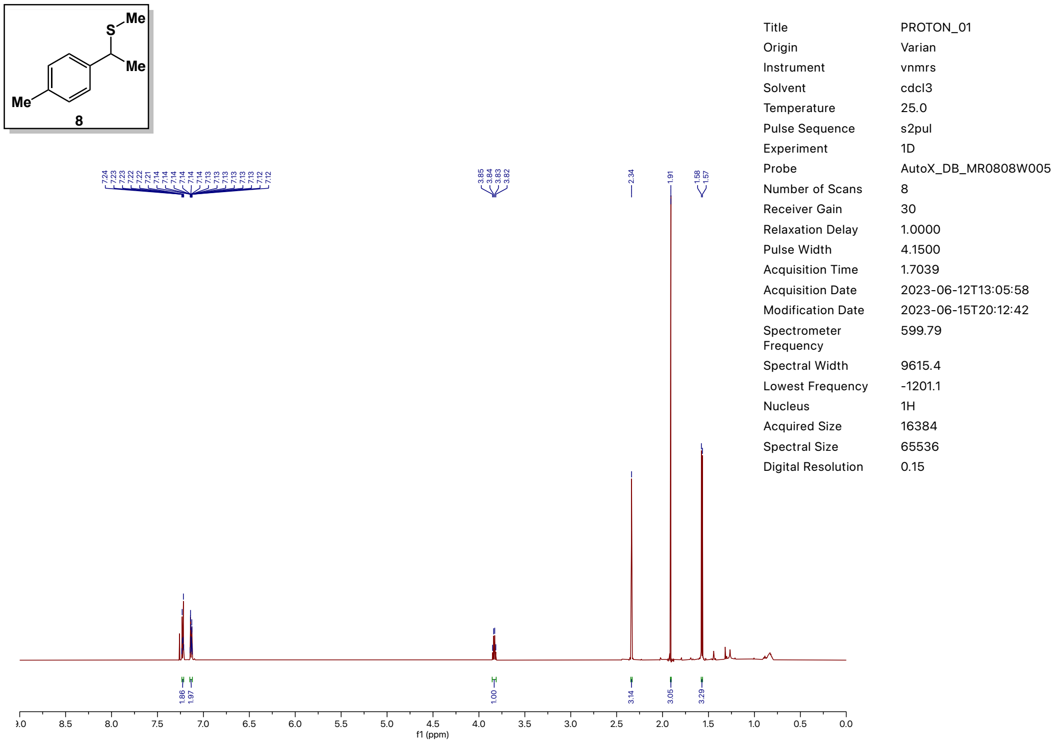

Fig. S5. ^{1}H NMR spectrum of thioether **8**.

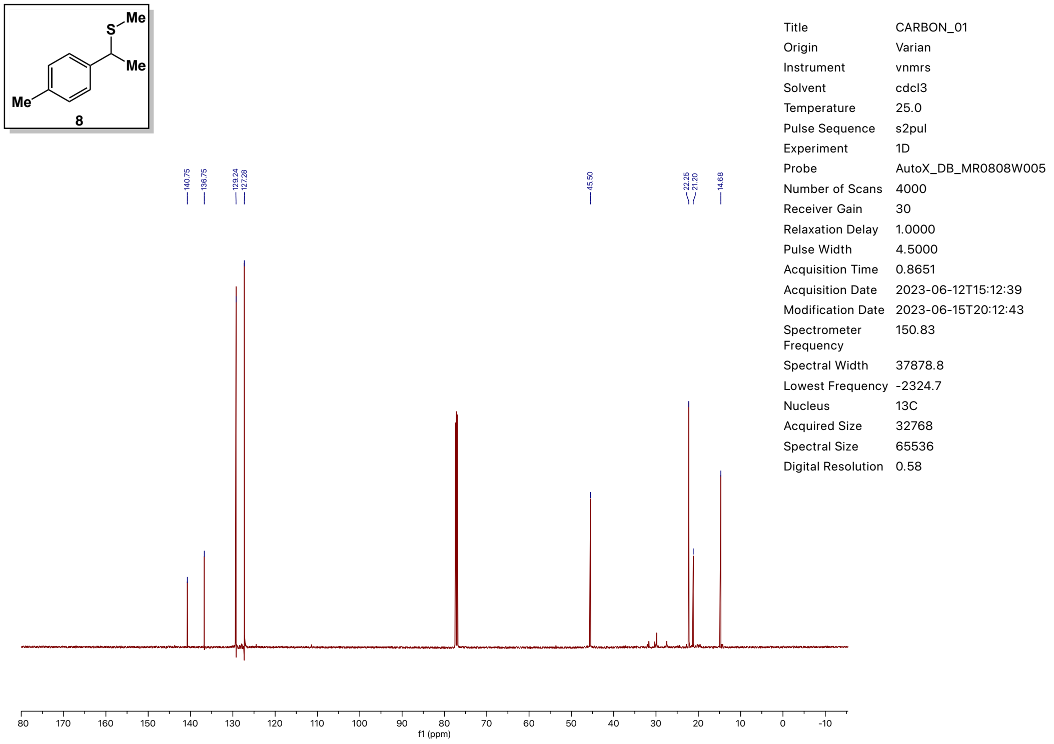

Fig. S6. ^{13}C NMR spectrum of thioether **8**.

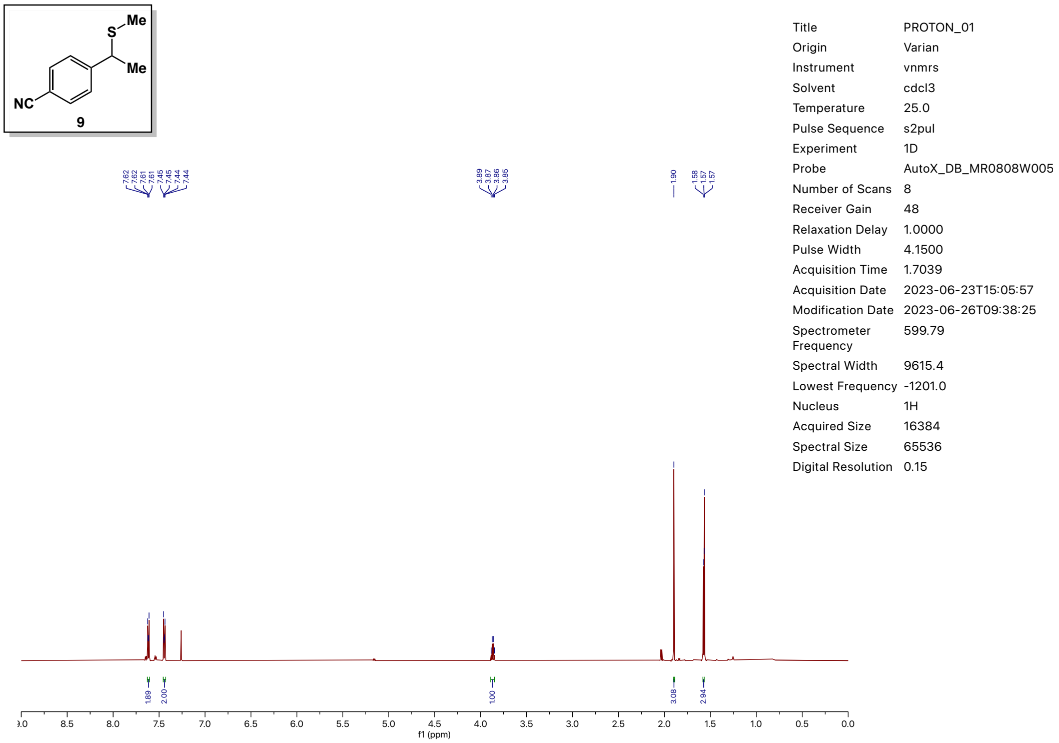

Fig. S7. ^{1}H NMR spectrum of thioether **9**.

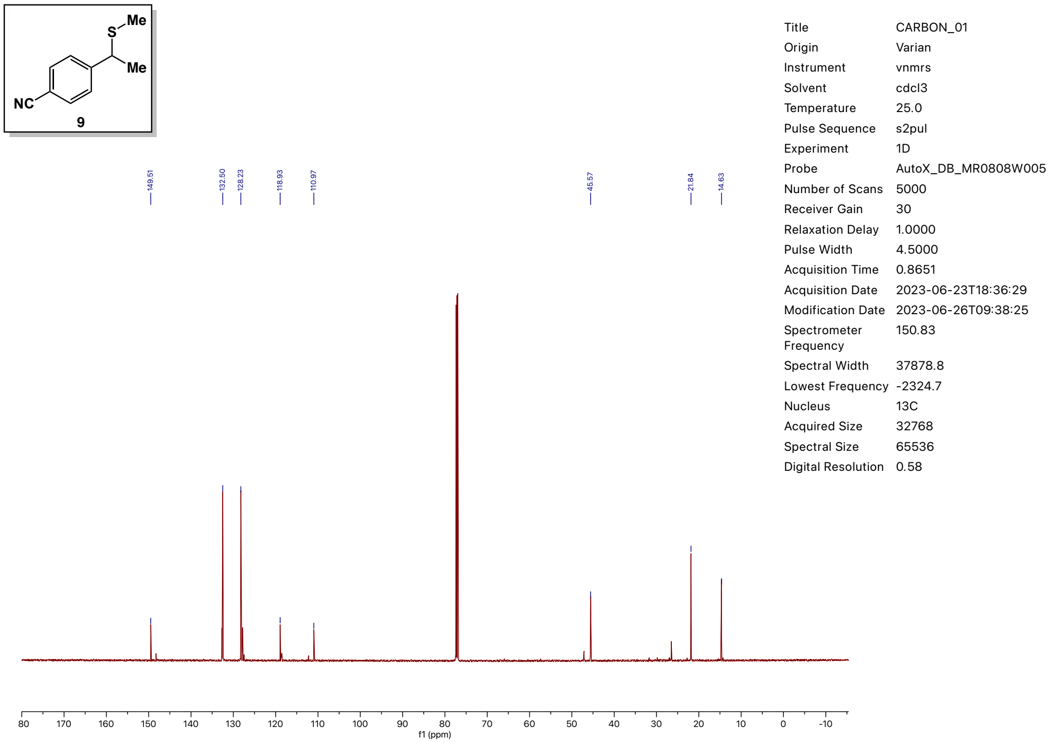

Fig. S8. ^{13}C NMR spectrum of thioether **9**.

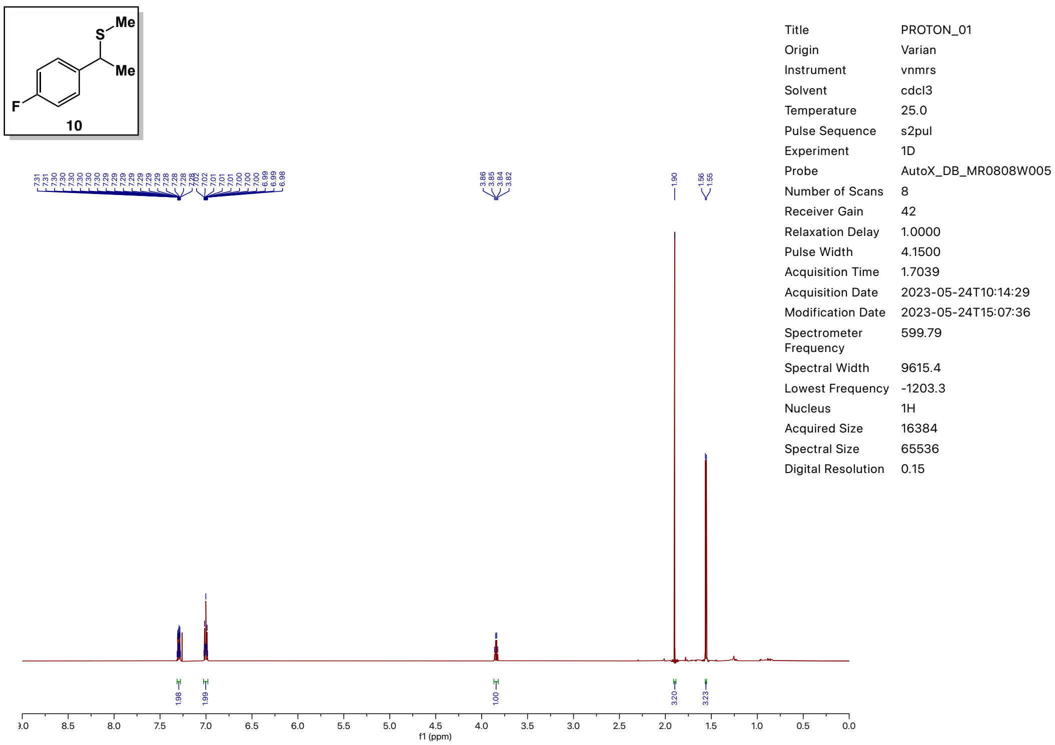

Fig. S9. ^{1}H NMR spectrum of thioether **10**.

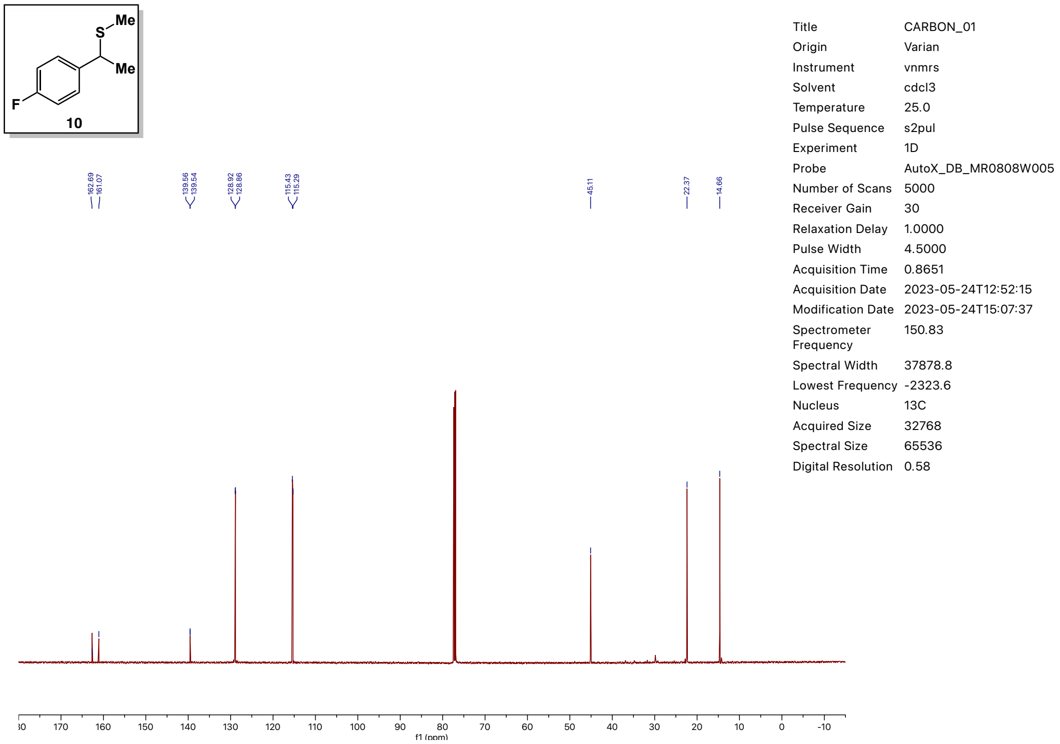

Fig. S10. ^{13}C NMR spectrum of thioether **10**.

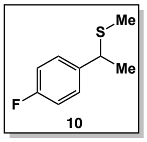

Title	FLUORINE_02
Origin	Varian
Instrument	vnmrs
Solvent	cdcl3
Temperature	25.0
Pulse Sequence	s2pul
Experiment	1D
Probe	AutoX_DB_MR0808W005
Number of Scans	64
Receiver Gain	50
Relaxation Delay	1.0000
Pulse Width	4.8667
Acquisition Time	0.9961
Acquisition Date	2023-07-20T13:06:11
Modification Date	2023-07-20T13:27:46
Spectrometer Frequency	564.32
Spectral Width	131578.9
Lowest Frequency	-113760.7
Nucleus	19F
Acquired Size	131072
Spectral Size	262144
Digital Resolution	0.50

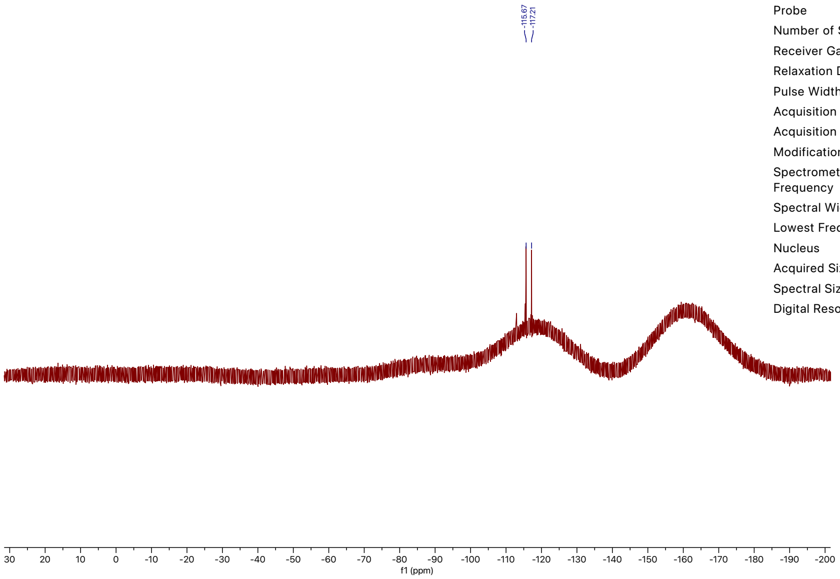

Fig. S11. ^{19}F NMR spectrum of thioether **10**.

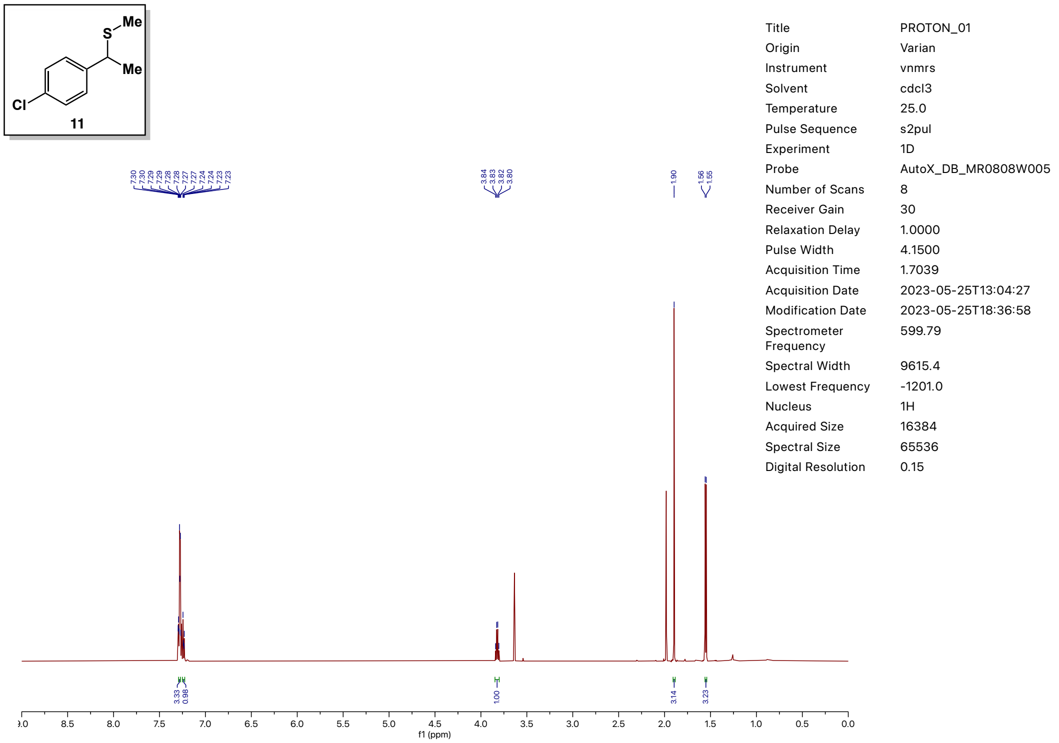

Fig. S12. ^{1}H NMR spectrum of thioether **11**.

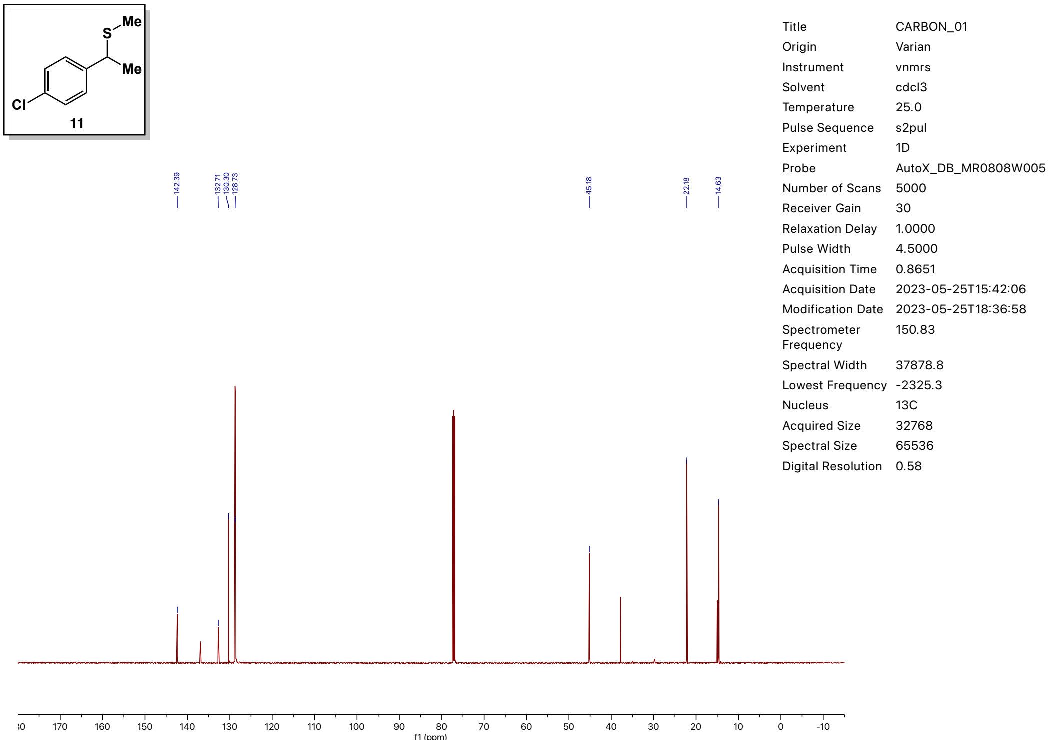

Fig. S13. ^{13}C NMR spectrum of thioether **11**.

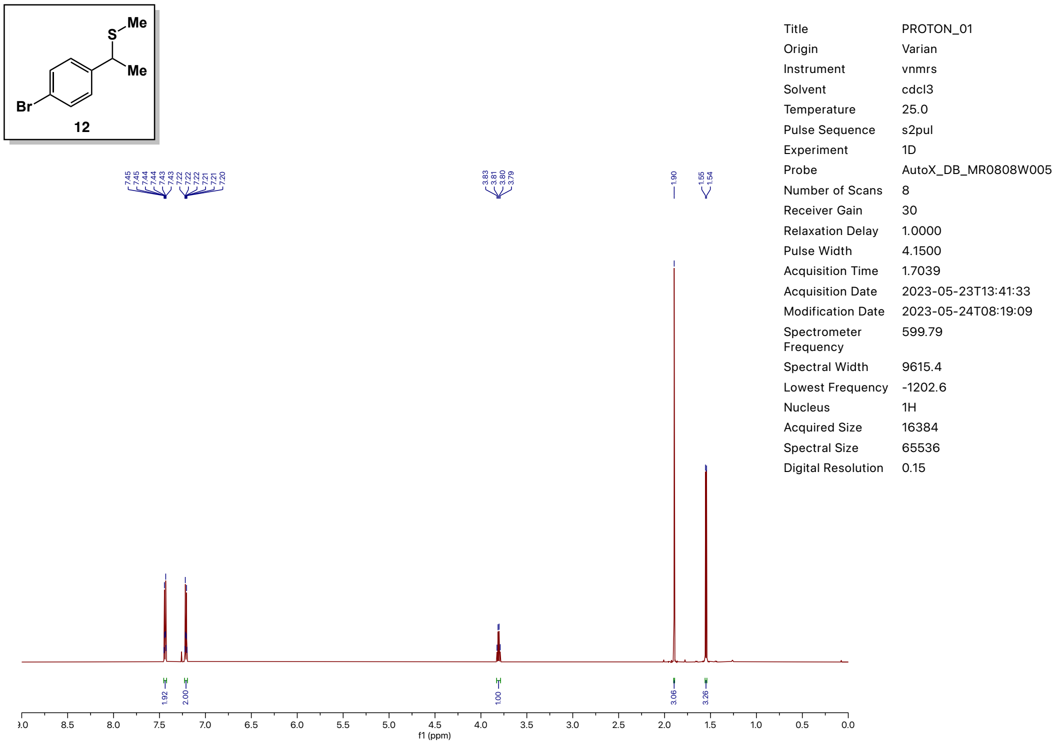

Fig. S14. ^{1}H NMR spectrum of thioether **12**.

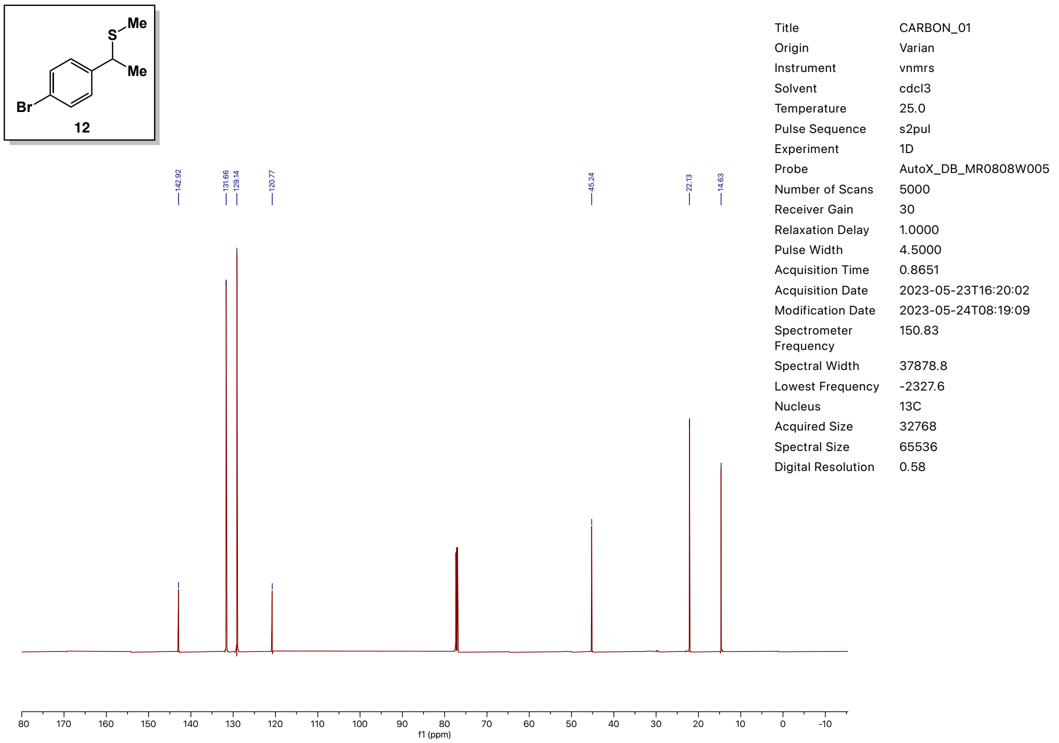

Fig. S15. ^{13}C NMR spectrum of thioether **12**.

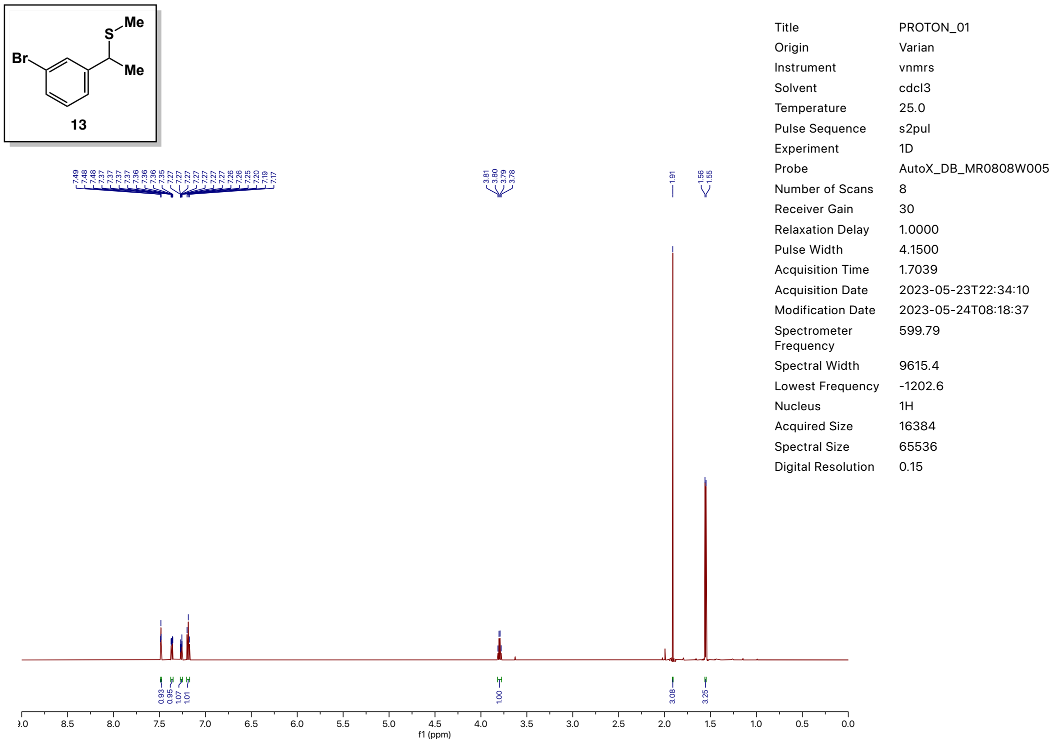

Fig. S16. ^{1}H NMR spectrum of thioether **13**.

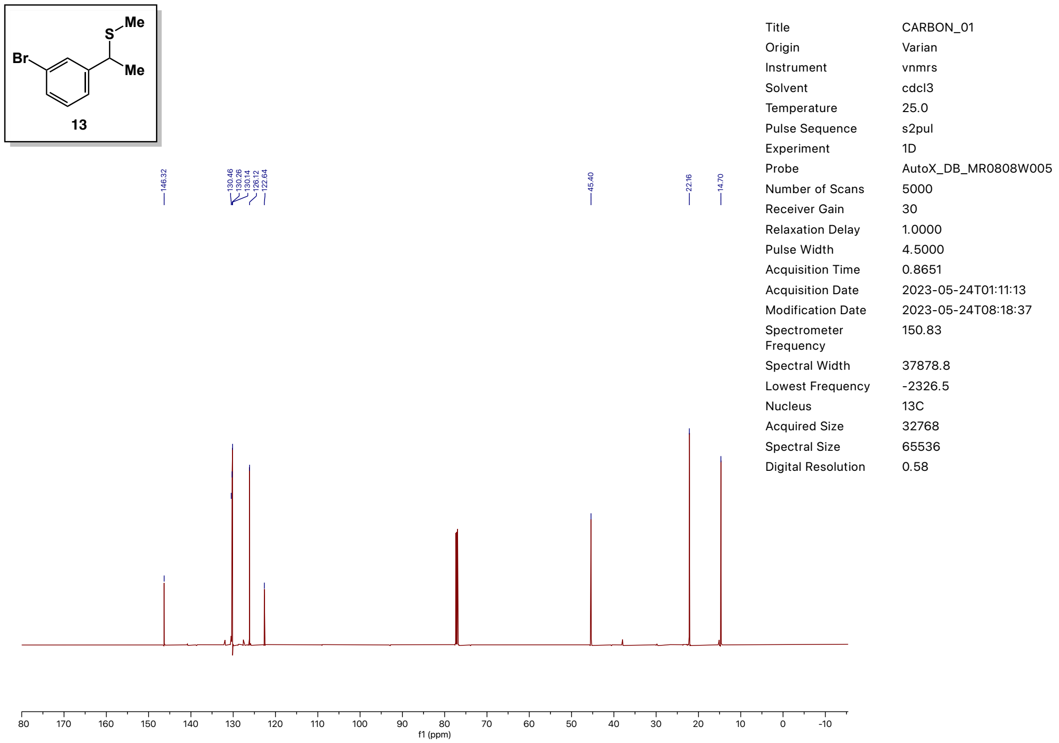

Fig. S17. ^{13}C NMR spectrum of thioether **13**.

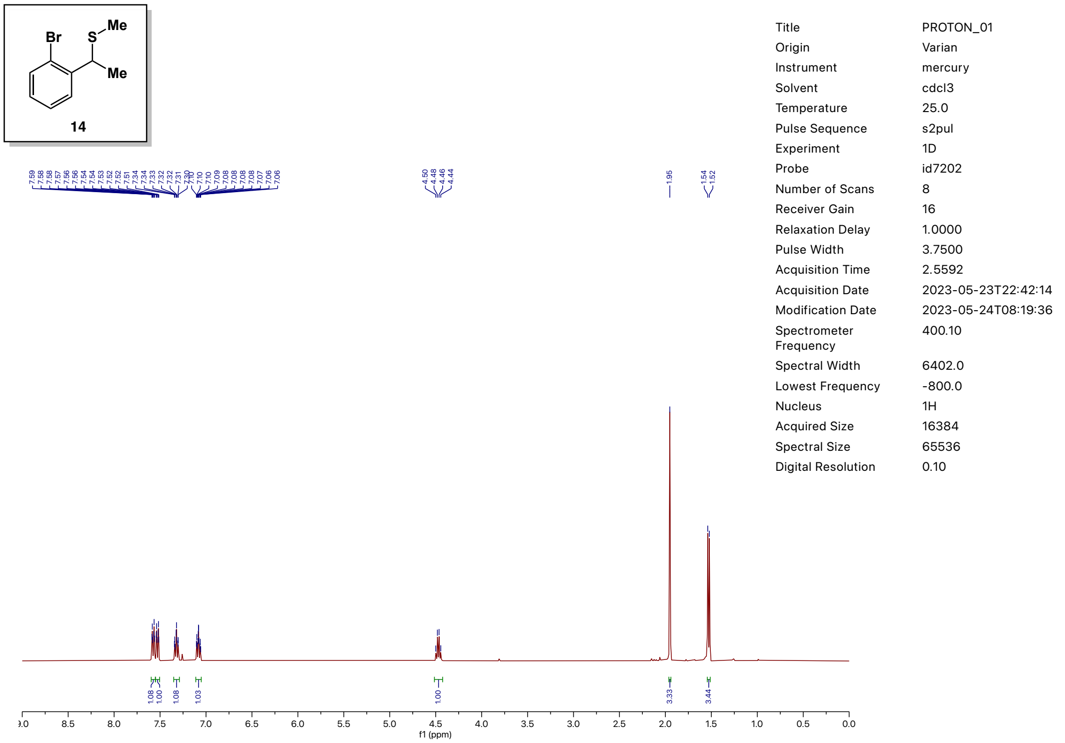

Fig. S18. ^{1}H NMR spectrum of thioether **14**.

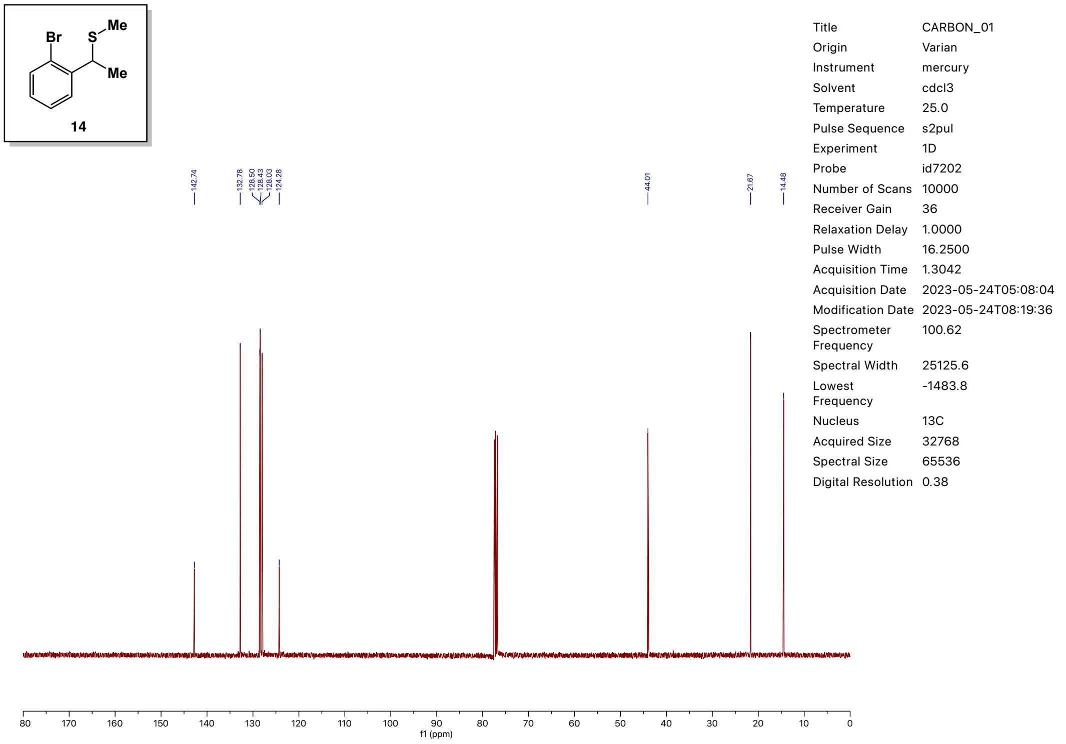

Fig. S19. ^{13}C NMR spectrum of thioether **14**.

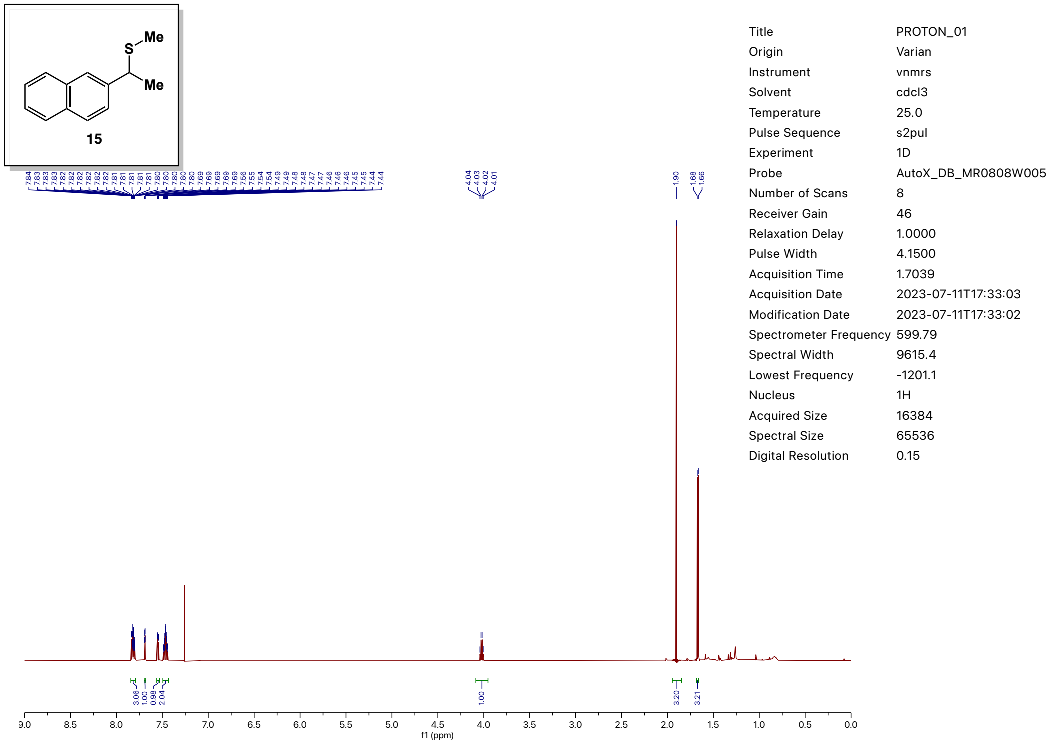

Fig. S20. ^{1}H NMR spectrum of thioether **15**.

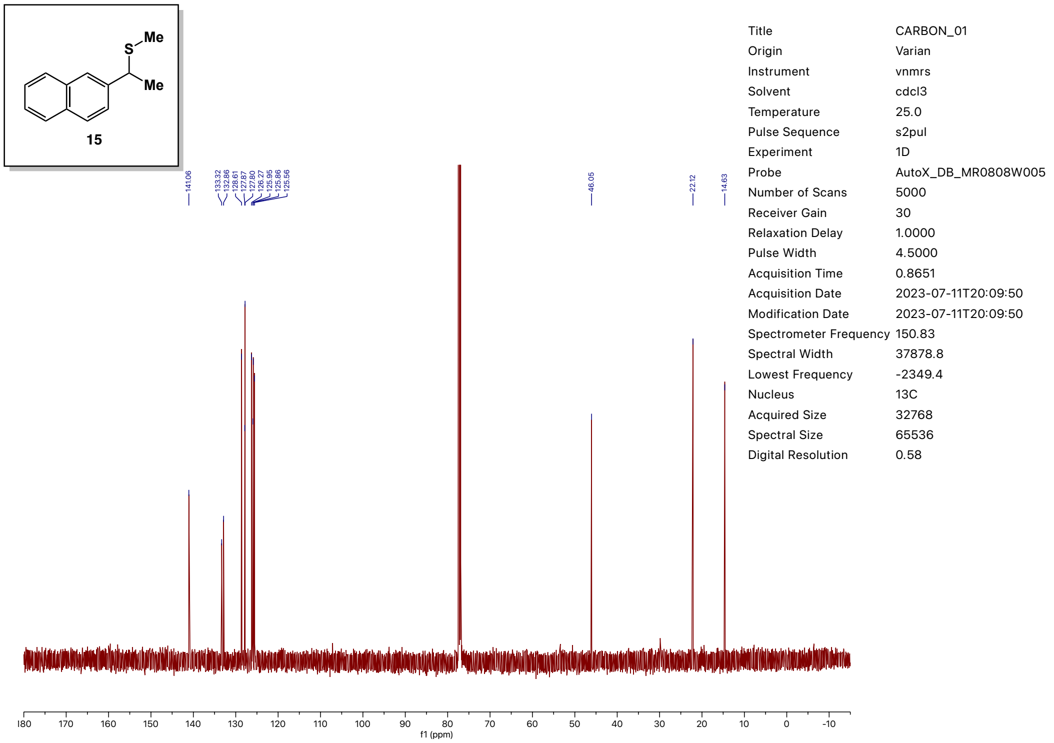

Fig. S21. ^{13}C NMR spectrum of thioether **15**.

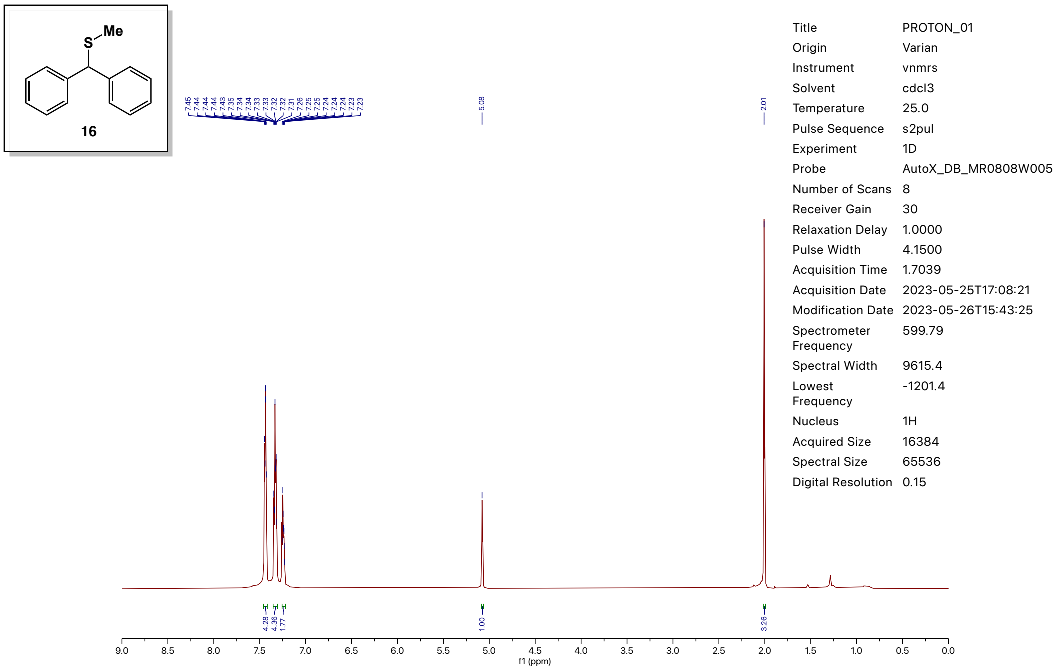

Fig. S22. ^{1}H NMR spectrum of thioether **16**.

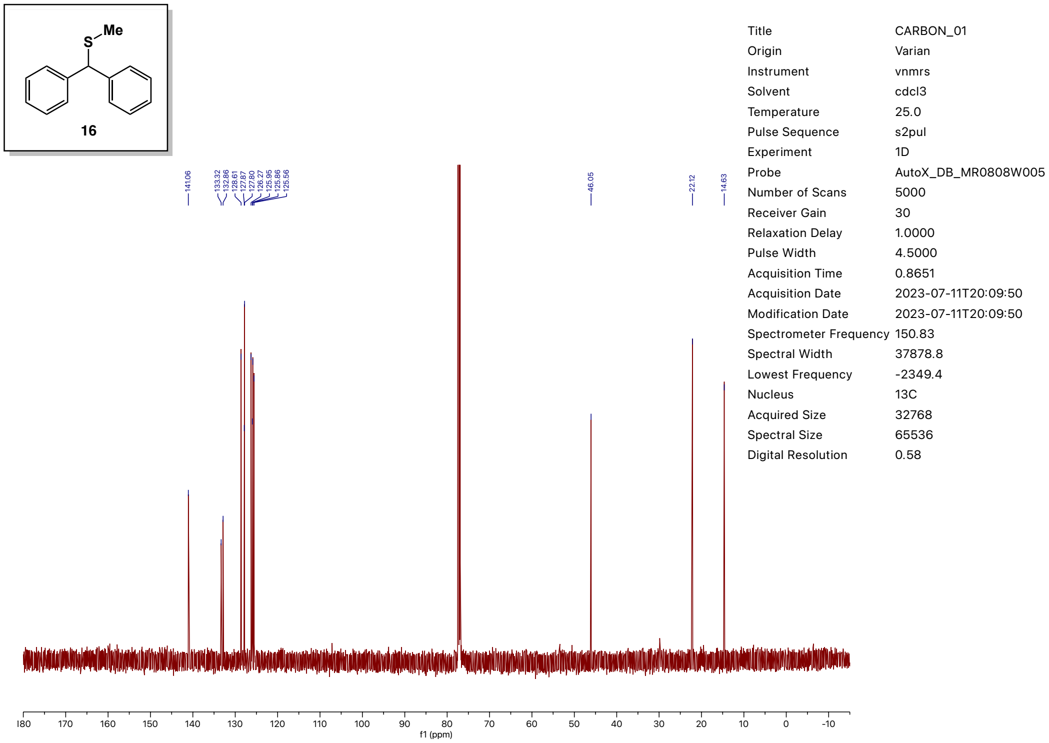

Fig. S23. ^{13}C NMR spectrum of thioether **16**.

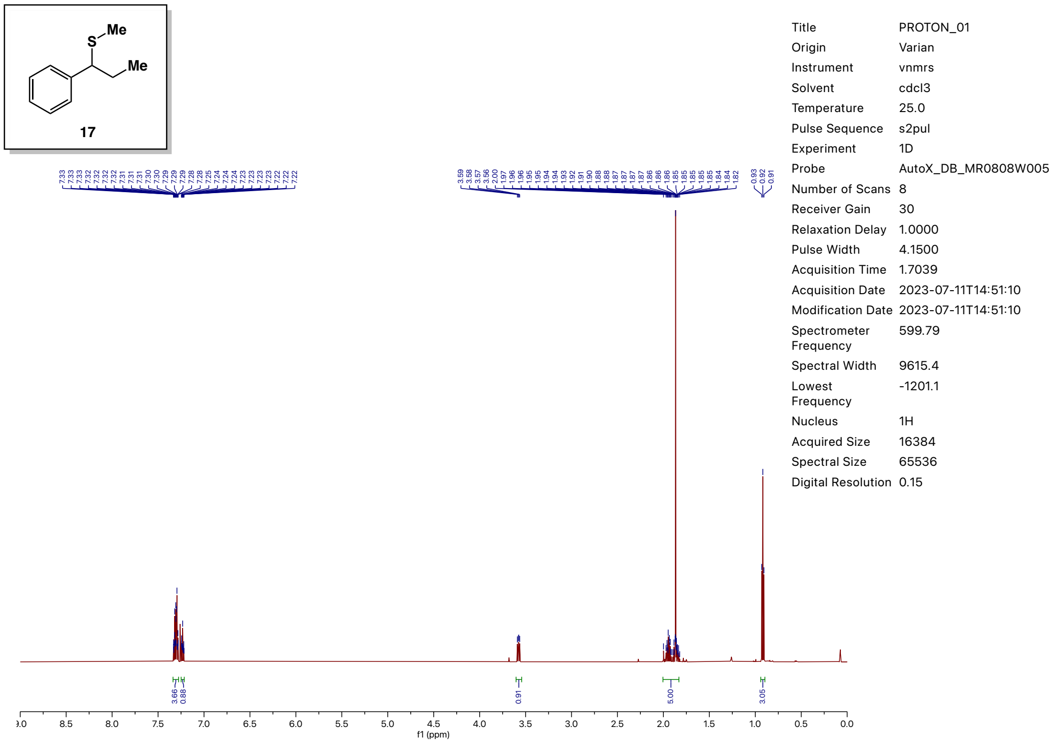

Fig. S24. ^{1}H NMR spectrum of thioether **17**.

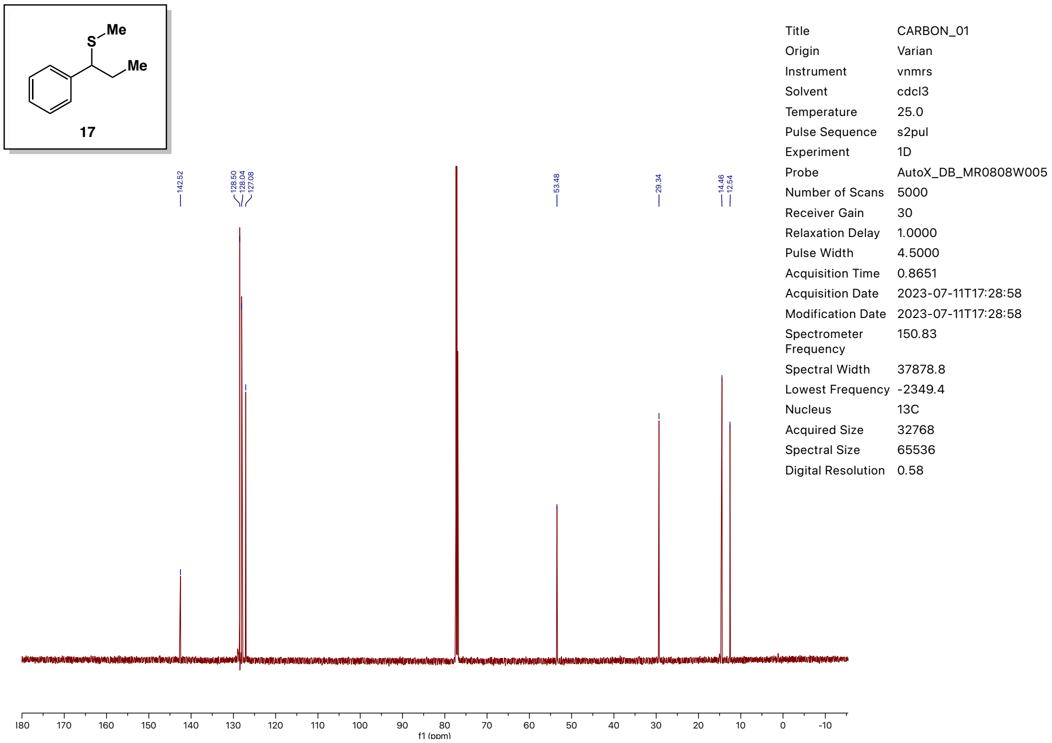

Fig. S25. ^{13}C NMR spectrum of thioether **17**.

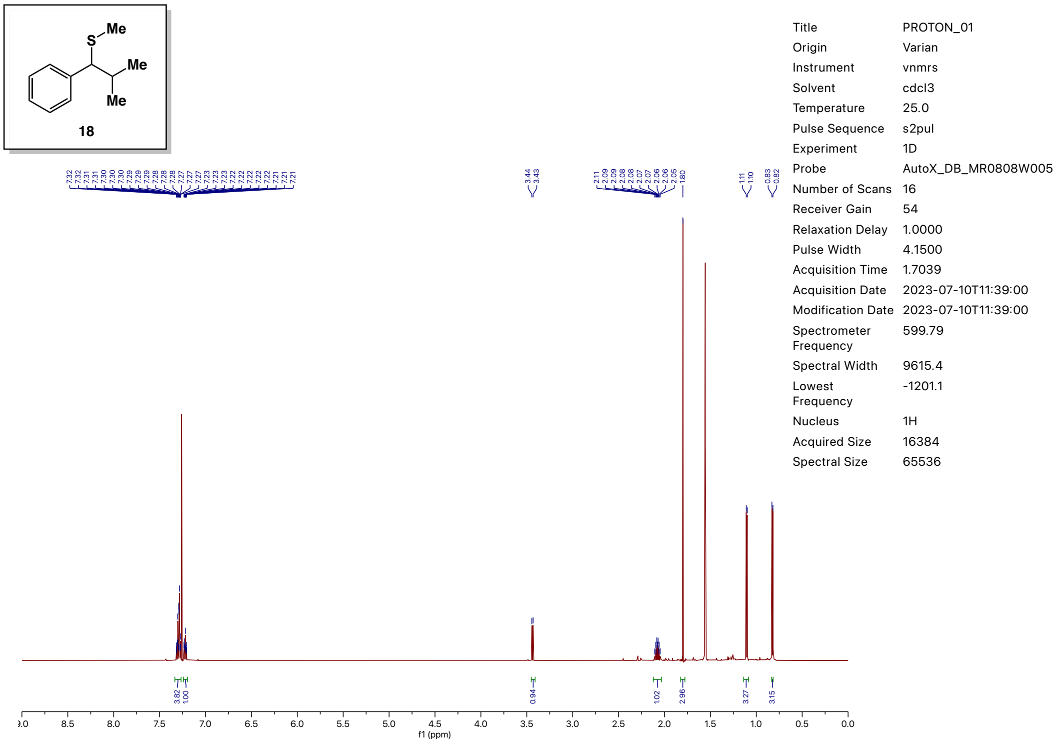

Fig. S26. ^{1}H NMR spectrum of thioether **18**.

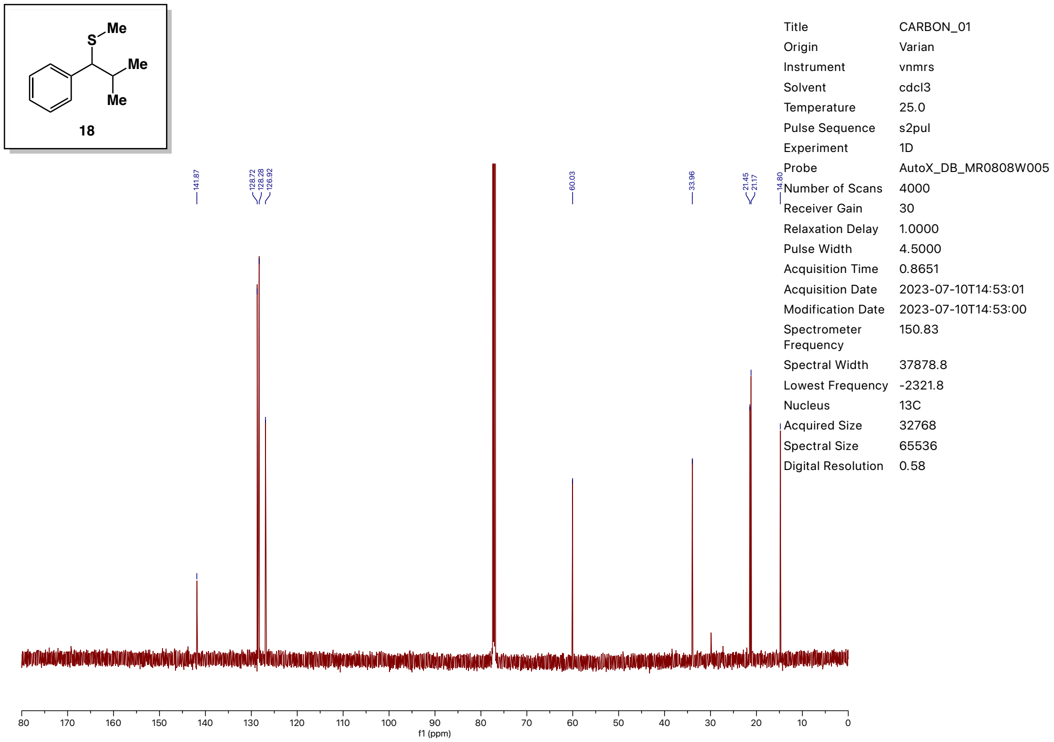

Fig. S27. ^{13}C NMR spectrum of thioether **18**.

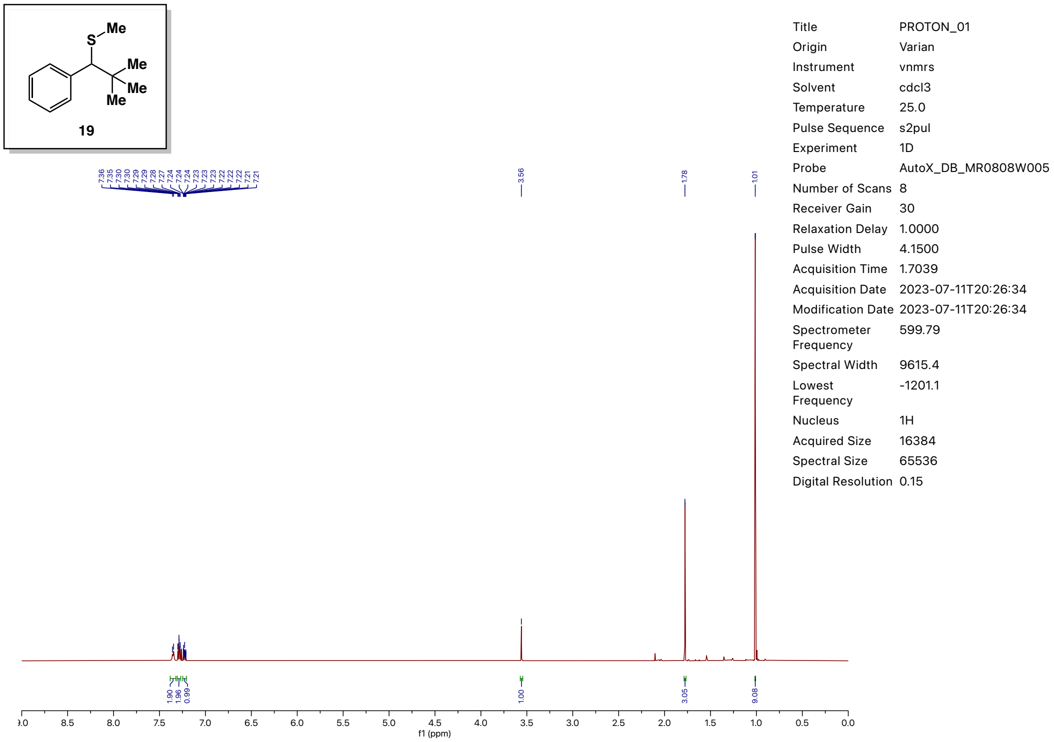

Fig. S28. ^{1}H NMR spectrum of thioether **19**.

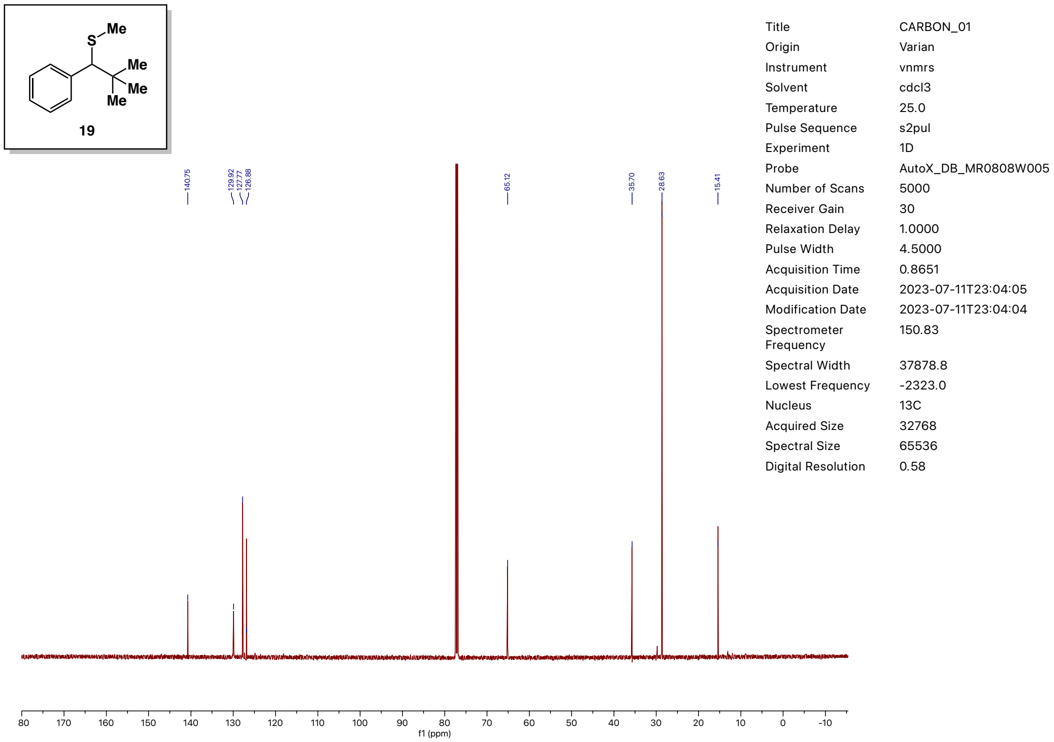

Fig. S29. ^{13}C NMR spectrum of thioether **19**.

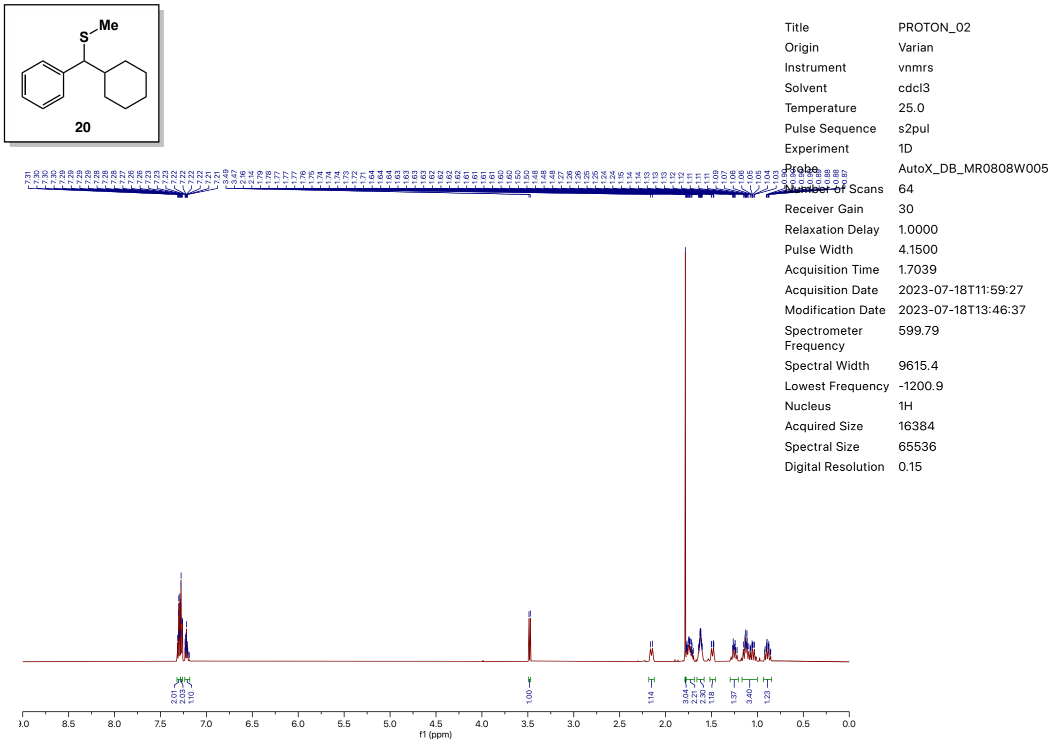

Fig. S30. ^{1}H NMR spectrum of thioether **20**.

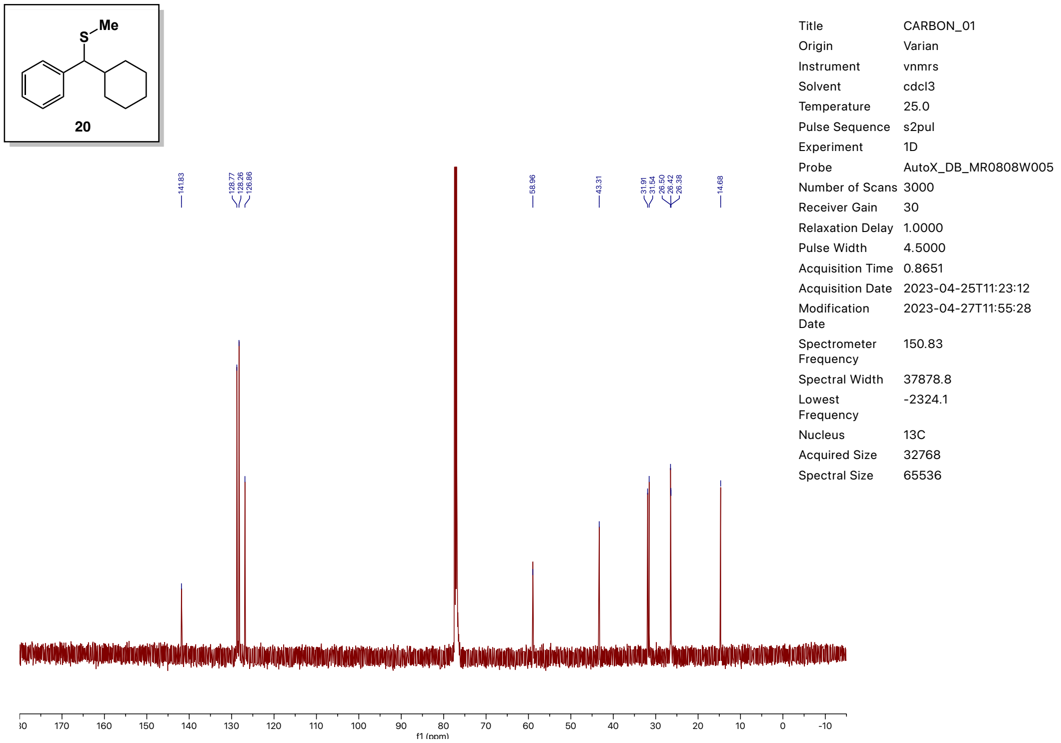

Fig. S31. ^{13}C NMR spectrum of thioether **20**.

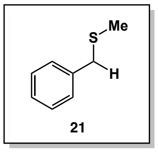

Title	PROTON_01
Origin	Varian
Instrument	vnmrs
Solvent	cdcl3
Temperature	25.0
Pulse Sequence	s2pul
Experiment	1D
Probe	AutoX_DB_MR0808W005
Number of Scans	32
Receiver Gain	42
Relaxation Delay	1.0000
Pulse Width	4.1500
Acquisition Time	1.7039
Acquisition Date	2023-04-25T16:38:32
Modification Date	2023-04-27T11:54:42
Spectrometer Frequency	599.79
Spectral Width	9615.4
Lowest Frequency	-1201.4
Nucleus	1H
Acquired Size	16384
Spectral Size	65536
Digital Resolution	0.15

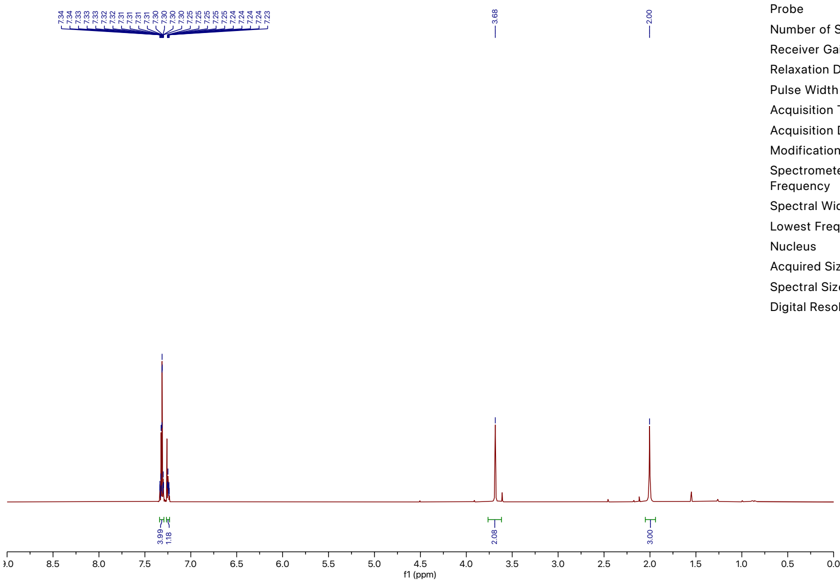

Fig. S32. ^{1}H NMR spectrum of thioether **21**.

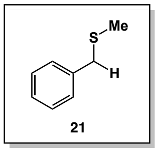

Title	CARBON_01
Origin	Varian
Instrument	vnmrs
Solvent	cdcl3
Temperature	25.0
Pulse Sequence	s2pul
Experiment	1D
Probe	AutoX_DB_MR0808W005
Number of Scans	4000
Receiver Gain	30
Relaxation Delay	1.0000
Pulse Width	4.5000
Acquisition Time	0.8651
Acquisition Date	2023-04-25T18:42:59
Modification Date	2023-04-27T11:54:43
Spectrometer Frequency	150.83
Spectral Width	37878.8
Lowest Frequency	-2324.7
Nucleus	13C
Acquired Size	32768
Spectral Size	65536
Digital Resolution	0.58

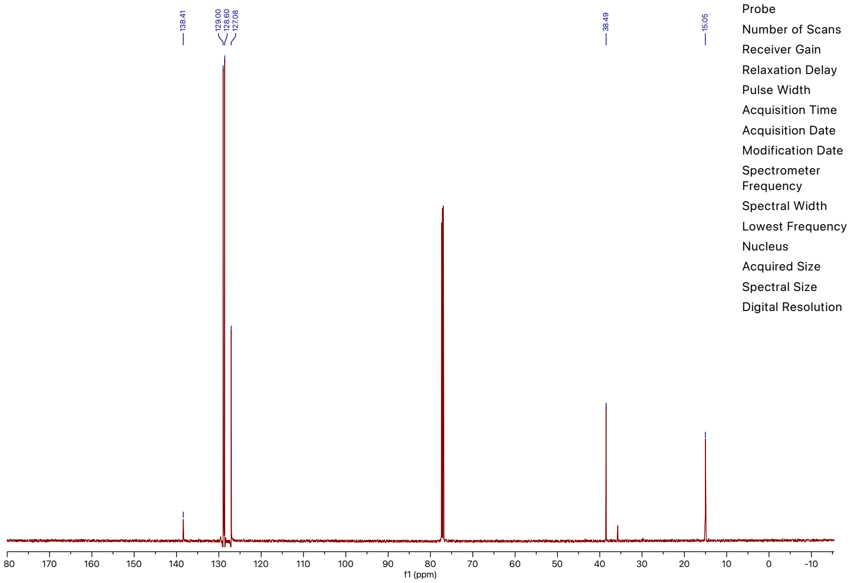

Fig. S33. ^{13}C NMR spectrum of thioether **21**.

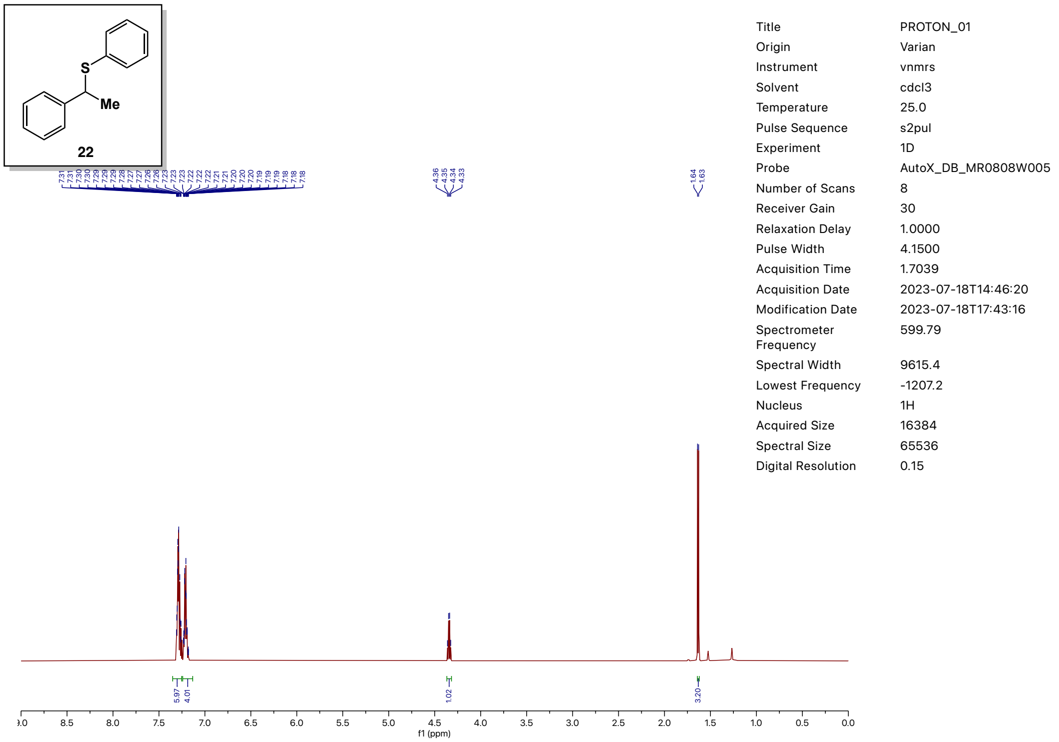

Fig. S34. ^{1}H NMR spectrum of thioether **22**.

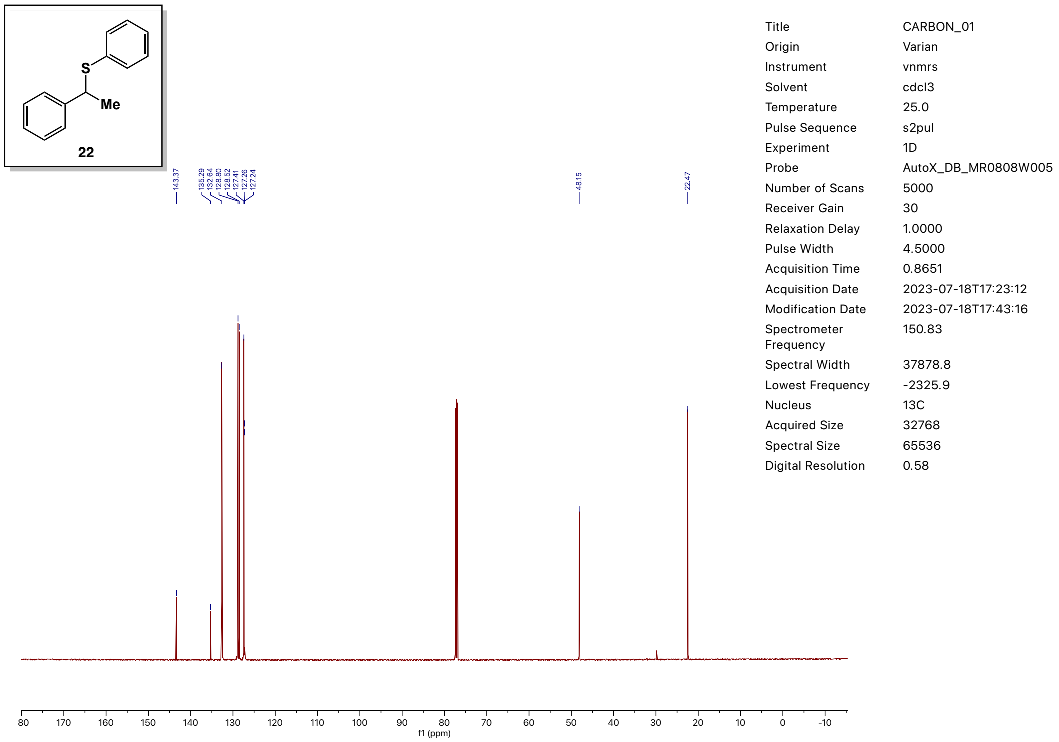

Fig. S35. ^{13}C NMR spectrum of thioether **22**.

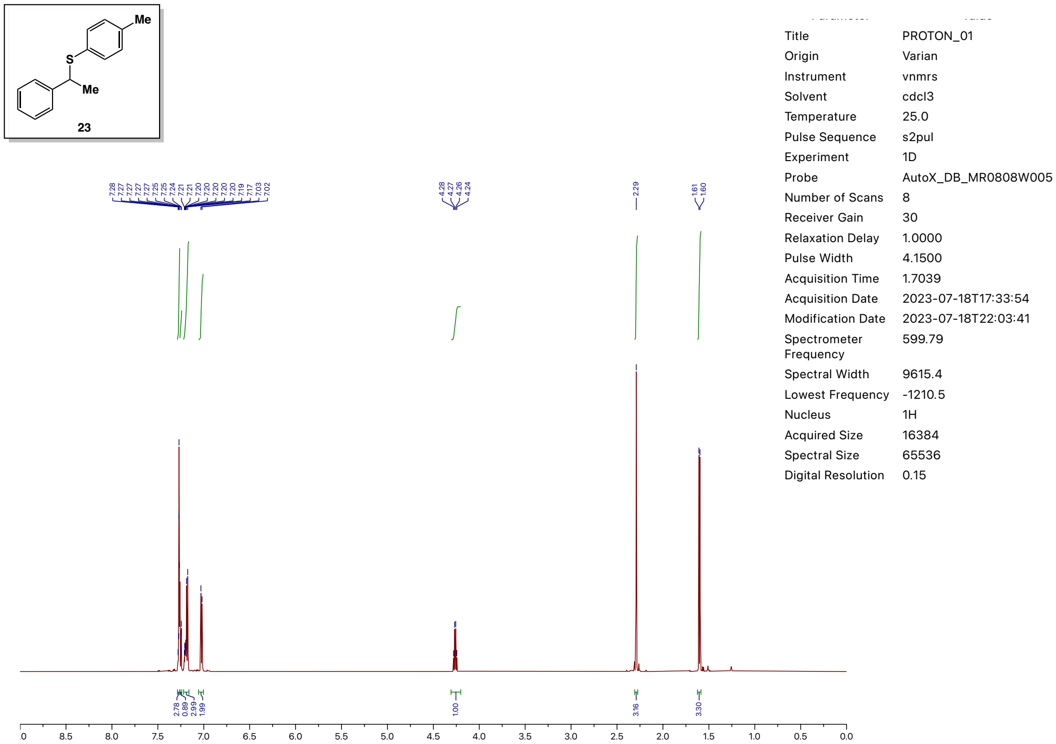

Fig. S36. ^{1}H NMR spectrum of thioether **23**.

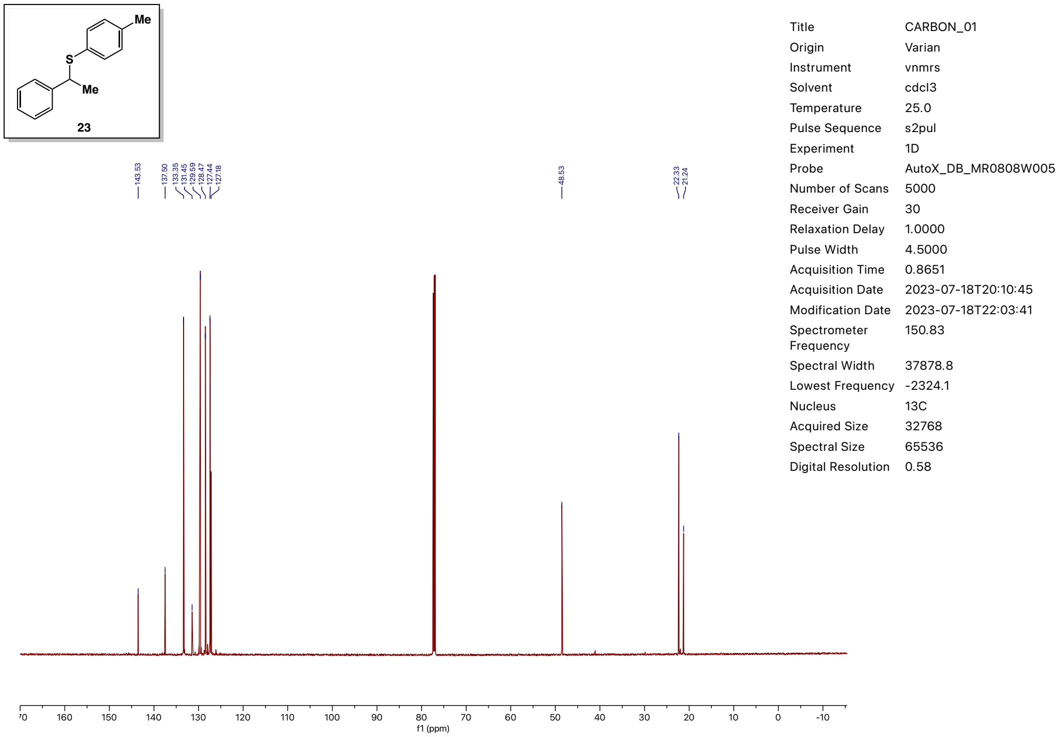

Fig. S37. ^{13}C NMR spectrum of thioether **23**.

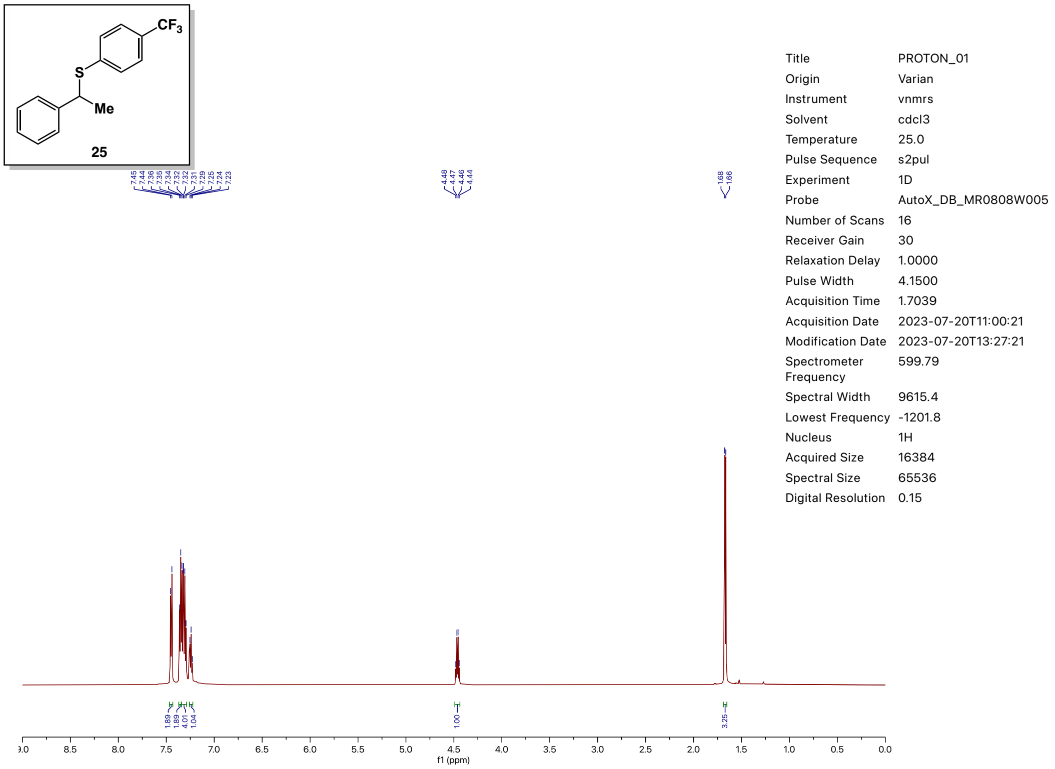

Fig. S38. ^{1}H NMR spectrum of thioether **25**.

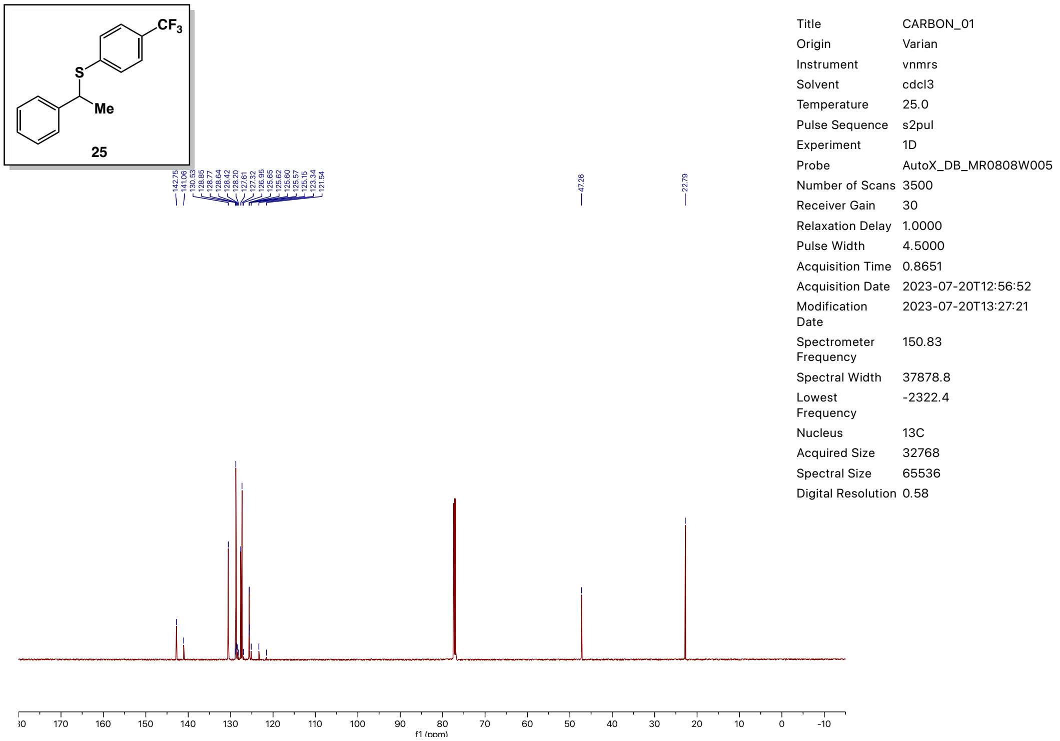

Fig. S39. ^{13}C NMR spectrum of thioether **25**.

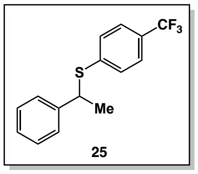

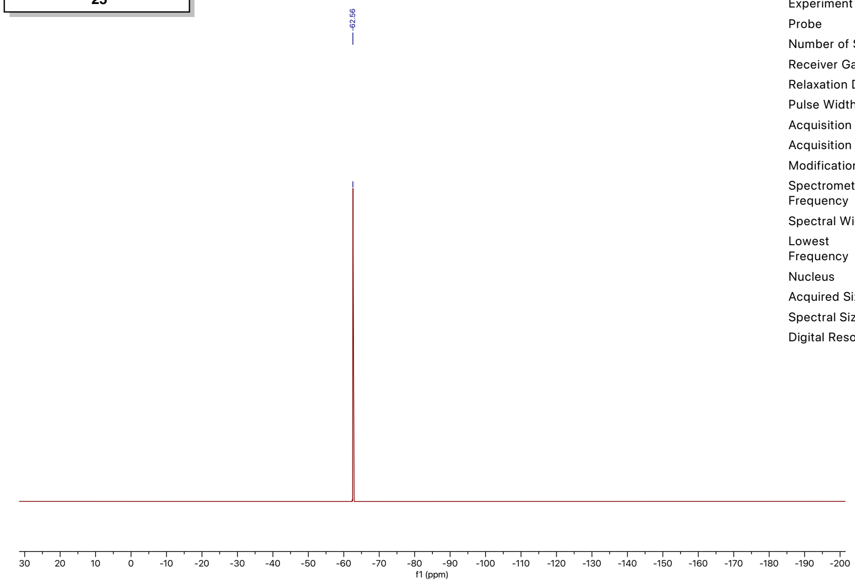

Title	FLUORINE_01
Origin	Varian
Instrument	vnmrs
Solvent	cdcl3
Temperature	25.0
Pulse Sequence	s2pul
Experiment	1D
Probe	AutoX_DB_MR0808W005
Number of Scans	32
Receiver Gain	54
Relaxation Delay	1.0000
Pulse Width	4.8667
Acquisition Time	0.9961
Acquisition Date	2023-07-20T11:04:58
Modification Date	2023-07-20T13:27:21
Spectrometer Frequency	564.32
Spectral Width	131578.9
Lowest Frequency	-113760.7
Nucleus	19F
Acquired Size	131072
Spectral Size	262144
Digital Resolution	0.50

Fig. S40. ^{19}F NMR spectrum of thioether **25**.

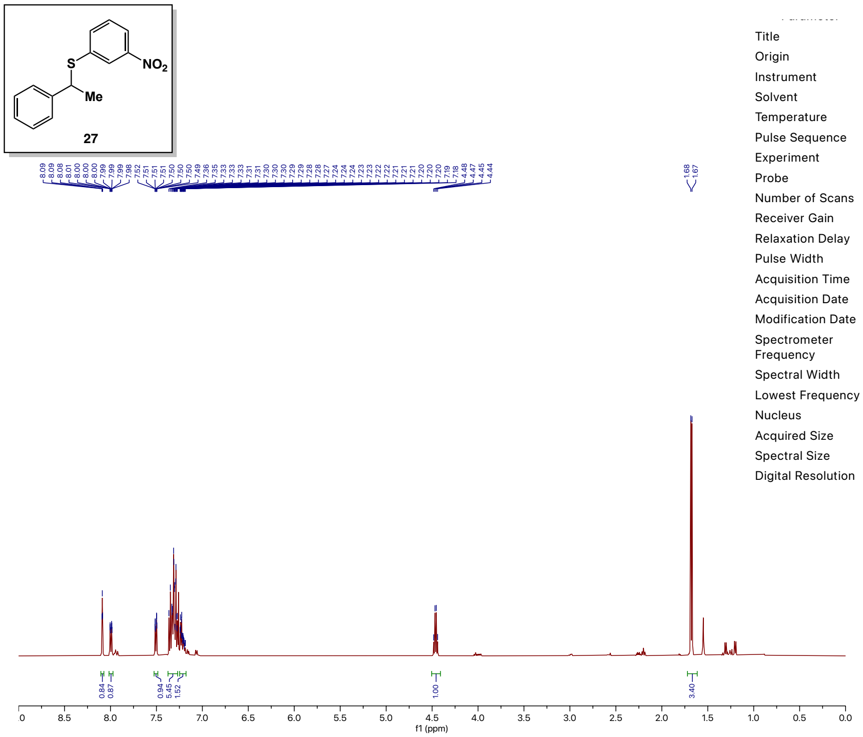

Parameter	Value
Title	PROTON_01
Origin	Varian
Instrument	vnmrs
Solvent	cdcl3
Temperature	25.0
Pulse Sequence	s2pul
Experiment	1D
Probe	5mm_MR0908W026_OneNMR
Number of Scans	16
Receiver Gain	30
Relaxation Delay	1.0000
Pulse Width	4.0000
Acquisition Time	2.0447
Acquisition Date	2023-07-28T18:05:19
Modification Date	2023-07-28T18:34:17
Spectrometer Frequency	499.76
Spectral Width	8012.8
Lowest Frequency	-1014.7
Nucleus	1H
Acquired Size	16384
Spectral Size	65536
Digital Resolution	0.12

Fig. S41. ^{1}H NMR spectrum of thioether **27**.

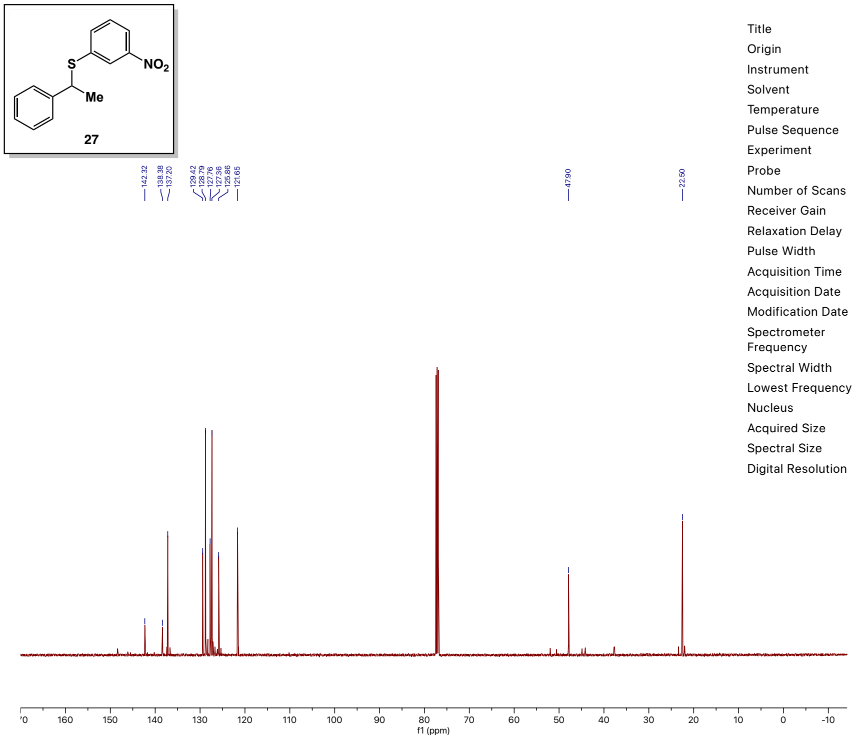

Title	CARBON_01
Origin	Varian
Instrument	vnmrs
Solvent	cdcl3
Temperature	25.0
Pulse Sequence	s2pul
Experiment	1D
Probe	5mm_MR0908W026_OneNMR
Number of Scans	5000
Receiver Gain	30
Relaxation Delay	1.0000
Pulse Width	4.3750
Acquisition Time	1.0486
Acquisition Date	2023-07-29T16:41:08
Modification Date	2023-07-29T17:19:43
Spectrometer Frequency	125.68
Spectral Width	31250.0
Lowest Frequency	-1783.6
Nucleus	13C
Acquired Size	32768
Spectral Size	65536
Digital Resolution	0.48

Fig. S42. ^{13}C NMR spectrum of thioether **27.**

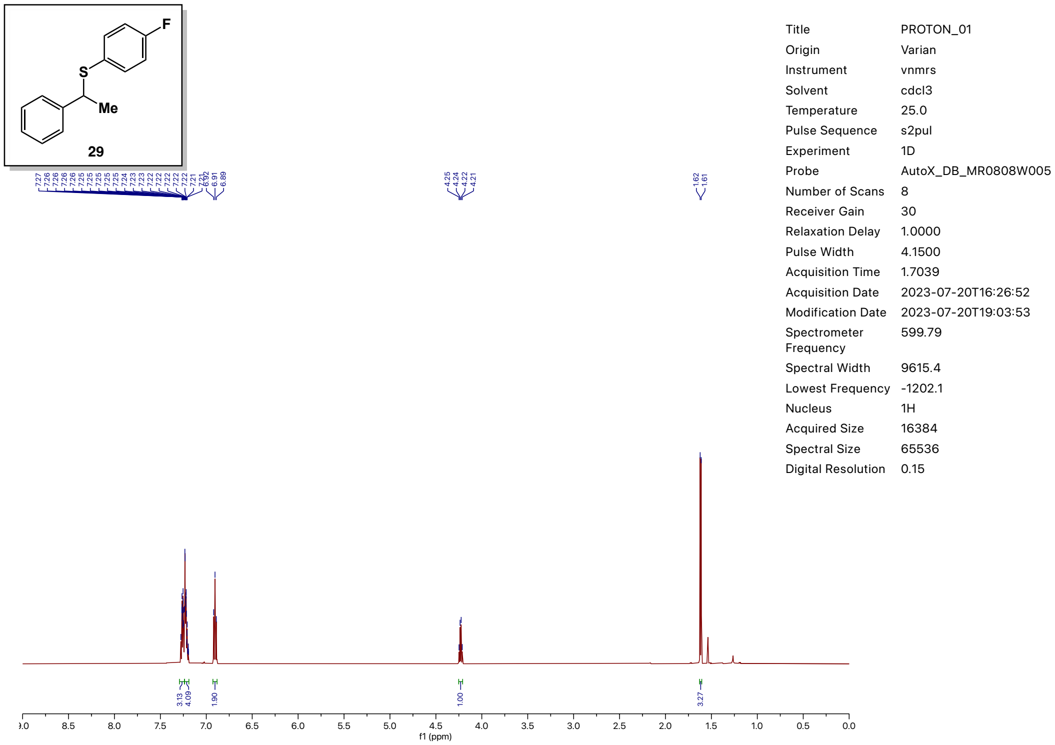

Fig. S43. ^{1}H NMR spectrum of thioether **29**.

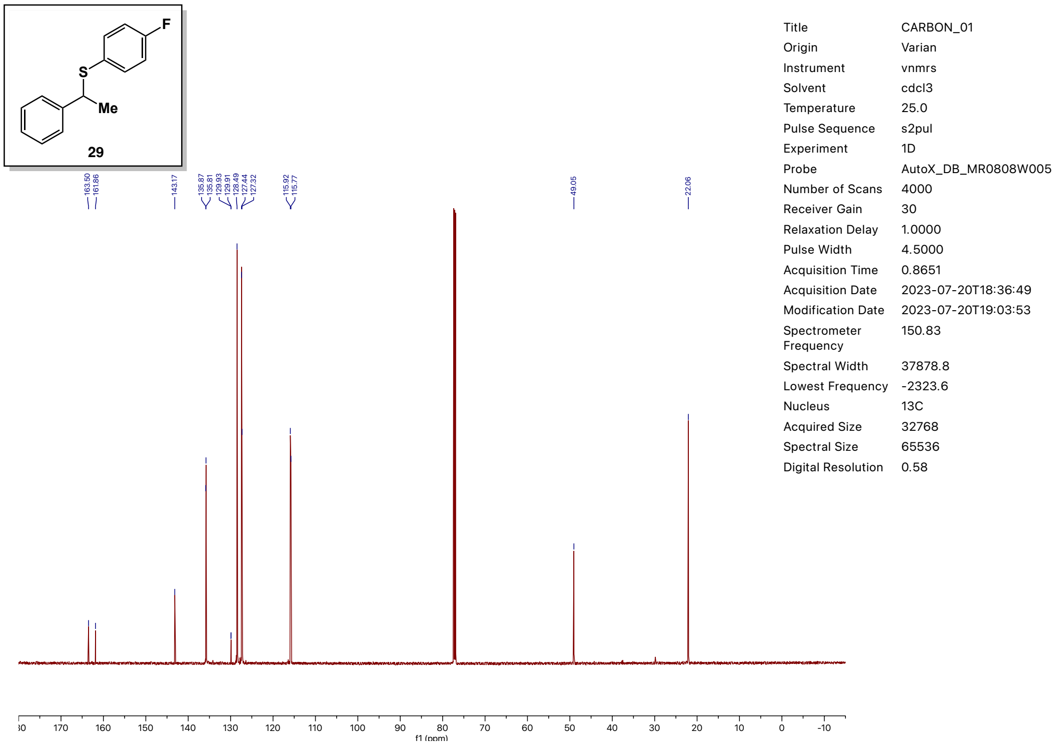

Fig. S44. ^{13}C NMR spectrum of thioether **29.**

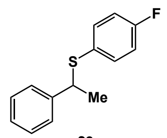

29

Title	FLUORINE_01
Origin	Varian
Instrument	vnmrs
Solvent	cdcl3
Temperature	25.0
Pulse Sequence	s2pul
Experiment	1D
Probe	AutoX_DB_MR0808W005
Number of Scans	32
Receiver Gain	44
Relaxation Delay	1.0000
Pulse Width	4.8667
Acquisition Time	0.9961
Acquisition Date	2023-07-20T16:30:23
Modification Date	2023-07-20T19:03:56
Spectrometer Frequency	564.32
Spectral Width	131578.9
Lowest Frequency	-113760.7
Nucleus	19F
Acquired Size	131072
Spectral Size	262144
Digital Resolution	0.50

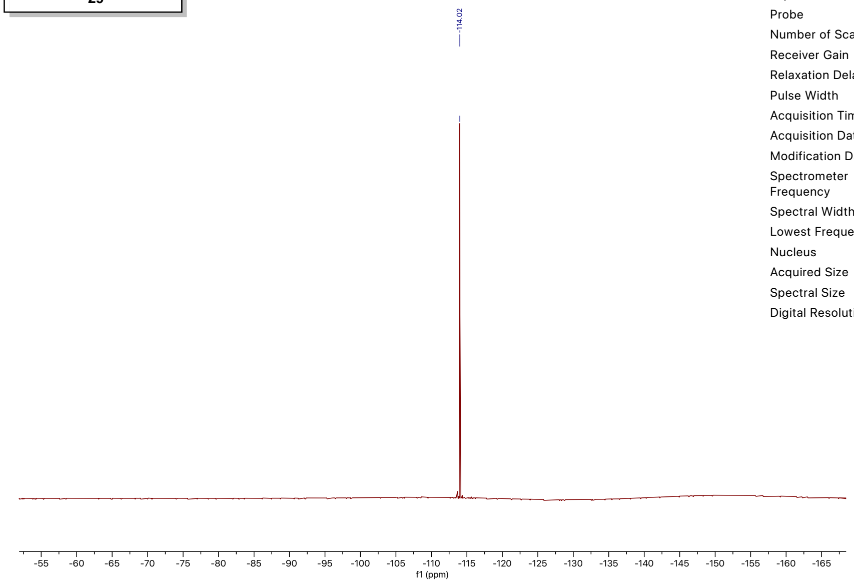

Fig. S45. ^{19}F NMR spectrum of thioether **29**.

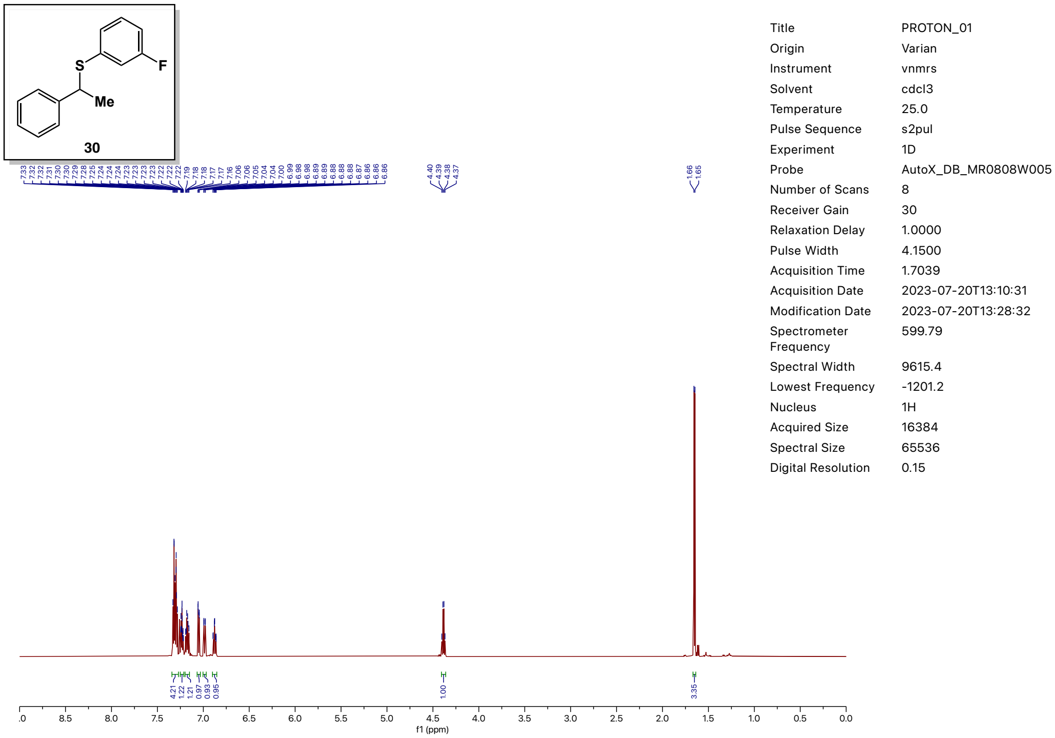

Fig. S46. ^{1}H NMR spectrum of thioether **30**.

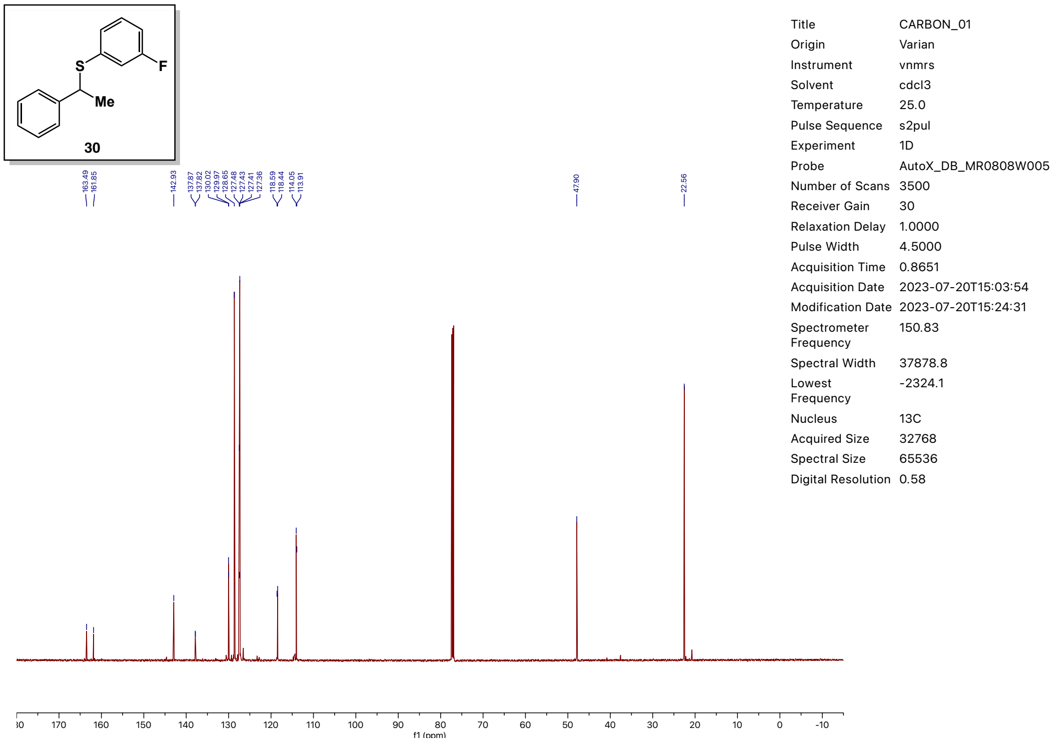

Fig. S47. ^{13}C NMR spectrum of thioether **30.**

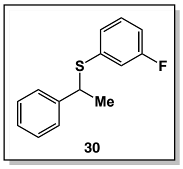

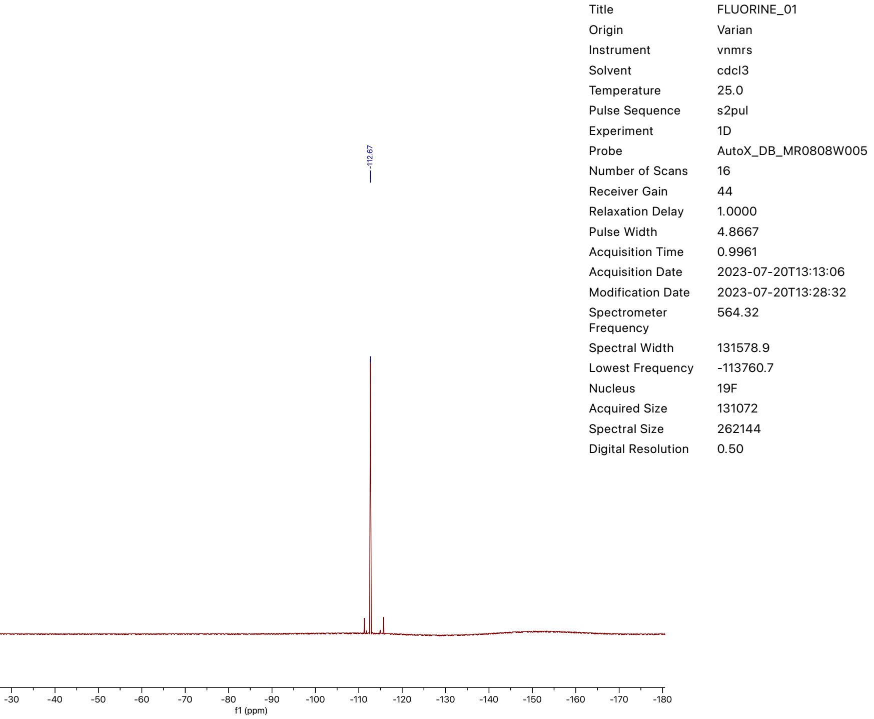

Fig. S48. ^{19}F NMR spectrum of thioether **30**.

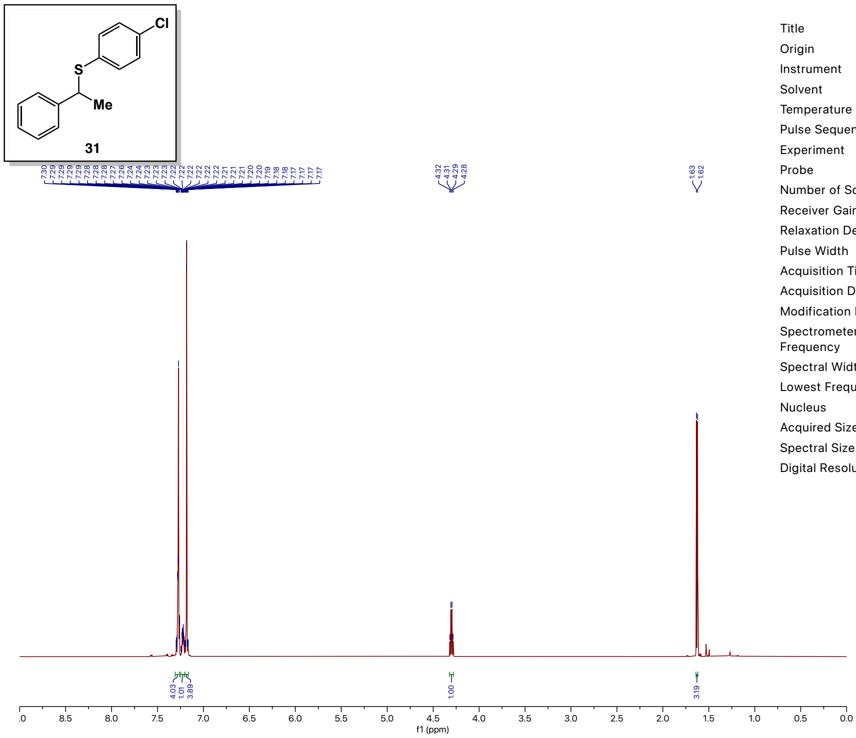

Title	PROTON_01
Origin	Varian
Instrument	vnmrs
Solvent	cdcl3
Temperature	25.0
Pulse Sequence	s2pul
Experiment	1D
Probe	AutoX_DB_MR0808W005
Number of Scans	8
Receiver Gain	30
Relaxation Delay	1.0000
Pulse Width	4.1500
Acquisition Time	1.7039
Acquisition Date	2023-07-18T20:24:13
Modification Date	2023-07-19T11:12:10
Spectrometer Frequency	599.79
Spectral Width	9615.4
Lowest Frequency	-1200.9
Nucleus	1H
Acquired Size	16384
Spectral Size	65536
Digital Resolution	0.15

Fig. S49. ^{1}H NMR spectrum of thioether **31**.

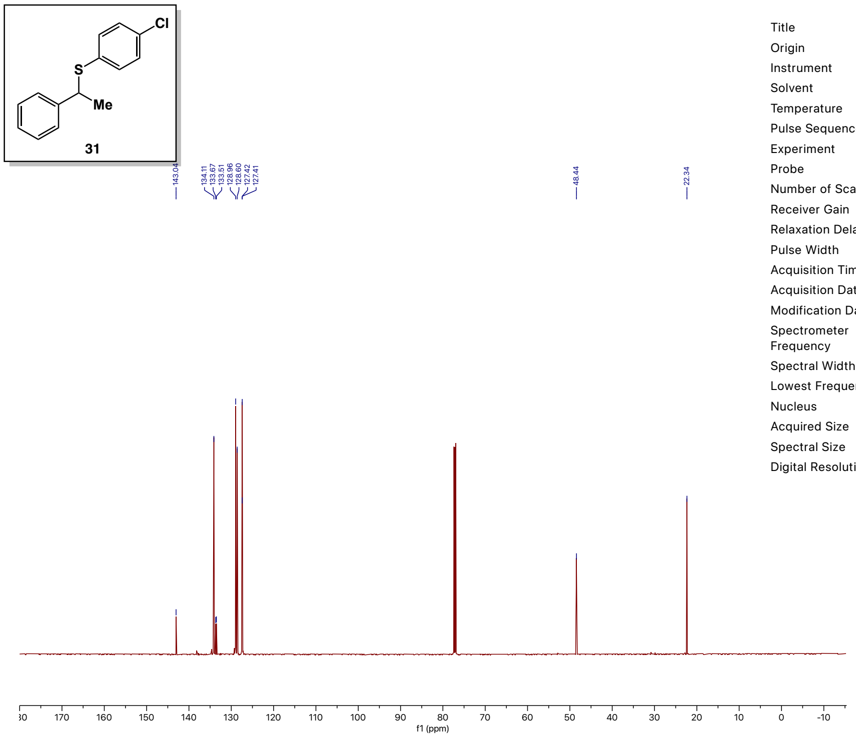

Title	CARBON_01
Origin	Varian
Instrument	vnmrs
Solvent	cdcl3
Temperature	25.0
Pulse Sequence	s2pul
Experiment	1D
Probe	AutoX_DB_MR0808W005
Number of Scans	5000
Receiver Gain	30
Relaxation Delay	1.0000
Pulse Width	4.5000
Acquisition Time	0.8651
Acquisition Date	2023-07-18T23:01:38
Modification Date	2023-07-19T11:12:11
Spectrometer Frequency	150.83
Spectral Width	37878.8
Lowest Frequency	-2322.6
Nucleus	13C
Acquired Size	32768
Spectral Size	65536
Digital Resolution	0.58

Fig. S50. ^{13}C NMR spectrum of thioether **31.**

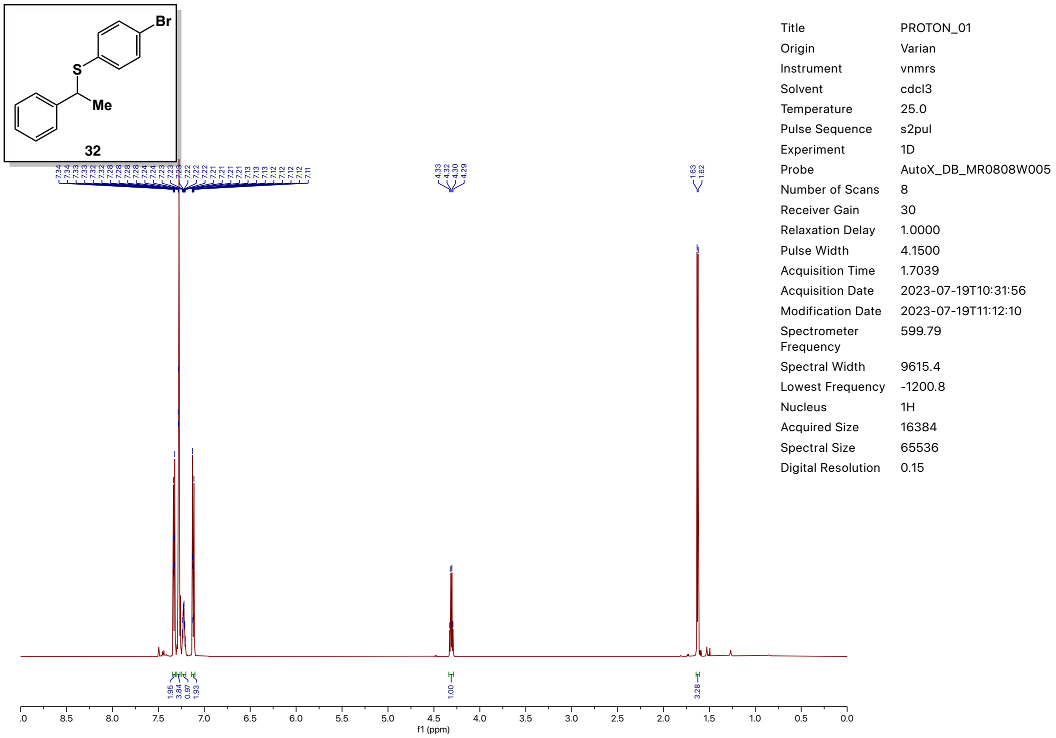

Fig. S51. ^{1}H NMR spectrum of thioether **32**.

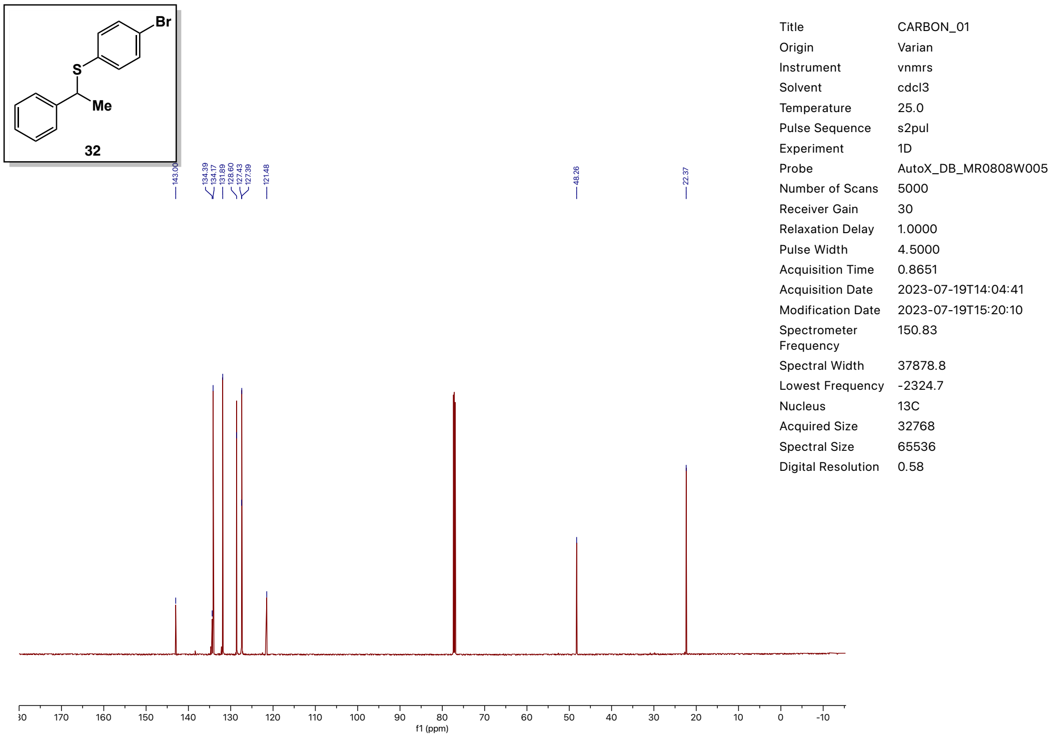

Fig. S52. ^{13}C NMR spectrum of thioether **32.**

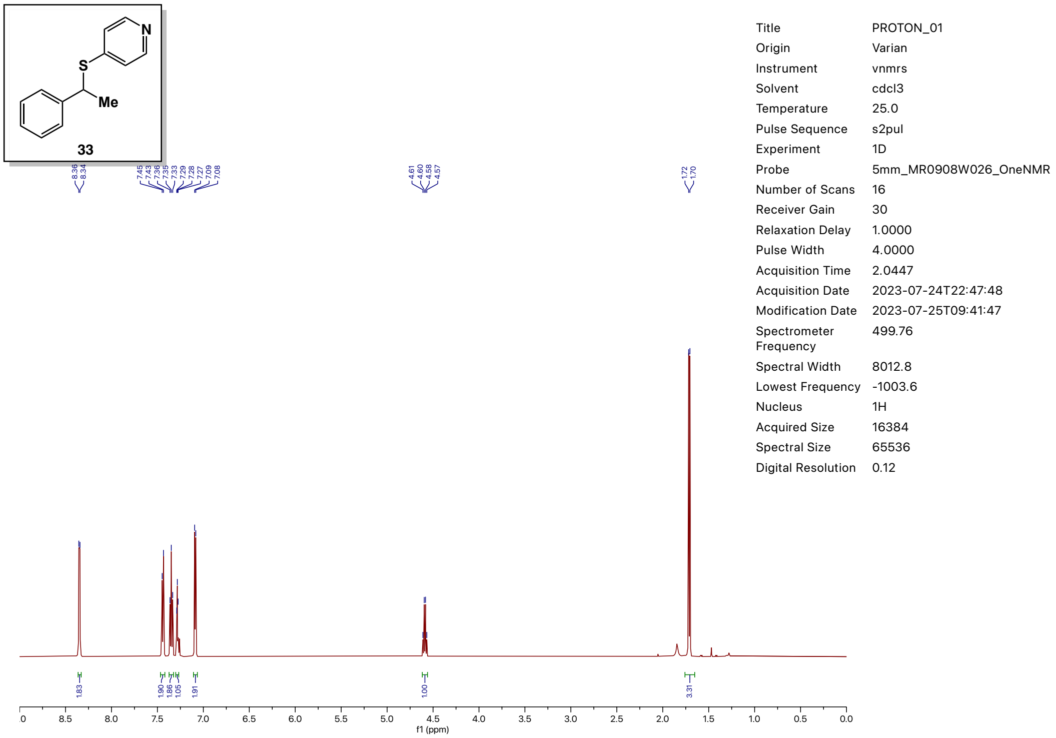

Fig. S53. ^{1}H NMR spectrum of thioether **33**.

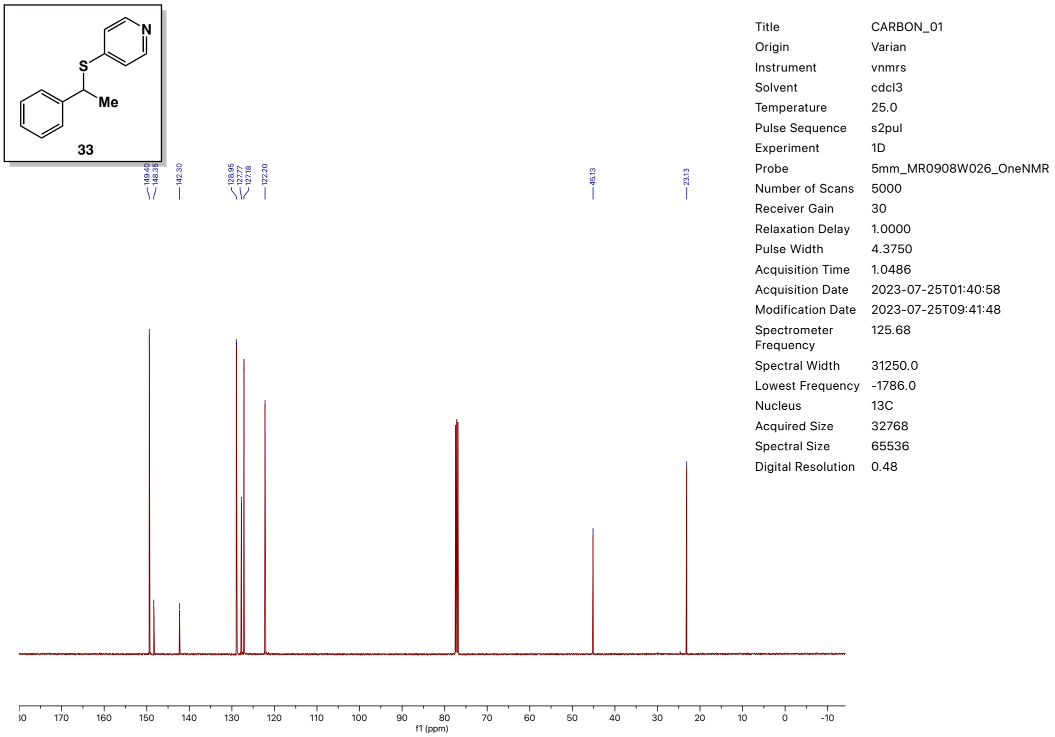

Fig. S54. ^{13}C NMR spectrum of thioether **33**.

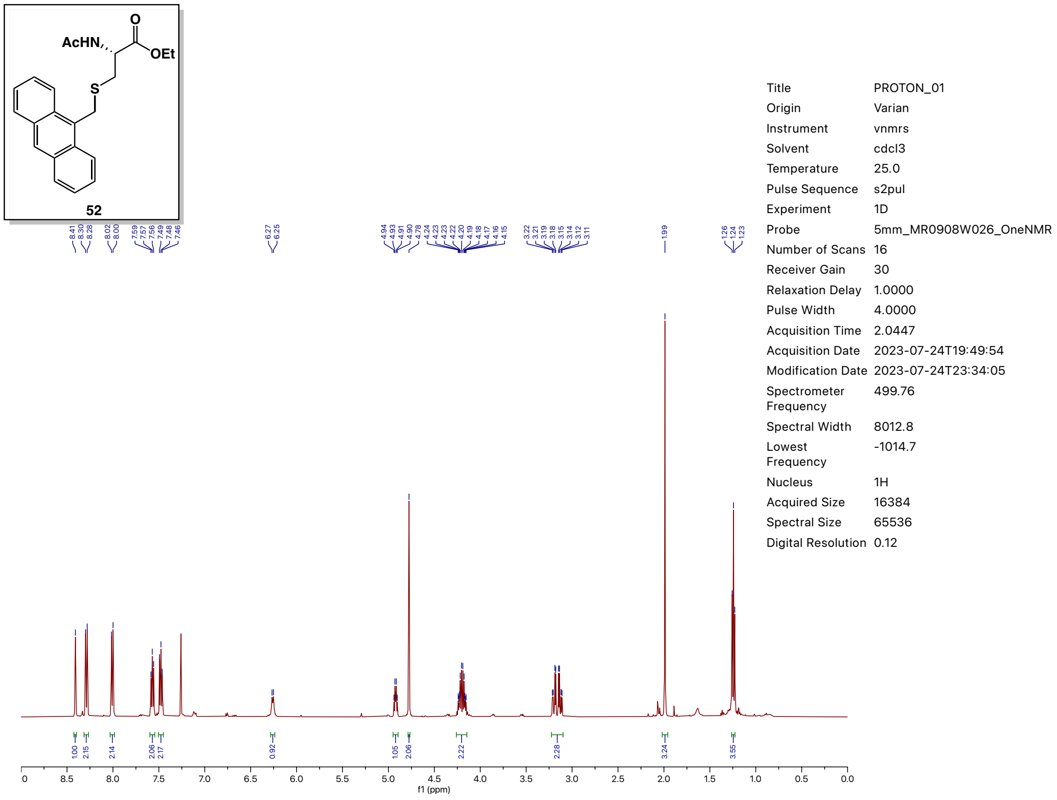

Fig. S55. ^{1}H NMR spectrum of thioether **52**.

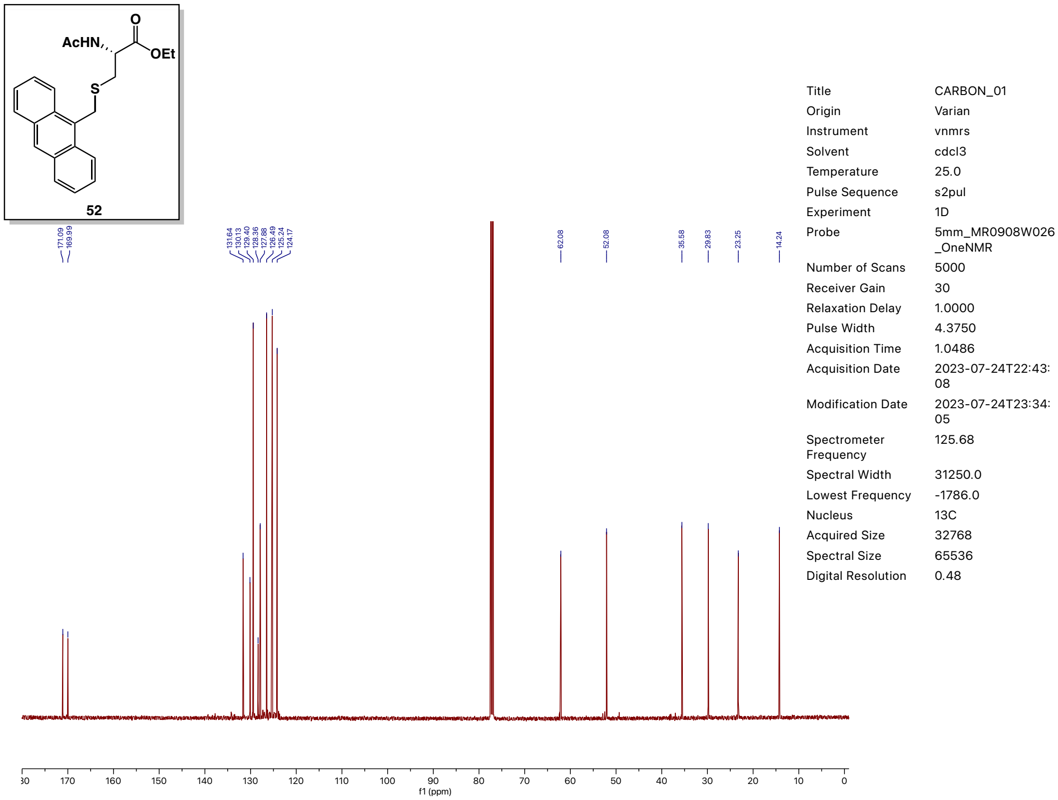

Fig. S56. ^{13}C NMR spectrum of thioether **52.**

14. SFC data

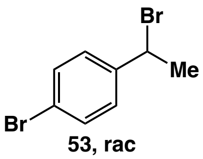

53, rac

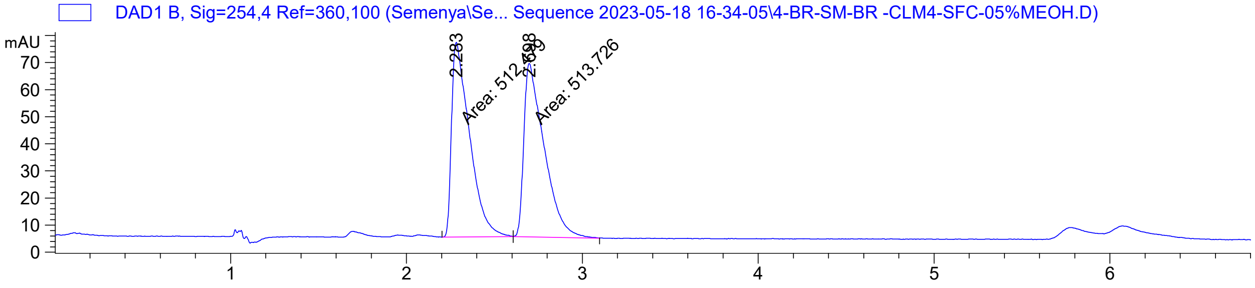

Signal 2: DAD1 B, Sig=254,4 Ref=360,100

Peak #	RetTime [min]	Type	Width [min]	Area [mAU*s]	Height [mAU]	Area %
1	2.283	MM	0.1188	512.47937	71.89185	49.9393
2	2.698	MM	0.1337	513.72620	64.01808	50.0607

Totals : 1026.20557 135.90993

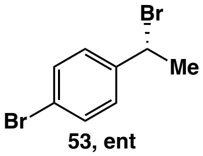

53, ent

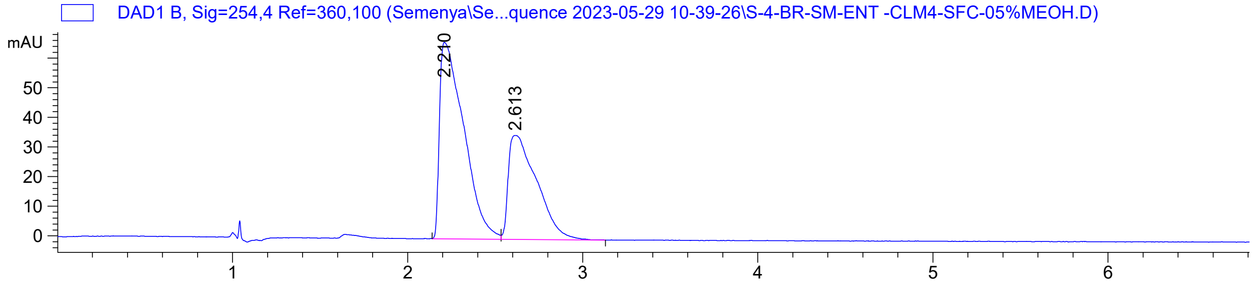

Signal 2: DAD1 B, Sig=254,4 Ref=360,100

Peak #	RetTime [min]	Type	Width [min]	Area [mAU*s]	Height [mAU]	Area %
1	2.210	BV	0.1350	639.29968	66.30545	61.7159
2	2.613	VV R	0.1455	396.57468	35.03807	38.2841

Totals : 1035.87436 101.34352

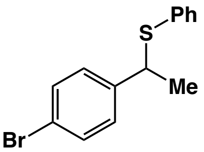

55, rac

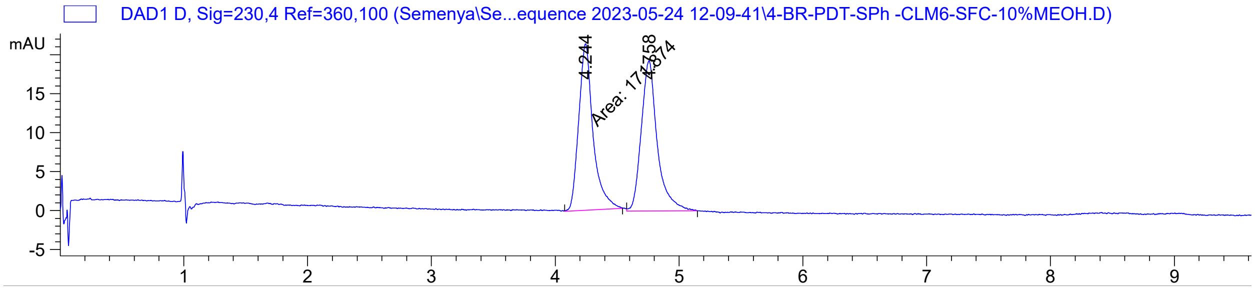


```
Signal 4: DAD1 D, Sig=230,4 Ref=360,100

Peak RetTime Type  Width     Area       Height     Area
  #    [min]        [min]    [mAU*s]     [mAU]       %
----|-------|----|-------|----------|----------|--------|
   1   4.244 MM    0.1341  171.87413   21.35632  49.8170
   2   4.758 VV R  0.1209  173.13667   19.25885  50.1830

Totals :                   345.01080   40.61517
```

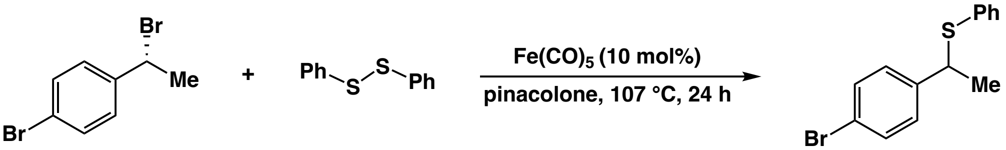

53 **24% ee** **54** **55** **0% ee**

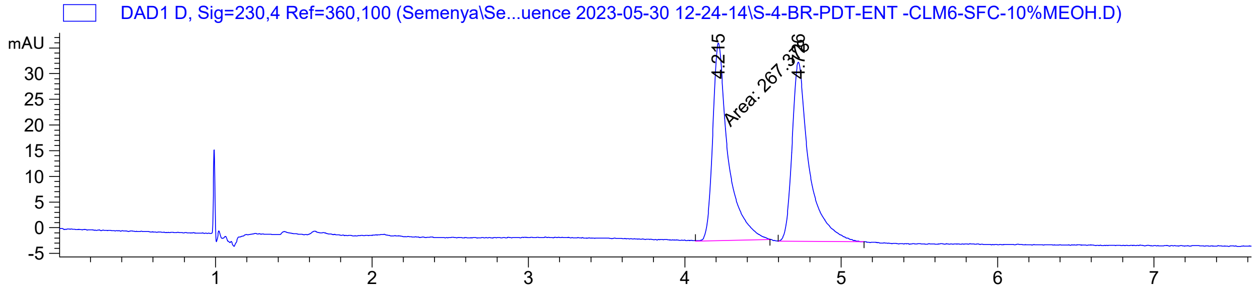


```
Signal 4: DAD1 D, Sig=230,4 Ref=360,100

Peak RetTime Type  Width     Area       Height     Area
  #    [min]        [min]    [mAU*s]     [mAU]       %
----|-------|----|-------|----------|----------|--------|
   1   4.215 MM    0.1159  267.37823   38.45855  50.1115
   2   4.726 BB    0.1096  266.18866   34.87076  49.8885

Totals :                   533.56689   73.32931
```

15. References

1. Ouyang, W.; Cai, X.; Chen, X.; Wang, J.; Rao, J.; Huo, Y.; Chen, Q.; Li, X. Sequential C–H Activation Enabled Expedient Delivery for Polyfunctional Arenes. *Chem. Commun.* **2021**, *57*, 8075–8078. DOI: 10.1039/D1CC03243G
2. Niu, B.; Sachidanandan, K.; Cooke, M. V.; Casey, T. E.; Laulhe, S. Photoinduced C(sp^3)–H Chalcogenation of Amide Derivatives and Ethers via Ligand-to-Metal Charge-Transfer. *Org. Lett.* **2022**, *24*, 4524–4529. DOI: 10.1021/acs.orglett.2c01505
3. Boehm, P.; Muller, P.; Finkelstein, P.; Rivero–Crespo, M. A.; Ebert, M–O.; Trapp, N.; Morandi, B. Mechanistic Investigation of the Nickel-Catalyzed Metathesis between Aryl Thioethers and Aryl Nitriles. *J. Am. Chem. Soc.* **2022**, *144*, 13096–13108. DOI: 10.1021/jacs.2c01595
4. Leino, R.; Lonnqvist, J-K. A very Simple Method for the Preparation of Symmetrical Disulfides. *Tetrahedron Lett.* **2004**, *45*, 8489–8491. DOI: 10.1016/j.tetlet.2004.09.100
5. Zhu, C.; Wu, D.; Liu, H.; Meng, C.; Tang, T. Transformation of Thiols to Disulfides via an Oxidant-free Radical Pathway on the Zeolite ETS-10. *Green Chem.* **2022**, *24*, 9033–9039. DOI: 10.1039/D2GC02894H
6. Dong, X. Y.; Zhang, Y. F.; Ma, C. L.; Gu, Q. S.; Wang, F. L.; Li, Z. L.; Jiang, S. P.; Liu, X. Y. A General Asymmetric Copper-Catalysed Sonogashira C(sp3)–C(sp) Coupling. *Nat. Chem.* **2019**, *11*, 1158–1166. DOI: 10.1038/s41557-019-0346-2
7. Nielsen, A. J.; Raez–Villanueva, S.; Crankshaw, D. J.; Holloway, A. C; McNulty, J. Synthesis of α-Methylstilbenes using an Aqueous Wittig Methodology and Application toward the Development of Potent Human Aromatase Inhibitors. *Bioorg. Med. Chem. Lett. Sci.* **2019**, *11*, 1395–1398. DOI: 10.1016/j.bmcl.2019.03.033
8. Holz, J.; Pfeffer, C.; Zuo, H.; Beierlein, D.; Richter, G.; Klemm, E.; Peters, R. In Situ Generated Gold Nanoparticles on Active Carbon as Reusable Highly Efficient Catalysts for a C−C Stille Coupling. *Angew. Chem. Int. Ed.* **2019**, *58*, 10330–10334. DOI: 10.1002/anie.201902352
9. Wang, F.; Nishimoto, Y.; Yasuda, M. Insertion of Diazo Esters into C–F Bonds toward Diastereoselective One-Carbon Elongation of Benzylic Fluorides: Unprecedented BF_3 Catalysis with C–F Bond Cleavage and Re-formation. *J. Am. Chem. Soc.* **2021**, *143*, 20616–20621. DOI: 10.1021/jacs.1c10517
10. Lopez, M. A.; Buss, J. A.; Stahl, S. S. Cu-Catalyzed Site-Selective Benzylic Chlorination Enabling Net C–H Coupling with Oxidatively Sensitive Nucleophiles. *Org. Lett.* **2022**, *24*, 597–601. DOI: 10.1021/acs.orglett.1c04038
11. Bandgar, B. P.; Sadavarte, V. S.; Uppalla, L. S. An expedient and Highly Selective Iodination of Alcohols using a $KI/BF_3{\cdot}Et_2O$ System. *Tetrahedron Lett.* **2001**, *42*, 951–953. DOI: 10.1016/S0040-4039(00)01953-5
12. Li, S.; Chen, T.; Saga, Y.; Han, L. B. Chloroform-based Atherton–Todd-type Reactions of Alcohols and Thiols with Secondary Phosphine oxides Generating Phosphinothioates and Phosphinates. *RSC. Adv.* **2015**, *5*, 71544–71546. D DOI: 10.1039/C5RA16015D
13. Liu, C. Y.; Pawar, V. D.; Kao, J. Q.; Chen, C. T. Substitution- and Elimination-Free Phosphorylation of Functionalized Alcohols Catalyzed by Oxidomolybdenum Tetrachloride. *Adv. Synth. Catal.* **2010**, *352*, 188–194. DOI: 10.1002/adsc.200900279
14. Hsiao, Y-T.; Beadle, J.; Pascoe, C.; Annadate, R.; Vederas, J. C. Decarboxylative Radical Addition to Methylideneoxazolidinones for Stereocontrolled Synthesis of Selectively Protected Diamino Diacids. *Org. Lett.* **2021**, *23*, 7270–7273. DOI: 10.1021/acs.orglett.1c02684

15. Taniguchi, N. Bronsted Acid-Assisted Zinc-Catalyzed Markovnikov-Type Hydrothiolation of Alkenes Using Thiols. *J. Org. Chem.* **2020**, *85*, 6528–6534. DOI: 10.1021/acs.joc.0c00487
16. Gui, Y.; Tian, S. K. Stereospecific Nucleophilic Substitution of Enantioenriched Tertiary Benzylic Amines via in Situ Activation with Benzyne. *Org. Lett.* **2017**, *19*, 1554–1557. DOI: 10.1021/acs.orglett.7b00365
17. Lu, G. P.; Cai, C. An Odorless, One-Pot Synthesis of Nitroaryl Thioethers via S_NAr Reactions through the in–situ Generation of S-Alkylisothiouronium Salts. *RSC Adv.* **2014**, *4*, 59990–59996. DOI: 10.1039/C4RA11490F
18. Xu, B.; Lin, Y.; Ye, Y.; Xu, L.; Xie, T.; Ye, X.-Y. Benzyl Thioether Formation Merging Copper Catalysis. *RSC Adv.* **2022**, *12*, 692–697. DOI: 10.1039/D1RA08015F
19. Johnson, M. G.; Gribble, M. W.; Houze, J. B.; Paras, N. A. Convenient Route to Secondary Sulfinates: Application to the Stereospecific Synthesis of α-*C*-Chiral Sulfonamides. *Org. Lett.* **2014**, *16*, 6248–6251. DOI: 10.1021/ol503208z
20. Gellert, B. A.; Kahlcke, N.; Feurer, M.; Roth, S. Triflic Acid Catalyzed Reductive Coupling Reactions of Carbonyl Compounds with O-, S-, and N-Nucleophiles. *Chem. Eur. J.* **2011**, *17*, 12203–12209. DOI: 10.1002/chem.201101819
21. Scholz, R.; Hellman, G.; Rohs, S.; Raabe, G.; Runsink, J.; Ozdemir, D.; Luche, O.; Heb, T.; Giesen, A. W.; Atodiresei, J.; Lindner, H. J.; Gais, H. J. Experimental and Theoretical Investigation of the Enantiomerization of Lithium α-tert-Butylsulfonyl Carbanion Salts and the Determination of Their Structures in Solution and in the Crystal. *Eur. J. Org. Chem.* **2010**, *24*, 4559–4587. DOI: 10.1002/ejoc.201000409
22. Fang, Y.; Yuan, M.; Zhang, J.; Zhang, L.; Jin, X.; Li, R.; Lin, J. An Efficient and Straightforward Route to Terminal Vinyl Sulfones via Palladium-Catalyzed Suzuki Reactions of α-Bromo Ethenylsulfones. *Tetrahedron Lett.* **2016**, *57*, 1460–1463. DOI: 10.1016/j.tetlet.2016.02.065
23. Xie, D.; Wang, Y.; Zhang, X.; Fu, Z.; Niu, D. Alkyl/Glycosyl Sulfoxides as Radical Precursors and Their Use in the Synthesis of Pyridine Derivatives. *Angew. Chem. Int. Ed.* **2022**, *61*, 1–7. DOI: 10.1002/anie.202204922
24. Niu, S-T.; Liu, H.; Xu, Y-J.; Lu, C-D. Diastereoselective α-Sulfenylation of N-tert-Butanesulfinyl Imidates. *J. Org. Chem.* **2018**, *83*, 10580–10588. DOI: 10.1021/acs.joc.8b01403
25. Pandey, A. K.; Chand, S.; Sharma, A. K.; Singh, K. N. Copper-Catalyzed Thiolation of Hydrazones with Sodium Sulfinates: A Straightforward Synthesis of Benzylic Thioethers. *J. Org. Chem.* **2023**, *88*, 475–482. DOI: 10.1021/acs.joc.2c02451
26. Li, L.; Yu, Z.; Shen, Z. Copper-Catalyzed Aminoxylation of Different Types of Hydrocarbons with TEMPO: A Concise Route to N-Alkoxyamine Derivatives. *Adv. Synth. Catal.* **2015**, *357*, 3495–3500. DOI: 10.1002/adsc.201500544
27. Yamamoto, E.; Kawai, Y.; Takakura, K.; Kimura, M.; Murayama, H.; Matsueda, H.; Otsuki, S.; Sakata, H.; Tokunaga, M. Convenient Unsymmetrical Disulfane Synthesis: Basic Zeolite-Catalyzed Thiol-Disulfane Exchange Reaction. *ChemCatChem* **2021**, *13*, 4694–4699. DOI: 10.1002/cctc.202101092
